# Single-cell and spatial transcriptomics reveal a high glycolysis B cell and tumor-associated macrophages cluster correlated with poor prognosis and exhausted immune microenvironment in diffuse large B-cell lymphoma

**DOI:** 10.1186/s40364-024-00605-w

**Published:** 2024-06-05

**Authors:** Liyuan Dai, Guangyu Fan, Tongji Xie, Lin Li, Le Tang, Haizhu Chen, Yuankai Shi, Xiaohong Han

**Affiliations:** 1 National Cancer Center/National Clinical Research Center for Cancer/Cancer Hospital, Chinese Academy of Medical Sciences & Peking Union Medical College, Beijing Key Laboratory of Clinical Study on Anticancer Molecular Targeted Drugs, No. 17 Panjiayuan Nanli, Chaoyang District, Beijing, 100021 China; 2 Department of Medical Oncology, National Cancer Center/National Clinical Research Center for Cancer/Cancer Hospital, Chinese Academy of Medical Sciences & Peking Union Medical College, Beijing Key Laboratory of Clinical Study on Anticancer Molecular Targeted Drugs, No. 17 Panjiayuan Nanli, Chaoyang District, Beijing, 100021 China; 3 Department of Pathology, National Cancer Center/National Clinical Research Center for Cancer/Cancer Hospital, Chinese Academy of Medical Sciences & Peking Union Medical College, No. 17 Panjiayuan Nanli, Chaoyang District, Beijing, 100021 China; 4 Guangdong Provincial Key Laboratory of Malignant Tumor Epigenetics and Gene Regulation, Breast Tumor Centre, Department of Medical Oncology, Phase I Clinical Trial Centre, Sun Yat-sen Memorial Hospital, Sun Yat-sen University, Guangzhou, 510120 P. R. China; 5 Clinical Pharmacology Research Center, Peking Union Medical College Hospital, State Key Laboratory of Complex Severe and Rare Diseases, NMPA Key Laboratory for Clinical Research and Evaluation of Drug, Beijing Key Laboratory of Clinical PK & PD Investigation for Innovative Drugs, Chinese Academy of Medical Sciences & Peking Union Medical College, No.1, Shuaifuyuan, Dongcheng District, Beijing, 100730 China

**Keywords:** Single-cell transcriptomics, Spatial transcriptomics, Diffuse large B-cell lymphoma, Metabolism, Glycolysis

## Abstract

**Background:**

Diffuse large B-cell lymphoma (DLBCL) is a heterogeneous malignancy characterized by varied responses to treatment and prognoses. Understanding the metabolic characteristics driving DLBCL progression is crucial for developing personalized therapies.

**Methods:**

This study utilized multiple omics technologies including single-cell transcriptomics (*n* = 5), bulk transcriptomics (*n* = 966), spatial transcriptomics (*n* = 10), immunohistochemistry (*n* = 34), multiple immunofluorescence (*n* = 20) and to elucidate the metabolic features of highly malignant DLBCL cells and tumor-associated macrophages (TAMs), along with their associated tumor microenvironment. Metabolic pathway analysis facilitated by scMetabolism, and integrated analysis via hdWGCNA, identified glycolysis genes correlating with malignancy, and the prognostic value of glycolysis genes (*STMN1, ENO1, PKM*, and *CDK1*) and TAMs were verified.

**Results:**

High-glycolysis malignant DLBCL tissues exhibited an immunosuppressive microenvironment characterized by abundant IFN_TAMs (CD68^+^CXCL10^+^PD-L1^+^) and diminished CD8^+^ T cell infiltration. Glycolysis genes were positively correlated with malignancy degree. IFN_TAMs exhibited high glycolysis activity and closely communicating with high-malignancy DLBCL cells identified within datasets. The glycolysis score, evaluated by seven genes, emerged as an independent prognostic factor (*HR* = 1.796, *95% CI*: 1.077–2.995, *p* = 0.025 and *HR* = 2.631, *95% CI*: 1.207–5.735, *p* = 0.015) along with IFN_TAMs were positively correlated with poor survival (*p* < 0.05) in DLBCL. Immunohistochemical validation of glycolysis markers (*STMN1, ENO1, PKM*, and *CDK1*) and multiple immunofluorescence validation of IFN_TAMs underscored their prognostic value (*p* < 0.05) in DLBCL.

**Conclusions:**

This study underscores the significance of glycolysis in tumor progression and modulation of the immune microenvironment. The identified glycolysis genes and IFN_TAMs represent potential prognostic markers and therapeutic targets in DLBCL.

**Supplementary Information:**

The online version contains supplementary material available at 10.1186/s40364-024-00605-w.

## Introduction

Diffuse large B-cell lymphoma (DLBCL) is a highly aggressive and heterogeneous malignancy. Despite advancements in treatment outcomes with the R‐CHOP (rituximab plus cyclophosphamide, doxorubicin, vincristine, and prednisone) regimen, 40% of patients experience poor survival outcomes within 5 years [1]. In the current era of immunochemotherapy, the International Prognostic Index (IPI) and revised IPI scoring systems, which rely on clinical data, do not account for important prognostic factors such as cytogenetics, genomics, and molecular mechanisms [2]. This emphasizes the necessity to identify high-risk patients who may have poor responses to immunochemotherapy, necessitating the exploration of alternative treatment strategies.

The significance of metabolic pathways in the pathogenesis of malignant lymphoma has been extensively reported [3, 4]. Metabolic reprogramming, a key hallmark of cancer, often involves aerobic glycolysis, also known as the ‘Warburg effect’ [5]. Cancer cells exhibit a preference for aerobic glycolysis over oxidative phosphorylation for glucose metabolism, resulting in the generation of adenosine 5’-triphosphate less efficiently and the creation of a highly acidic microenvironment. This metabolic characteristic forms the basis for the clinical utility of fluorodeoxyglucose positron emission tomography computed tomography (FDG-PET/CT) imaging [6]. Tumor aerobic glycolysis can contribute to malignant transformation and tumor progression [7]. For lymphoma, the maximum standardized uptake value of invasive lymphoma is higher than that of indolent lymphoma, indicating that the invasive activity of lymphoma depends on glucose uptake [6]. However, there are currently no reliable glycolysis biomarkers for predicting DLBCL prognosis.

The tumor microenvironment (TME) constitutes a microecosystem crucial for tumor survival, encompassing tumor cells, stromal cells, and associated immune cells such as tumor-associated macrophages (TAMs), fibroblasts, T cells, and dendritic cells, along with their products including cytokines and chemokines [8]. Tumor metabolic heterogeneity can alter the TME, promoting immune evasion and cancer progression [9, 10]. Increased tumor glycolysis generates a highly acidic microenvironment, influencing the composition of infiltrating immune cells [11]. Glycolytic TME can promote metabolic reprogramming of TAMs, glycolysis-produced lactate polarizes TAMs towards an immunosuppressive M2 phenotype [12, 13], leading to elevated glycolytic metabolism [14], increased programmed death ligand−1 (PD-L1) expression in TAMs [15–17], and the formation of an immunosuppressive TME [18]. High glycolytic metabolism in TAMs can further promote tumor cell glycolytic metabolism and PD-L1 expression [19, 20]. Moreover, glycolysis characteristics correlate inversely with CD8^+^ T cell in solid tumor types and adversely affects memory T cell phenotypes [21]. Cascone et al. demonstrated that increased tumor glycolysis inhibit anti-tumor immunity by impairing T cell cytotoxic function and trafficking to the TME [10]. While several studies have reported on the role of TAMs in DLBCL [22–24], studies focusing on the reciprocal regulation between tumor cells and TAMs under conditions of high glycolytic metabolism in the TME are lacking.

Single-cell sequencing (scRNA-seq) technology enables the detection of tumor cell heterogeneity at a single-cell resolution, identification of rare cells, delineation of cell subclusters, tracking of cell lineages, localization of mutated genes, and discovery of new biomarkers [25]. This approach offers a novel perspective for studying tumor metastases. Additionally, spatial transcriptomics (ST) complements the characterization of cellular component in the spatial environment of single-cell omics, offering a high-throughput approach to explore tumor heterogeneity in spatial context [26]. We utilized scRNA-seq, bulk RNA-seq, ST, immunohistochemistry, and multiple immunofluorescence data from DLBCL tumor samples obtained from Gene Expression Omnibus (GEO) databases and the Cancer Hospital, Chinese Academy of Medical Sciences (CHCAMS) to investigate the role of high glycolysis metabolism in tumor cells and TAMs in DLBCL prognosis and immune microenvironment remodeling.

## Methods

All the materials and tools in this study were listed in the Table. 1

**Table 1:** Reagents and tools table

Reagent/Resource	Reference or Source	Catalog Number
**Single-cell RNA Sequencing**		
Single-cell RNA sequencing samples	GSE182434	Ref.[27]
CellMarker	http://biocc.hrbmu.edu.cn/CellMarker/	N/A
Panglao DB	https://panglaodb.se/	N/A
Harmony (v 0.1.1) package	https://rdocumentation.org/packages/harmony/versions/0.1.1	Ref.[31]
Seurat (v 5.0.1) package	https://github.com/satijalab/seurat	Ref.[33]
InferCNV (v 1.14.2) package	https://github.com/broadinstitute/inferCNV/wiki	Ref.[34]
irGSEA (v 2.1.5) package	https://github.com/chuiqin/irGSEA	N/A
Dorothea (v 1.7.3) package	https://github.com/saezlab/dorothea	Ref.[35]
scMetabolism (v 0.2.1) package	https://github.com/wu-yc/scMetabolism	Ref.[36]
HdWGCNA (v 0.2.23) package	https://smorabit.github.io/hdWGCNA/	Ref.[37]
Monocle3 (v 1.3.1) package	http://cole-trapnell-lab.github.io/monocle3/	Ref.[38]
Cellchat (v 1.6.1) package	https://github.com/sqjin/CellChat	Ref.[39]
MSigDB	http://www.gsea-msigdb.org/gsea/index.jsp	N/A
ClusterGVis (v 0.1.0) package	https://github.com/junjunlab/ClusterGVis	N/A
**Bulk-RNA Sequencing**		
Gene Expression Omnibus (GEO)	http://www.ncbi.nlm.nih.gov/geo	N/A
RNA sequencing samples	GSE10846	Ref.[28]
RNA sequencing samples	GSE181063	Ref.[29]
Survival (v 3.5-7) package	https://cran.r-project.org/web/packages/survival/index.html	N/A
Ggrisk (v 1.3) package	https://github.com/yikeshu0611/ggrisk	N/A
Survminer (v 0.4.9) package	https://github.com/kassambara/survminer/	N/A
TimeROC (v 0.4) package	https://cran.r-project.org/web/packages/timeROC/	Ref.[40]
Maxstat (v 0.7–25) package	https://cran.r-project.org/web/packages/maxstat/	Ref.[41]
ESTIMATE (v 1.0.13) package	https://r-forge.r-project.org/R/?group_id=2237	Ref.[42]
**Spatial Transcriptomics**		
Formalin-fixed paraffin-embedded samples	CHCAMS	N/A
Hematoxylin	S330930-2	Dako
Eosin	HT110216	Sigma-Aldrich
Glycerol	15,514,011	Thermofisher
HCl	H1758	Sigma-Aldrich
Visium Spatial Gene Expression for FFPE reagent kit	1,000,338 (human transcriptome)	10×Genomics
GSVA (v 1.46.0) package	https://github.com/rcastelo/GSVA	Ref.[43]
CARD (v 1.1) package	https://github.com/YMa-lab/CARD	Ref.[46, 47]
SPATA2 (v 2.0.4) package	https://github.com/theMILOlab/SPATA2	Ref.[50]
SPATA (v 0.1.0) package	https://github.com/theMILOlab/SPATA	Ref.[50]
**Immunohistochemistry**		
Formalin-fixed paraffin-embedded samples	CHCAMS	N/A
Rabbit anti-human STMN1 IgG antibody	ab52630	Abcam
Rabbit anti-human ENO1 IgG antibody	ab227978	Abcam
Rabbit anti-human PPIA IgG antibody	ab126738	Abcam
Rabbit anti-human PKM IgG antibody	ab137791	Abcam
Rabbit anti-human CDK1 IgG antibody	ab133327	Abcam
HRP-labeled goat anti-rabbit IgG secondary antibody	GB23303	Servicebio
hematoxylin	G1004	Servicebio
Microscope	Nikon	E100
CaseViewer 2.4	3DHISTECH	Hungary
**Multiple Immunofluorescence**		
Formalin-fixed paraffin-embedded samples	CHCAMS	N/A
Rabbit anti-human CD8 IgG antibody	ab237709	Abcam
Rabbit anti-human CD68 IgG antibody	ab303565	Abcam
Rabbit anti-human CXCL10 IgG antibody	ab306587	Abcam
Rabbit anti-human PD-L1 IgG antibody	13,684 S	Cell Signaling Technology
Rabbit anti-human TGFβ1 IgG antibody	ab215715	Abcam
HRP-labeled goat anti-rabbit IgG secondary antibody	GB23303	Servicebio
Automated immunohistochemistry stainer	Leica Bond RX	N/A
Automated digital pathology scanning system	Vectra Polaris	N/A
QuPath	https://qupath.github.io	N/A
**Software**		
IBM SPSS Statistics 24	https://www.ibm.com/support/pages/	N/A
R(4.2.1)	https://www.R-project.org/	N/A
Metascape	https://metascape.org/gp/index.html	N/A
Sanger plot website	http://www.sangerbox.com	N/A
GEPIA2	http://gepia2.cancer-pku.cn/#index	N/A
Novaseq 6000	https://www.illumina.com.cn/systems/sequencing-platforms/novaseq.html	Illumina

### DLBCL samples collection

For scRNA-seq, data from GSE182434, which encompassed four tumor samples from DLBCL and one tonsil sample from a patient with tonsillitis, were retrieved. Clinical data and metadata were obtained from the original study [27].

For bulk RNA-seq, data from two GEO datasets (http://www.ncbi.nlm.nih.gov/geo): GSE10846 (*n* = 164) [28] and GSE181063 (*n* = 802) [29] were extracted. Additionally, datasets from the Cancer Genome Atlas (TCGA) and Genotype-Tissue Expression (GTEx) databases were included for analysis. The two RNA-seq datasets analyzed were collected from tissue samples before R-CHOP treatment, with accompanying survival data. TCGA and GTEx datasets comprised 47 DLBCL patients and 491 healthy controls.

For ST, immunohistochemistry (IHC) and multiple immunofluorescence (mIF), formalin-fixed paraffin-embedded (FFPE) samples were retrospectively collected from DLBCL patients before first-line R-CHOP treatment, between 2010 and 2023 at the Cancer Hospital, Chinese Academy of Medical Sciences. Samples collected between 2019 and 2023 were utilized for ST, while those spanning from 2010 to 2020 were employed for IHC and mIF experiments. All samples were stored at room temperature. FFPE samples used for ST were confirmed by two pathologists (Dr. Tongji Xie and Dr. Lin Li) through HE staining that malignant B cells constituted at least 95% of the total B cell population.

Inclusion criteria comprised DLBCL patients with available samples before first-line R-CHOP chemotherapy, having received at least two cycles of R-CHOP with complete clinical data. Exclusion criteria included DLBCL patients with secondary primary cancers, primary central nervous system DLBCL, or DLBCL converted from indolent lymphoma. The efficacy of R-CHOP was assessed using the 2014 Lugano criteria. Based on the 24-month event-free survival (EFS24), considered a robust endpoint for disease-related outcomes in DLBCL treated with immunochemotherapy [30], DLBCL patients were categorized into relapsed (R) and non-relapsed (NR) groups.

**Fig. 1:**
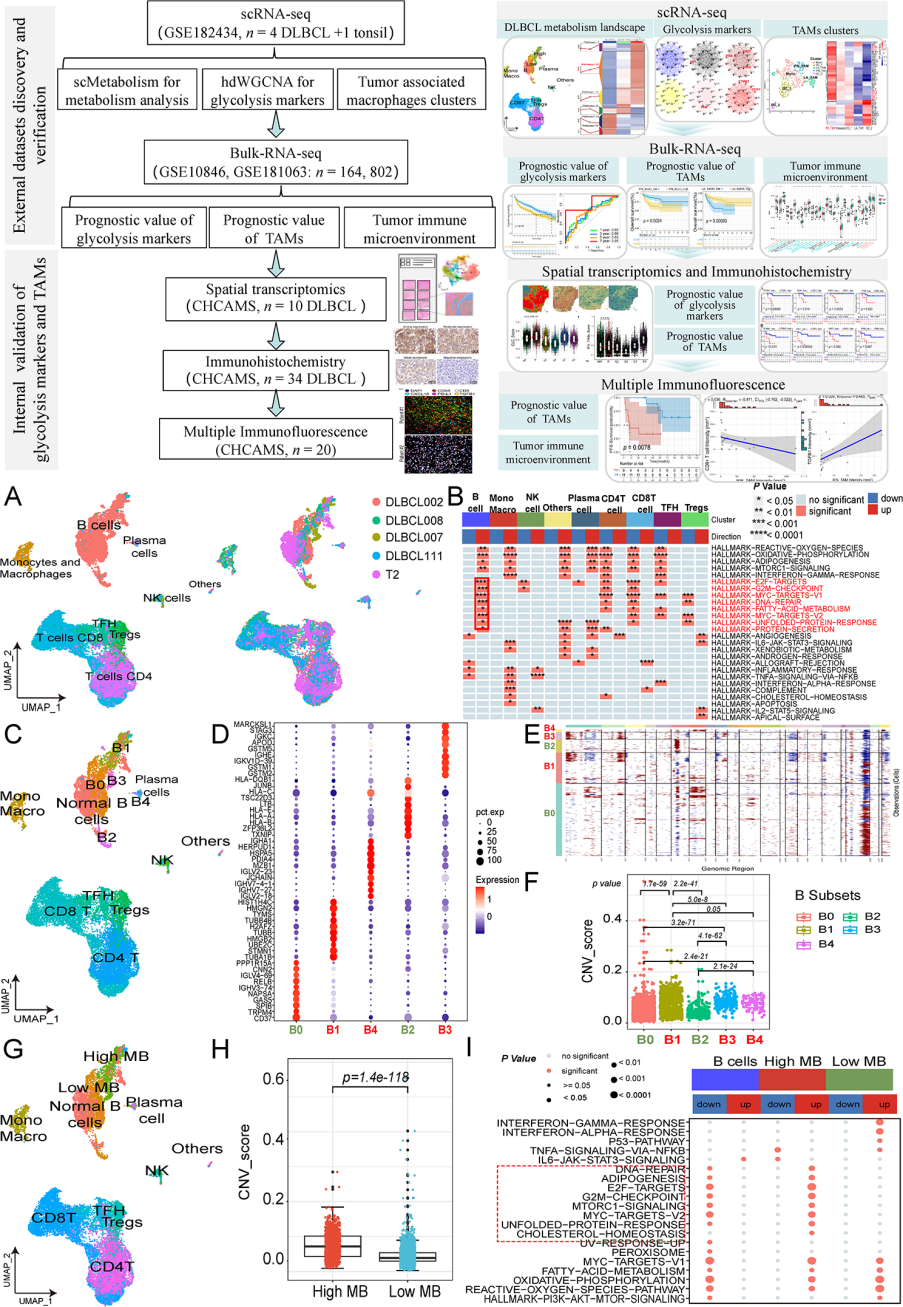
Flow chart of this study and identification of malignant B cell subgroups and CNV score comparison of scRNA-seq in GSE182434. **A** UMAP plot of cell types and samples distribution. **B.** Hallmark and pathways of different cell types determined by GSEA. **C.** UMAP plot of PCA clustering result of B malignant cells and other cell types grouping. **D.** Dot plot for expression levels of cell markers across B malignant subclusters (B0-B4). **E.** Chromosomal landscape of inferred CNVs among B malignant subclusters. **F.** Comparison of inferred CNV scores across B malignant subclusters. **G.** UMAP plot of cell types including high and low malignant B cells. **H.** Comparison of inferred CNV scores between high and low malignant B cell types. **I.** Hallmark and pathways of high and low malignant B cell types determined by GSEA. (*Abbreviation*: CNVs: copy number variations; scRNA-seq: single-cell RNA-sequencing; UMAP: uniform manifold approximation and projection; GSEA: gene set enrichment analysis; PCA: principal component analysis; DLBCL: diffuse large B-cell lymphoma; MB: malignant B cells)

In total, 64 DLBCL patients were collected across three cohorts, the ST (*n* = 10), IHC (*n* = 34), and mIF (*n* = 20) cohorts. Six out of ten patients in the ST cohort and all patients in the IHC and mIF cohorts were followed-up for over two years. Detailed patient characteristics are provided in Table S1, and the study’s flowchart is depicted in Fig. 1. This study has been approved by the Ethics Committee of the National Cancer Center/National Clinical Research Center for Cancer/Cancer Hospital, Chinese Academy of Medical Sciences & Peking Union Medical College (No. 23/262–4004). All experiments were executed according to the Declaration of Helsinki.

### Single-cell RNA sequencing data analysis

#### Quality control, multi sample integration and batch effect correction

Scrutiny was implemented on cell quality, involving filtering based on the presence of detected genes (minimum: 300, maximum: 6000), mitochondrial gene percentage (0–15%), hemoglobin gene percentage (0–0.1%), and ribosomal gene percentage (minimum: 1–100%). Additionally, any genes expressed in fewer than three cells were excluded. Then the “harmony” (v 0.1.1) package [31] facilitated the integration of expression data across various patients. Initially, expression matrices from different patients underwent normalization, scaling, and identification of variable features using the regularized negative binomial regression (“SCTransform”) [32] function of “Seurat” (v 5.0.1) package [33]. Subsequently, principal components analysis (PCA) was employed to reduce the data to a lower-dimensional space defined by the first 20 principal components (PCs). Following this, utilizing the patient ID as the batch factor, the “RunHarmony” function corrected batch effects in the low-dimensional PC representation.

#### Chromosomal copy number variations and gene set functional enrichment

Evaluation of chromosomal copy-number variations (CNVs) was conducted using the “inferCNV” (v 1.14.2) package [34], computing CNV scores across cells within each cell type. Furthermore, irGSEA (v 2.1.5) was employed for rank-based gene set enrichment analysis (GSEA).

#### Clustering and dimensionality reduction

Following data preprocessing and integration, distinct cell subclusters of tumor B cells and macrophages were individually isolated. Employing the “FindClusters” function with a resolution of 0.15 for B cells and 0.3 for macrophages and monocytes, the data were segmented. The “RunUMAP” function facilitated the visualization of a two-dimensional representation of the initial 30 PCs through uniform manifold approximation and projection (UMAP). Marker genes for each cell type were identified using the “FindAllMarkers” function, selecting those detected in a minimum of 25% of cells within the cluster, displaying a *p*-value < 0.05 in the Wilcoxon test, and demonstrating a differential expression threshold of 0.25 log fold change (log *FC*). Visualization functions like DotPlot, VlnPlot, and DoHeatmap were utilized to illustrate the differentially expressed genes.

#### Transcription factor activity

The transcription factor (TF) activity was inferred using DoRothEA (v 1.7.3) package [35]. Specifically selecting high-confidence TFs (“A”, “B”, and “C”) based on “dorothea_hs” regulons provided by the “DoRothEA” package, Viper scores were calculated, scaled, and integrated into the Seurat object as the “Dorothea” attribute. To enable a comparison of TF score activities, mean and standard deviation were computed for scaled viper scores within each cell type. TFs were ranked based on the variance of their respective viper scores. The top 20 TFs exhibiting highly variable scores in each cell type were chosen for visualization.

#### Cell metabolic activity and hdwgcna analysis

The “scMetabolism” [36] (v 0.2.1) package, designed for quantifying single-cell metabolism, systematically evaluated and scored clusters within individual metabolic pathways (*n* = 79) from conventional single-cell matrix files using a vision algorithm. High-dimensional weighted gene co-expression network analysis (hdWGCNA) (v 0.2.23) package [37] was employed to delineate the key molecular characteristics of highly malignant B cells. Utilizing a soft threshold of 5, a scale-free network was constructed for optimal connectivity, resulting in the identification of 10 gene modules.

#### Pseudotime analysis and cell-cell communication

Pseudotime analysis was executed using the R package “Monocle3” (v 1.3.1) [38]. Dimensionality reduction via the UMAP method facilitated visualization, and the “plot_cells” function aided in visualization. Additionally, the “graph_test” function was utilized to identify differentially expressed genes along the pseudotime trajectory.

The “Cellchat” (v 1.6.1) package [39] was utilized to explore cell-cell communication. Specific categories like “Secreted Signaling”, “ECM-Receptor”, and “Cell-Cell Contact” within the Cellchat database underwent examination, applying a minimum cell count criterion of 3. Markers for the “hallmark_glycolysis” pathway were obtained from the Molecular Signature Database (MSigDB). Visualization of dynamic trends within metabolic pathways was achieved using the “ClusterGVis” (v 0.1.0) package, employing the k-means clustering method. Differences between groups were analyzed using Mann-Whitney U tests.

### Bulk RNA sequencing in GEO datasets

Datasets GSE10846 (platform GPL570, *n* = 164) and GSE181063 (platform GPL14951-11332, *n* = 802) were annotated for comprehensive analysis. Raw data underwent rigorous quality control using the “Affy” package within R software. This involved the computation of average values for multiple probes corresponding to a single gene. For comparative analysis of messenger RNA (mRNA) expression of seven prognostic markers in TCGA and GTEx, Gene Expression Profiling Interactive Analysis 2 platform (GEPIA2) (http://gepia2.cancer-pku.cn/#index) was employed. Univariate and multivariate Cox analyses were conducted for overall survival (OS) using “survival” (v 3.5-7) package. Visualization of the results included scatter plots, risk score heatmaps, and time-dependent receiver operating characteristic (ROC) curves generated through the use of the “ggrisk” (v 1.3), “survminer” (v 0.4.9), and “timeROC” [40] (v 0.4) packages, respectively. Optimal cutoff values for distinguishing high- and low-expression groups were determined utilizing the “Maxstat” (v 0.7–25) package [41] in R software. ESTIMATE package [42] (v 1.0.13) was used to calculate the stromal and immune content (stromal score, immune score, and ESTIMATE score) in all patients with DLBCL. Furthermore, leveraging marker genes specific to each cell type, single-sample Gene Set Enrichment Analysis (ssGSEA) scores were computed across cell types within GEO datasets using the “GSVA” (v 1.46.0) package [43].

### Spatial transcriptomics analysis

#### Experiment procedure

This study utilized the Visium technology platform by 10x Genomics, with all experimental materials sourced from this platform (https://www.10xgenomics.com/products/spatial-gene-expression). Detailed procedures are presented in Table S2.

#### Data preprocessing

Each sequenced ST library was processed and aligned to the GRCh38 human reference genome using Space Ranger software (version 2.0.0) developed by 10x Genomics. Subsequently, unique molecular identifier (UMI) counts were aggregated for each specific spot. To distinguish tissue overlaying spots from the background, identification of tissue overlaying spots was performed based on image analysis. Only barcodes linked to these tissue overlaying spots were preserved, resulting in the generation of filtered UMI count matrices. Moreover, we manually excluded spots not covered by tissue yet undetected by Space Ranger, further refining the UMI count matrices.

#### Samples integration

Individual data were imported into R for samples integration, processing the filtered UMI count matrix using the R package Seurat (version 4.1.0). The “SCTransform” method was used for UMI count matrix normalization. After merging ten slices for joint analysis, PCA was employed to project data into a lower-dimensional space encompassing the first 20 PCs. To rectify batch effects, the “RunHarmony” function was applied, utilizing patient ID as the batch factor to mitigate the influence of batch effects [44, 45].

#### CARD deconvolution and celltype annotation

The “CARD” (v 1.1) package [46, 47] was employed to deconvolute ST data based on four DLBCL scRNA-seq count datasets within GSE182434. A “CARD” object was generated utilizing the “CreateCARDObject” function, followed by application of the “CARD_deconvolution” function with default parameters to compute the results.

Following preprocessing steps such as SCTransform, PCA, and data integration via harmony, ST spots were stratified into discrete clusters using the “FindClusters” function with a resolution parameter set at 0.5. UMAP visualization of the first 30 PCs using “RunUMAP” provided a two-dimensional representation of the identified clusters. Marker gene identification was performed via “FindAllMarkers” following “PrepSCTFindMarkers”, considering genes detected in at least 25% of cells within the cluster, exhibiting a Wilcoxon test *p*-value < 0.05, and demonstrating a differential expression threshold of 0.25 log*FC*. Marker genes were cross-referenced with known cell types using the CellMarker (http://biocc.hrbmu.edu.cn/CellMarker/) [48] and Panglao DB (https://panglaodb.se/) [49] databases.

#### InferCNVs and SPATA2

Inferred CNVs analysis followed the process similar to scRNA-seq. The “SPATA2” (v 2.0.4) package [50] was employed to validate the precision of cell type annotations at the inferCNV level. Functions such as “initiateSpataObject_CountMtr”, “runCnvAnalysis”, “plotCnvLineplot”, and “SPATA2::plotSurface” were utilized to compute chromosomal copy-number variations and generate visual representations illustrating CNV variations.

#### Intratumoral heterogeneity score

Following the methodology outlined in reference [51], an examination of intratumoral heterogeneity (ITH) was performed. ITH assessment involved evaluating individual cells within the tumor using PCA coordinates as distinctive features. The process computed the distance from each feature to the centroid, establishing an average distance of all cells to the centroid, characterizing intratumoral cellular heterogeneity within the sample.

#### Gene set enrichment analysis and SPATA

The methodology for conducting irGSEA analysis was consistent with the scRNA-seq process. To visualize hallmark pathways, the “SPATA” (v 0.1.0) package was conducted. The “initiateSpataObject_10X” function was employed to generate a “spata_obj” followed by the utilization of the “SPATA::plotSurfaceComparison” function for visualization.

#### Cell metabolic activity and celltype score

The methodology employed for Metabolism analysis was the same as scRNA-seq analysis. Calculations for glycolysis (GLC) risk score and activated CD8^+^ T score utilized the “AddModuleScore” function within the “Seurat” package.

### Immunohistochemistry validation

All patient samples underwent hematoxylin-eosin (HE) staining and were meticulously reviewed and confirmed by two experienced pathologists to identify cancer lesions. IHC was performed on FFPE samples after dewaxing and heat-induced antigenic repair. Samples were washed and incubated with a 3% hydrogen peroxide solution to quench endogenous peroxidase activity. FFPE samples were incubated with primary rabbit anti-human IgG antibodies specific to stathmin 1 (STMN1, ab52630, Abcam), enolase 1 (ENO1, ab227978, Abcam), peptidylprolyl isomerase A (PPIA, ab126738, Abcam), pyruvate kinase M (PKM, ab137791, Abcam), and cyclin dependent kinase 1 (CDK1, ab133327, Abcam) at dilutions of 1:1000, 1:1000, 1:50, 1:500, and 1:250. respectively, following blocking with rabbit serum. After washing, FFPE samples were incubated with a 1:200 dilution of HRP-labeled goat anti-rabbit IgG secondary antibody (GB23303, Servicebio) for 50 min at room temperature. Diaminobenzidine color development was used to visualize the results, and the nuclei were re-stained using hematoxylin (G1004, Servicebio). Results were then interpreted under a white light microscope (E100, Nikon) and proteins were quantified using CaseViewer 2.4 (3DHISTECH, Hungary) software.

Protein expression were quantified by *H* Score. *H* Score was calculated based on the intensity of the stain and the percentage of positive tumor cells, with scores ranging from 0 to 300. Stain intensity was classified as negative (0 scores), weak (1 score), moderate (2 scores), and strong (3 scores) stain, and the percentage of positive cells was scored from 0 to 100. *H* Score was calculated as the product of intensity and percentage. *H* Scores below 60 were determined as low expression, while *H* Scores greater than or equal to 60 were considered high expression.

### Multiple immunofluorescence validation

FFPE tissue sections of 4–5 μm thickness were prepared, followed by dewaxing and rehydration. Antigen retrieval was performed, and endogenous peroxidase activity was blocked with antibody blocking solution. All immunohistochemical procedures were performed using the fully automated immunohistochemistry stainer, Leica Bond RX. Sequential immunostaining was performed for each target antigen, including primary antibodies against rabbit anti-human IgG antibody CD8 (ab237709, dilution 1:500, Abcam), CD68 (ab303565, dilution 1:500, Abcam), CXCL10 (ab306587, dilution 1:2000, Abcam), PD-L1 (13,684 S, dilution 1:800, Cell Signaling Technology), and TGFβ1 (ab215715, dilution 1:500, Abcam), followed by incubation with secondary antibodies: HRP-labeled goat anti-rabbit IgG secondary antibody (GB23303, dilution 1:500, Servicebio) for CD8, CD68, CXCL10, PD-L1, and TGFβ1. Tyramide signal amplification (TSA) wase employed, followed by microwave treatment to remove the TSA-antibody complex, enabling subsequent rounds of antibody labeling. iF570-Tyramide was for CD68, iF480-Tyramide was for CD8, iF780-Tyramide was for CXCL10, iF520-Tyramide was for PD-L1, and iF690-Tyramide was for TGFβ1. Following immunostaining, cell nuclei were counterstained with 4’,6-diamidino-2-phenylindole (DAPI), and slides were coverslipped for scanning. The whole-slide scanning was conducted by the automated digital pathology scanning system, Vectra Polaris. QuPath (https://qupath.github.io*)* software was employed for quantification of the number and percentage of positive cells.

## Results

### Study design

The overall study design, as illustrated in Fig. 1, consisted of two-phase: external datasets discovery and verification in scRNA-seq (*n* = 5) and GEO datasets (*n* = 966) cohorts and internal validation of glycolysis markers and TAMs in ST (*n* = 10), IHC (*n* = 34), and mIF (*n* = 20) cohorts. First, using the scMetabolism algorithm and clustering of B cells and TAMs at the single-cell level in DLBCL, we found that high glycolytic metabolism was associated with the malignancy of tumor B cells and identified a subset of TAMs with high glycolytic activity. Through hdWGCNA and gene set scoring, we identified key genes involved in glycolytic metabolism. Validation at the transcriptomic level indicated that a high glycolytic metabolism score and higher levels of glycolytic TAMs are associated with poorer prognosis and a lower infiltration of CD8^+^ T cells. Subsequent validation using ST, IHC, and mIF cohorts from CHCAMS further confirmed that a high glycolytic metabolism score and higher levels of glycolytic TAMs correlate with poorer prognosis and reduced CD8^+^ T cell infiltration. Detailed clinical characteristics were shown in Table S1.

### Identification of a highly malignant B cells in DLBCL by scRNA-seq

Prior to harmony integration, while patient samples exhibited distinct separation in the UMAP plot, there was no significant segregation among cell types (Fig. S1A, B). However, following harmony integration, distinct cell types were clearly segregated, accompanied by confluent patient sample distributions (Fig. 1A). InferCNVs analysis unveiled that B cells exhibited the highest CNV scores among various cell types (*p* < 0.0001) (Fig. S1C, D). Subsequent comparison between malignant tumor and benign B cells, as classified in the original study [27], revealed significantly higher CNV scores in malignant B cells compared to benign B cells (*p* < 0.0001) (Fig. S1E, F). Functional enrichment analysis underscored distinct features of B cells, particularly marked by enrichment in E2F-targets, G2M-checkpoint, MYC-targets, DNA-repair, and fatty-acid metabolism hallmark pathways, while exhibiting reduced activity in angiogenesis, allograft-rejection, inflammatory-response, and TNFA-signaling-via-NFKB pathways (*p* < 0.05) (Fig. 1B).

PCA clustering revealed five distinct subtypes of malignant B cells (Fig. 1C), as depicted in the dot plot (Fig. 1D). Detailed differential gene expression among these B cell subtypes were presented in Fig. S2A. Previous studies have reported that CNV levels are positively correlated with the malignancy of T-cell [52] and B-cell [53] lymphomas. So InferCNVs analysis was performed across B cell subtypes. InferCNVs analysis further illustrated that B1, B3, and B4 subtypes exhibited higher CNV scores compared to B0 and B2 subtypes (*p* < 0.05) (Fig. 1E, F). Consequently, grouping B1, B3, and B4 as highly malignant B cell types, and B0 and B2 as low malignant B cell types was established (Fig. 1G). The highly malignant B cells demonstrated significantly higher CNV scores compared to the low malignant B cells (*p* = 1.4e-118) (Fig. 1H) and exhibited enriched activity in E2F-targets, G2M-checkpoint, MYC-targets, and DNA-repair hallmark pathways (*p* < 0.05) (Fig. 1I). Detailed clinical characteristics and the distribution of 15 cell types among four DLBCL patients and one tonsillitis patient are presented in Table S3.

### Highly malignant B cells reveals elevated glycolysis metabolic activity

**Fig. 2:**
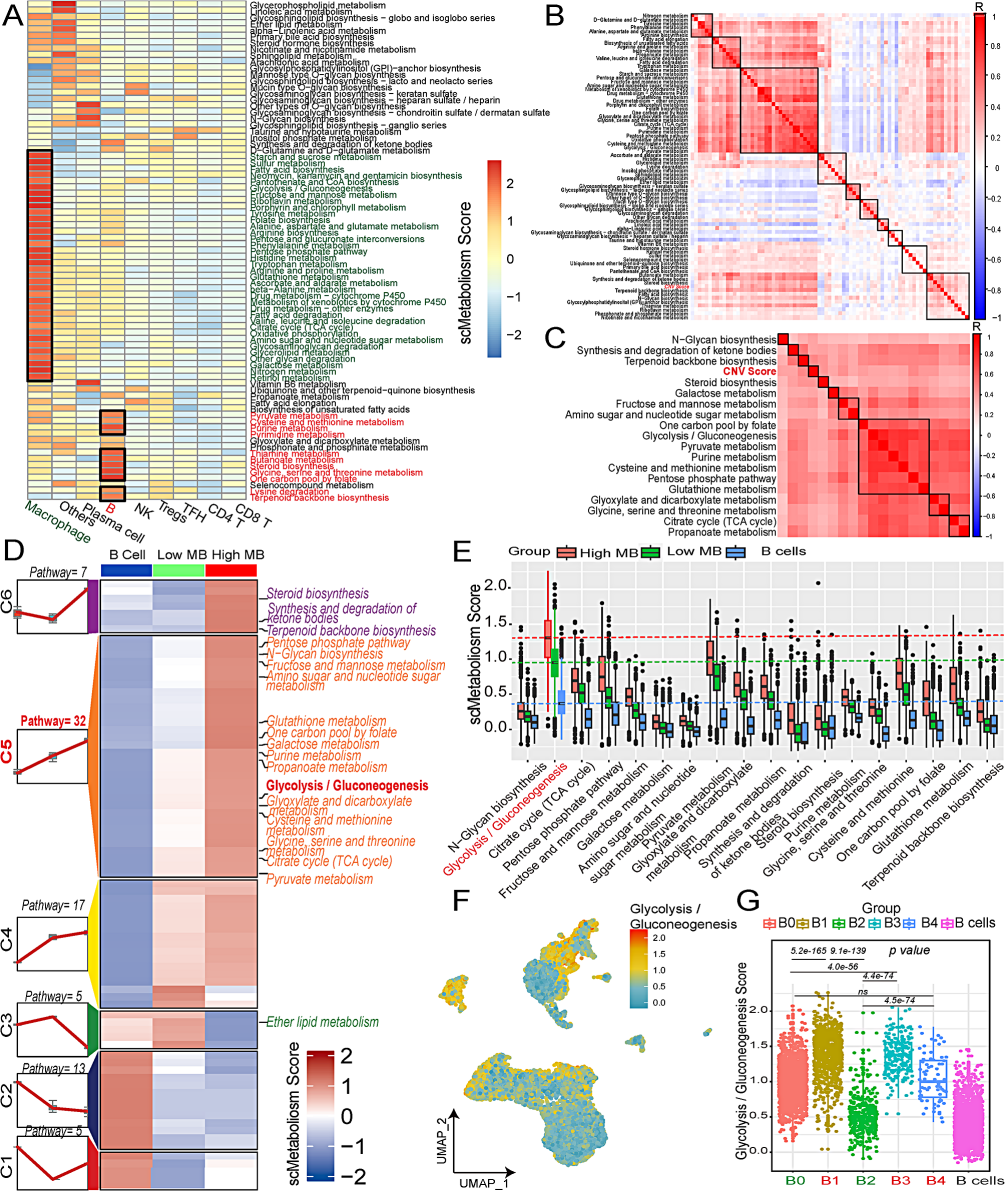
Metabolism altas of samples in single-cell RNA-sequencing. **A** Metabolism enrichment of different cell types by heatmap. **B.** Correlation between inferred CNV scores and metabolism pathway scores. **C.** 18 metabolism pathway scores correlation with inferred CNV scores (*r* > 0.3 and *p* < 0.05). **D.** Clusters of metabolism pathway (*n* = 79) from benign B cells, low malignant B cells, to high malignant B cells. **E.** Comparison of 18 metabolism pathway scores among benign B cells, low malignant B cells, and high malignant B cells. **F.** UMAP plot of glycolysis / gluconeogenesis pathway score. **G.** Barplot of glycolysis / gluconeogenesis pathway scores in benign B cells and B0-B4 subgroups (*Abbreviation*: CNV: copy number variation; MB: malignant B cells; UMAP: uniform manifold approximation and projection. Mann-Whitney test was performed between groups.)

The analysis of metabolic activity among various cell types revealed that macrophages and monocytes exhibited predominant metabolic activity across a majority (36/79) of metabolic pathways (Fig. 2A). Specifically, B cells demonstrated elevated activity in purine metabolism, thiamine metabolism, butanoate metabolism, steroid biosynthesis, glycine, serine and threonine metabolisms, and one-carbon pool by folate(Fig. 2A). Exploring the correlation between metabolic pathways and malignant degree unveiled 18 metabolic pathways with positive correlations (*p* < 0.05 and *r* > 0.3) with CNV scores (Fig. 2B and C; Table S4). A cluster analysis categorized the 79 metabolic pathways among benign B cells, low malignant B cells, and highly malignant B cells into six distinct categories (Fig. 2D). Notably, the majority (15/18) of pathways displaying positive correlations were clustered in cluster 5, which exhibited a consistent upward trend among the three groups. The analysis of the 18 metabolic pathways indicated that glycolysis/gluconeogenesis pathway had significantly elevated activity in all three groups (*p* < 0.05) (Fig. 2E). This pathway displayed a clear increasing trend across B cells, low malignant B cells, and high malignant B cells (Fig. 2E). The UMAP plot and barplot depicting B0-B4 cells further supported this marked elevation (*p* < 0.05) (Fig. 2F, G). Additionally, the metabolic level of glycolysis/gluconeogenesis was elevated in monocytes/macrophages (Fig. 2A, F).

### Glycolysis metabolic gene selection and IFN_TAMs demonstrated cell-cell communication with highly malignant B cells and CD8^+^ T cells

**Fig. 3:**
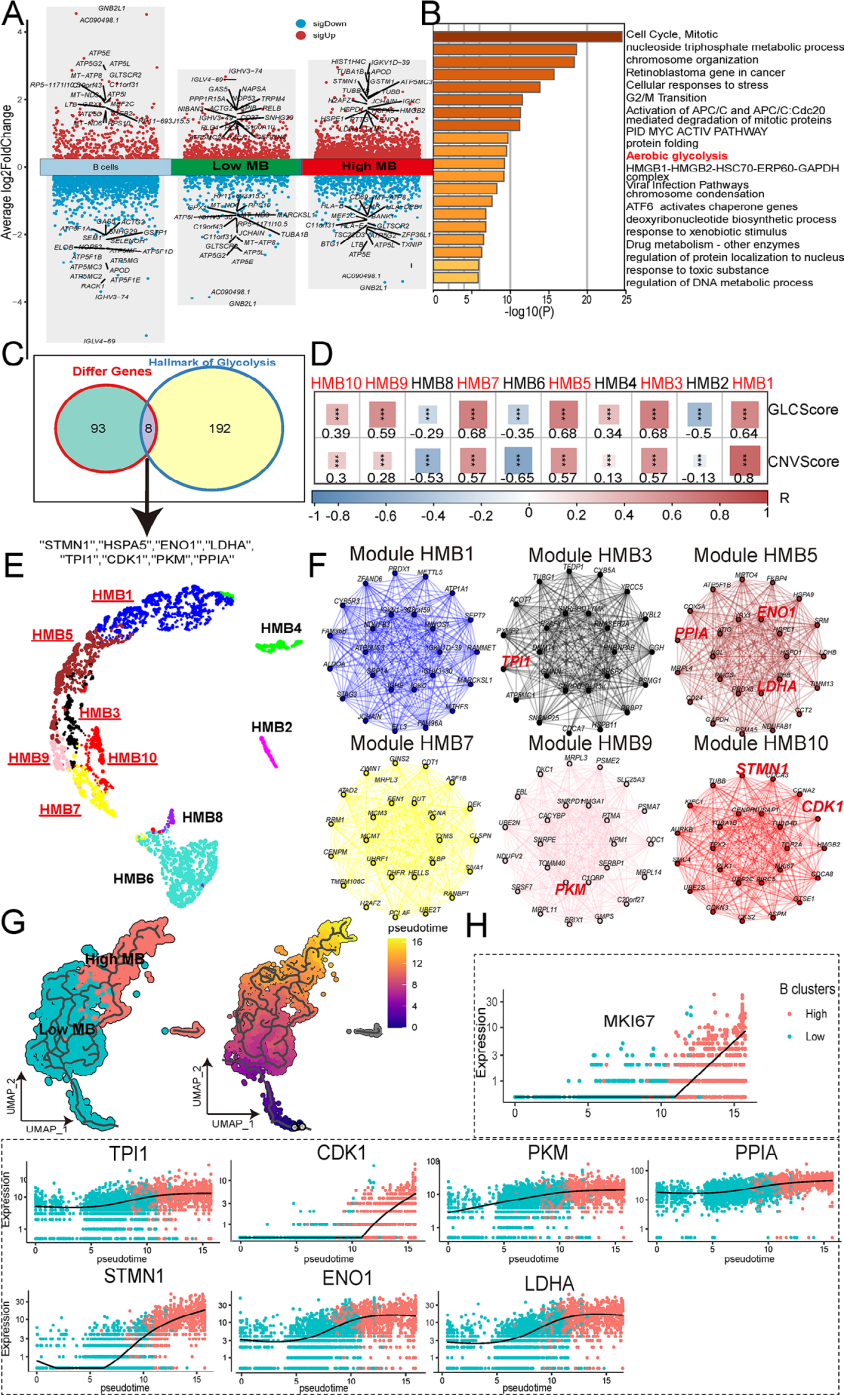
Identifcation of glycolysis / gluconeogenesis maker genes in high malignant B cells. **A** Volcano plot of differential genes among the benign B cells, low malignant B cells, and high malignant B cells. **B.** Functional analysis of highly expressed genes in high malignant B cells by Metascape. **C.** Identification of eight commonly genes overlapped across three groups and glycolysis hallmark genes. **D.** Module trait correlation showed the relationships between modules, CNV score, and Glycolysis score. **E.** Network visualization of 10 modules of high maligant B cells.(*The modules highlighted in red and underlined are modules associated with CNV score and Glycolysis score.*) **F.** The first 25 eigengenes of each module. **G.** Trajectory of different malignant B subclusters predicted by monocle. **H.** Genes expression level in single spot ordered along the pseudotime for MKI67 and seven glycolysis / gluconeogenesis gene markers (*STMN1, ENO1, LDHA, TPI1, CDK1, PKM*, and *PPIA*). (*Abbreviation*: HMB: high malignant B cells; CNV: copy number variation; UMAP: uniform manifold approximation and projection. *** *p* < 0.001.)

To identify marker genes associated with glycolysis/gluconeogenesis, differential analyses were conducted among benign B cells, low malignant B cells, and highly malignant B cells (Fig. 3A). Highly malignant B cells exhibited elevated levels of *HIST1H4C, IGKVID-39, TUBA1B, APOD, GSTM1, ATP5MC3, STMN1, TUBB, TUBB4B*, and *JCHAIN*, while showed decreased levels of *GNB2L1, AC090498.1, ATP5E, LTB, ATP5L, ATP5G2, TXNIP, ZFP36L1, TSC22D3*, and *BTG1* (Fig. 3A). And the transcription factor of low malignant B cells, and highly malignant B cells were shown in Fig. S2B, which showed high transcription factor activity in MYC, NFKB2, ATF6, MYCN, FOSL2, HSF1, HHEX, YY1, FOXA1, and GLI2 in high malignant B cells. Functional analysis showed that highly malignant B cells were enriched in the aerobic glycolysis pathway (Fig. 3B). A total of 101 genes were identified as highly expressed in high malignant B cells compared with low malignant B cells and normal B cells, meeting the criteria of average *log2FC* > 1.0 and *p* < 0.05 (Fig. 3C and Table S5). Eight genes (*STMN1*, *HSPA5*, *ENO1*, *LDHA*, *TPI1*, *CDK1*, *PKM*, and *PPIA*) were found to overlap with the hallmark_glycolysis geneset (Fig. 3C). hdWGCNA was employed to delineate the key molecular characteristics of highly malignant B cells. Utilizing a soft threshold of 5, a scale-free network was constructed for optimal connectivity, resulting in the identification of 10 gene modules (Fig. S3A-E). Modules HMB1, 3, 5, 7, 9, and 10 exhibited positive correlations (*p* < 0.05, *R* ≧ 0.28) with CNV score and glycolysis score (Fig. 3D), and co-expression networks demonstrated their cohesive association (Fig. 3E). The first 25 eigengenes of each module indicated contributions from *STMN1*, *ENO1*, *LDHA*, *TPI1*, *CDK1*, *PK*M, and *PPIA* to the HMB1, 3, 5, 7, 9, and 10 modules (Fig. 3F). Pseudotime analysis revealed a differentiation trajectory from low-grade malignant B cells and then to highly malignant B cells (Fig. 3G). Notably, all seven marker genes along with the cell proliferation gene proliferation marker protein Ki-67 (MKI67) displayed elevated expression levels (Fig. 3H). The UMAP plots further validated their higher expression in highly malignant B cells (Fig. S3E).

**Fig. 4:**
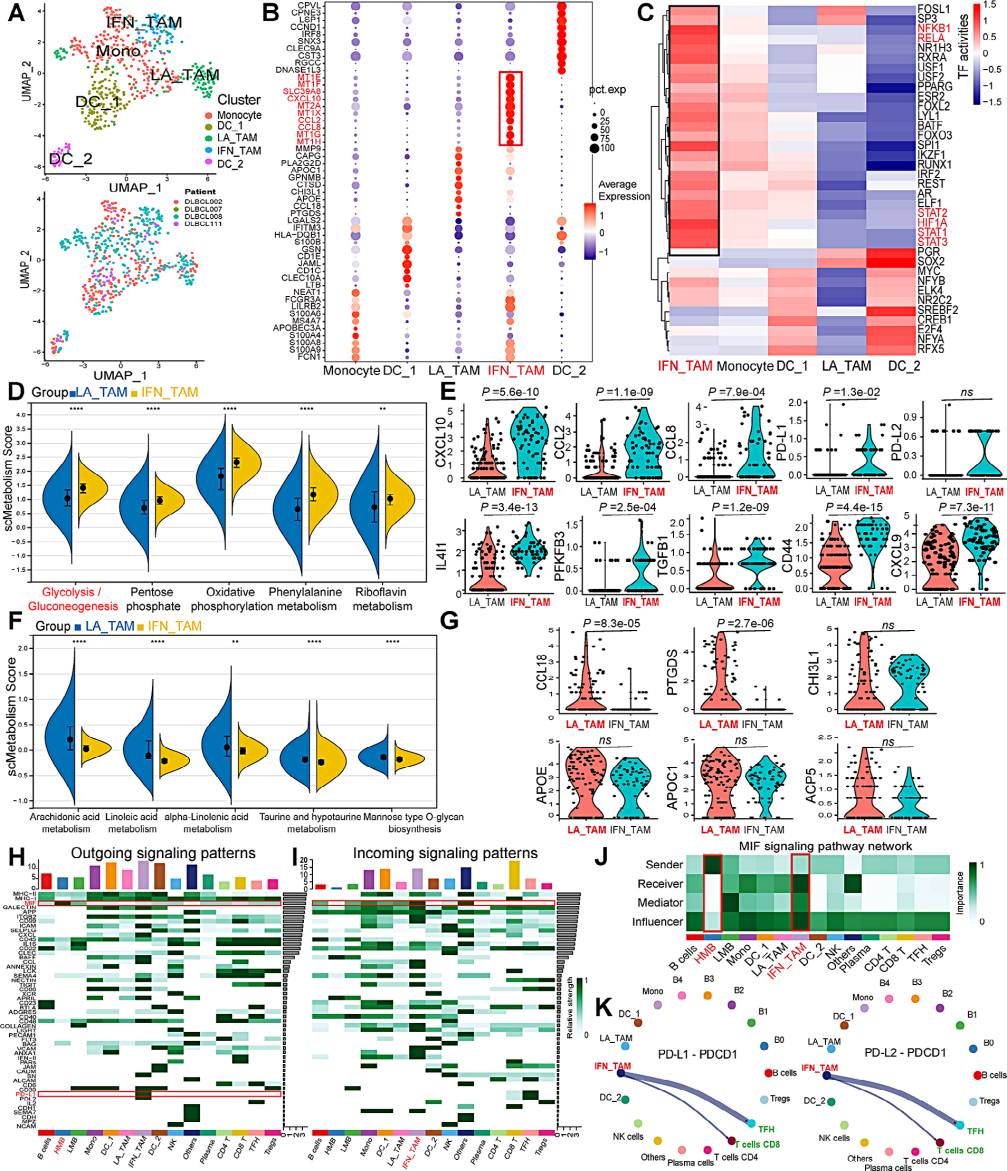
Macrophage subgroups identification and Cell-cell Communications in single-cell RNA-sequencing. **(A)** UMAP plot of PCA clustering result of macrophage and samples clustering. **(B)** Dot plot for cell marker expression levels. **(C)** Heatmap representation of top 20 highly variable transcription factor activities. **(D)** Top 5 higher metabolic pathways in IFN_TAMs compared with LA_TAMs. **(E)** Comparison of *CXCL10, CCL2, CCL8, PD-L1, PD-L2, IL4I1, PFKFB3, TGFB1* and *CD44* gene expression in LA_TAMs and IFN_TAMs. **(F)** Top 5 higher metabolic pathways in LA_TAMs compared with IFN_TAMs. **(G)** Comparison of *CCL18, PTGDS, CHI3L1, APOE, APOC1*, and *ACP5* gene expression in LA_TAMs and IFN_TAMs. **H-I.** Heatmap of cell-cell communication network for incoming and outgoing signaling analysis. **J.** Heatmap of the relative importance of cell groups in the MIF signaling network based on four network centrality degrees. **K.** Circular plot of the quantity or intensity of interactions among various cell groups in PD-L1-PDCD1 and PD-L2-PDCD1 networks (*Abbreviation*: UMAP: uniform manifold approximation and projection; PCA: principal component analysis; IFN_TAMs: interferon-primed tumor-associated macrophages; LA_TAMs: lipid-associated tumor-associated macrophages; MIF: macrophage migration inhibitory factor. Mann-Whitney test was performed between groups. * *p* < 0.05, ** *p* < 0.01, *** *p* < 0.001, **** *p* < 0.0001, ns, not significant.)

As shown in Fig. 2F, elevated glycolysis/gluconeogenesis pathways were detected in monocytes/macrophages. Given the heightened metabolic activity of macrophages and monocytes in DLBCL, macrophages and monocytes were clustered into five distinct clusters (Fig. 4A, Table S6), with representative markers illustrated in Fig. 4B. Based on previous literature classifications [54–58] of macrophages, monocytes, and dendritic cells, we categorized five cell types using specific markers as monocytes (FCN1^+^ S100A8^+^ APOBEC3A^+^), dendritic cells_1 (CLEC10A^ +^ CD1C^+^ CD1E^+^), lipid-associated tumor-associated macrophages (LA_TAMs) (APOC1^ +^ APOE^+^ ACP5^+^ CCL18^+^), interferon-primed_TAMs (IFN_TAMs) (PD-L1^+^ PD-L2^+^ CXCL10^+^), and dendritic cells 2 (CLEC9A^ +^ THBD^+^). Enhanced TF activities for RELA, NFKB1, and HIF1A (known as glycolytic promoting factors) were specifically observed in IFN_TAMs (Fig. 4C). IFN_TAMs exhibited higher glycolysis/gluconeogenesis activity than LA_TAMs (*p* < 0.0001) (Fig. 4D). *CXCL10*, *CCL2*, *CCL8*, *PD-L1*, *IL4I1*, *PFKFB3*, *TGFB1* and *CD44* genes exhibited higherlevels (*p* < 0.05) in IFN_TAMs than LA_TAMs, while the difference of *PD-L2* level was not significant (Fig. 4E). While fatty acid-related metabolic pathways, such as arachidonic acid metabolism, linoleic acid metabolism, and alpha-Linolenic acid metabolism pathways were higher in LA_TAMs than IFN_TAMs (Fig. 4F), along with higher expression (*p* < 0.05) in *CCL18* and *PTGDS* gene in LA_TAMs, while the difference of *CHI3L1*, *APOE*, *APOC1*, and *ACP5* levels were not significant (Fig. 4G). Analysis based on specific pathways and ligand receptors unveiled intricate interactions among malignant B cell subtypes and other cell types in DLBCL. A total of 54 pathways among 18 cell types were detected, with the macrophage migration inhibitory factor (MIF) pathway emerging as a prominent mode of both incoming and outgoing signaling (Fig. 4H, I) in malignant B cells. Specifically, highly malignant B cells were identified as the prominent senders, while IFN_TAMs exhibited the strongest receptivity to the MIF pathway (Fig. 4J). Notably, IFN_TAMs displayed a specific interaction pattern with CD8^+^ T cells and follicular helper T cell (TFH) via the PD-L1-PDCD1 and PD-L2-PDCD1 ligand receptors (Fig. 4K).

### Prognostic value of glycolysis markers, IFN_TAMs, and LA_TAMs

**Fig. 5:**
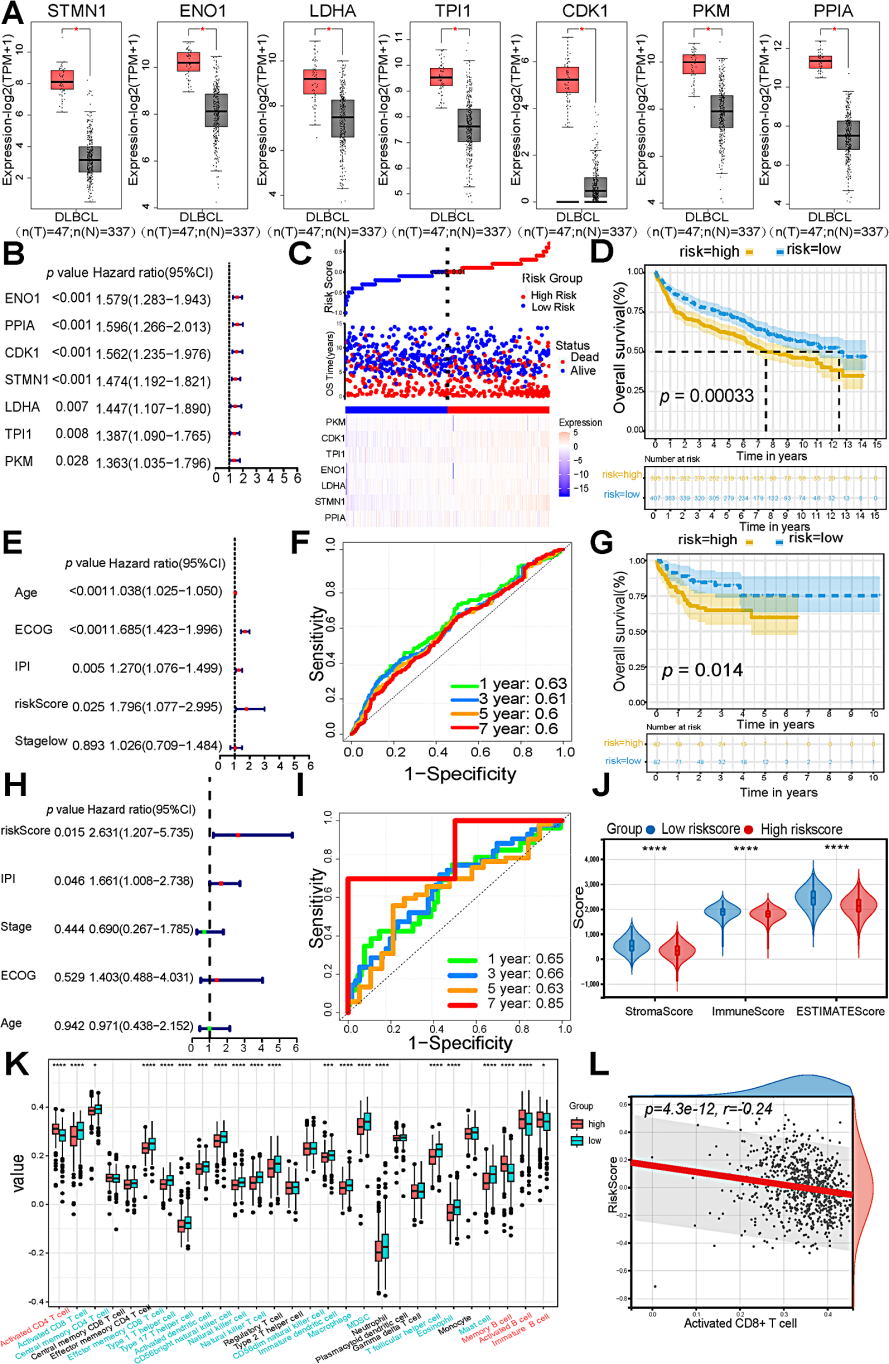
Performance of seven glycolysis / gluconeogenesis markers in predicting OS in GSE181063 (*n* = 802) and GSE10846 (*n* = 164), and relationship between risk score and immune landscape in bulk-RNA seq. **A** Comparisons of seven glycolysis / gluconeogenesis genes’ mRNA expression in DLBCL (*n* = 47) and HCs (*n* = 491). **B and E.** Univariate and Multivariate Cox analysis for OS in GSE181063. **C-D.** Scatter and heatmaps for the seven markers-based risk score and Kaplan-Meier curves of OS in GSE181063. **F and I.** Time-dependent ROC curves for OS of the seven markers-based risk score in GSE181063 and GSE10846. **G.** Kaplan-Meier curves of OS based on seven markers-based risk score in GSE10846. **H.** Multivariate Cox analysis for OS in GSE10846. **J.** Comparisons of Estimate, Stromal and Immune scores among high riskscore and low riskscore patients in GSE181063. **K.** Distribution of 28 immune cell types in high and low risk groups in GSE181063. **L.** Correlation between riskscore and activated CD8^+^ T cell in GSE181063. (*Abbreviation*: DLBCL: diffuse large B-cell lymphoma; HC: healthy controls; OS: overall survival; ROC: receiver operating characteristic; ECOG: Eastern Cooperative Oncology Group; IPI: International Prognostic Index. Mann-Whitney test was performed between groups. * *p* < 0.05, ** *p* < 0.01, *** *p* < 0.001, **** *p* < 0.0001, ns, not significant.)

**Fig. 6:**
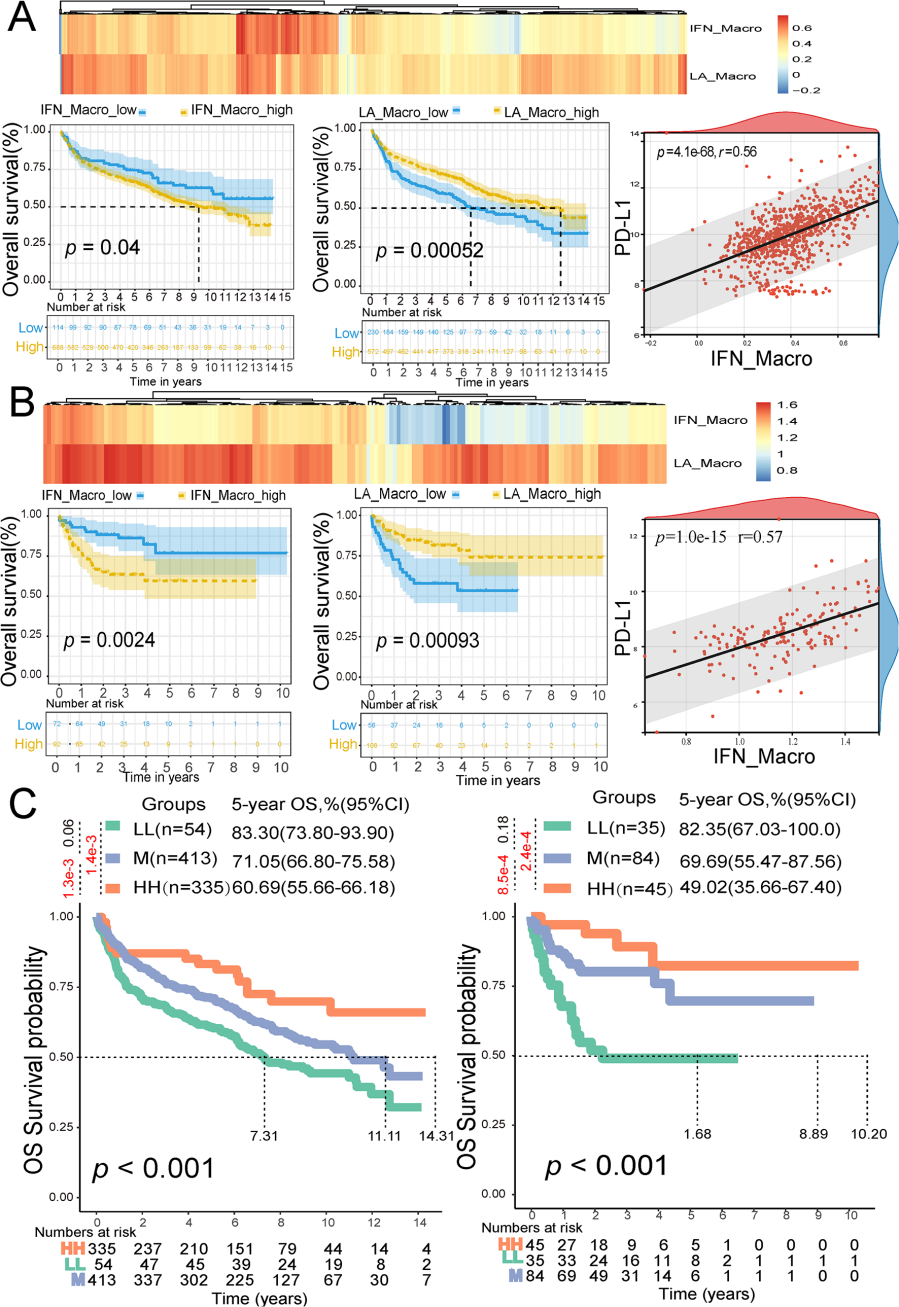
Performance of IFN_TAMs and LA_TAMs in predicting OS and association with PD-L1 in GSE181063 (*n* = 802) and GSE10846 (*n* = 164). **(A)** Kaplan-Meier analysis for OS based on IFN_TAMs and LA_TAMs (calculated by ssGSEA), and correlation between IFN_TAMs and PD-L1 in GSE181063. **(B)** Kaplan-Meier analysis for OS based on IFN_TAMs (calculated by ssGSEA) and LA_TAMs (calculated by ssGSEA), and correlation between IFN_TAMs and PD-L1 in GSE10846. **(C)** OS stratified by the glycolysis markers-based risk score combined with the IFN_TAM. (*Abbreviation*: IFN_TAMs: interferon-primed tumor-associated macrophages; LA_TAMs: lipid-associated tumor-associated macrophages; OS: overall survival; HH: IFN_TAMs high and glycolysis markers-based risk score high; M: IFN_TAMs high and glycolysis markers-based risk score low or IFN_TAMs low and glycolysis markers-based risk score high; LL: IFN_TAMs low and glycolysis markers-based risk score low.)

Expression levels of *STMN1*, *ENO1*, *LDHA*, *TPI1*, *CDK1*, *PKM*, and *PPIA* mRNAs were significantly higher in DLBCL patients (*n* = 47) compared to healthy controls (*n* = 491) based on the TCGA and GTEx datasets (*p* < 0.05) (Fig. 5A). All seven genes were associated with OS through univariate Cox regression and Kaplan-Meier curves (*p* < 0.05) in GSE181063 (Figs. 5B and S4). A risk score model based on the seven glycolysis markers was constructed. The risk score was calculated using the formula: risk score = Σ (Expression * Coefficient). The coefficients were calculated by the COX regression in the “rms” package. Based on the optimal cutoff value, samples were stratified into high- and low-risk score groups (Fig. 5C). The high-risk score group exhibited significantly worse OS (*p* < 0.05) (Fig. 5D) compared to the low-risk score group. In multivariate Cox analyses, low risk score remained an independent predictor of OS (*hazard ratio* = 0.755; *95% CI*, 0.590–0.966) (Fig. 5E). The risk score model showed consistent performance with area under the curves (AUCs) for predicting 1, 3, 5, and 7-year OS rates of 0.63, 0.61, 0.6, and 0.6, respectively (Fig. 5F). Comparable results were observed for OS in GSE10846, where the risk score remained an independent factor for OS (Fig. 5G, H). The AUCs for predicting 1, 3, 5, and 7-year OS rates were 0.65, 0.66, 0.63, and 0.85 (Fig. 5I). According to ESTIMATE algorithms, low riskscore patients performed higher (*p* < 0.05) stromal score, immune score, and ESTIMATE score(Fig. 5J). The high-risk score group exhibited an exhausted immune environment, indicated by reduced infiltration of activated CD8^+^ T cells and natural killer cells (Fig. 5K) by ssGSEA analysis, which maker genes for celltypes were shown in Table S7. And activated CD8^+^ T cells showed a trend of negative association with riskscore (*p* = 4.3e-12, *r* = − 0.24) (Fig. 5L). Moreover, IFN_TAMs were predictive of OS in GSE181063 and GSE10846 datasets (*p* < 0.05) (Fig. 6A, B), indicating that patients with longer OS exhibited lower IFN_TAMs levels. While patients with higher LA_TAMs were associated with superior OS (*p* < 0.05) (Fig. 6A, B). Furthermore, IFN_TAMs were positively correlated with *PD-L1* gene expressions (*p* = 4.1e-68, *r* = 0.56, and *p* = 1.0e-15, *r* = 0.57) (Fig. 6A, B). When combining GLC score and IFN_TAM for risk stratification, it can effectively predict OS (*p* < 0.01) (Fig. 6C).

### ST landscape of DLBCL

To validate the prognostic role of glycolytic biomarkers and IFN_TAM at the spatial transcriptomic level, as well as the relationship between glycolytic levels, IFN_TAM, and CD8^+^ T cell infiltration, spatial transcriptomic analysis was performed on tissue samples from 10 patients with DLBCL before R-CHOP treatment.

Firstly, quality control analysis of ten DLBCL samples revealed the detection of 4275–4992 spots, with a median of 4584–7343 detected genes per spot and sequencing saturation ranging from 53 to 86% (Table S8). The nCounts of ten samples were depicted in Fig. S5. Post-harmony integration, distinct cell types exhibited clear segregation, and patient sample distributions were effectively integrated compared to pre-harmony condition (Fig. S6). Based on PCA clustering in Fig. S7A, along with referencing single-cell annotation databases (CellMarker and PanLao DB), nine distinct cell types were identified. Notably, B cells and macrophages exhibited higher nCounts compared to other cell types (Fig. S7B). Representative cell markers are outlined in Figs. S7C, S8 and Table S9 for B cell, T cell, NKT cell, fibroblast, macrophage, neutrophil, plasma cell, muscle and endothelial cells. Consistency between the results from CARD deconvolution and manual annotation was illustrated in Fig. S9, such as in the representative data shown in Fig. S7D for sample 2. The distribution of cell types across samples is detailed in Table S10 and Fig. S10A, B.

Regarding intratumoral and intracellular heterogeneity, the ITH scores were significantly higher in germinal center B-cell (GCB) samples compared to non-GCB samples across all cell types (Fig. S10C, D; Table S11). InferCNVs analysis indicated that B cells possessed the highest CNV score, with malignant cells (B cells) exhibiting higher CNV scores (*p* < 0.0001) compared to benign cells (non-B cells) (Fig. S11A-C). Similar trends were observed in representative samples, such as sample 2 (Fig. S11D), with notable variations observed in chromosome 3, visually represented in Fig. S11E, F. These demonstrated the concordance between the inferCNV and SPATA2 results, as well as the consistency between manual annotation and CARD-identified cell clusters.

### Validation of prognosis value for glycolysis markers and IFN_TAM in ST, IHC, and mIF cohorts

**Fig. 7:**
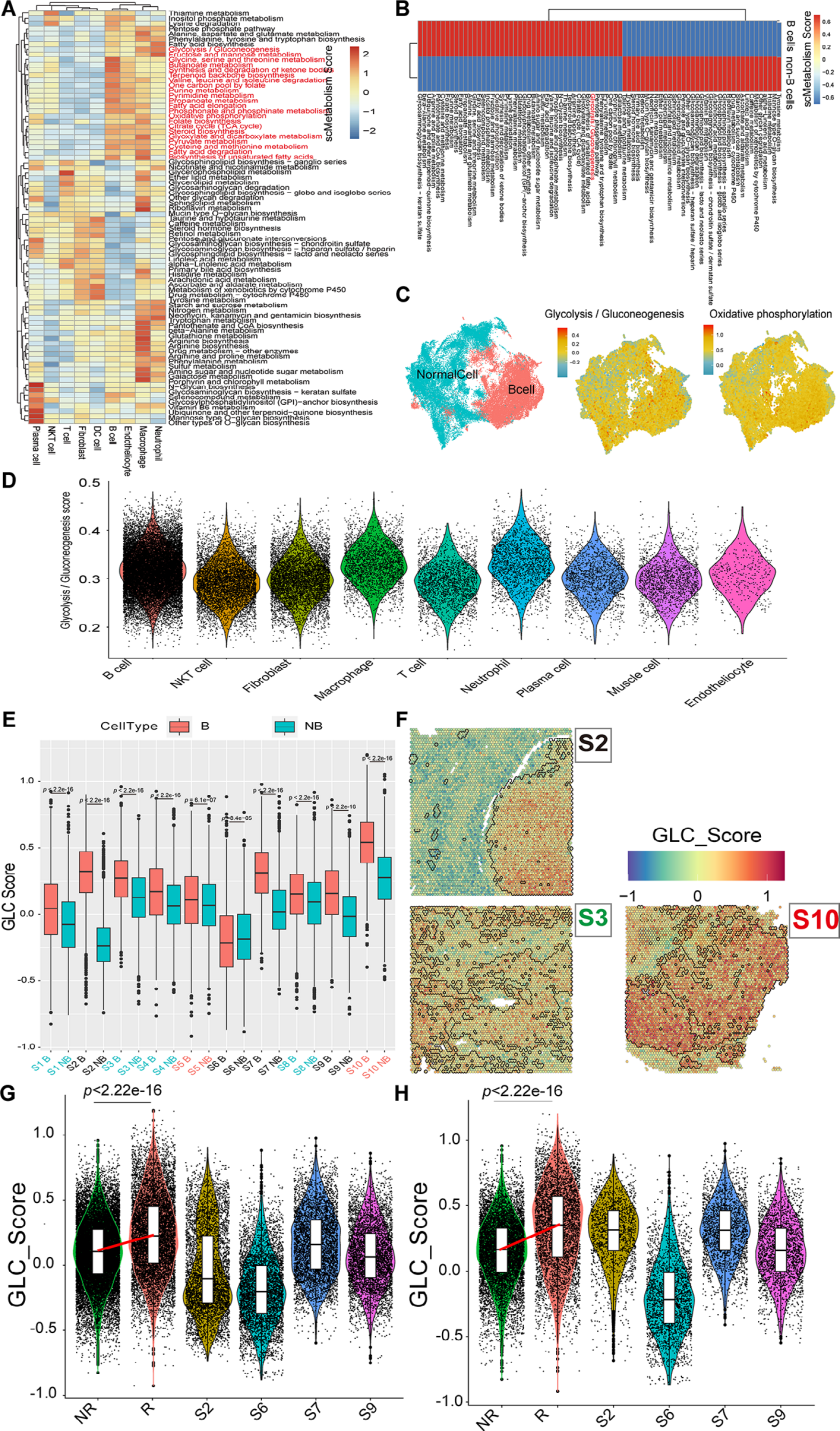
Metabolism altas and GLC score prognosis value validation of DLBCL in spatial transcriptomics. **A-B.** Metabolism enrichment of different cell types, and B cells and normal cells by heatmap. **C.** Highly expressed hallmark pathway scores of B cells using UMAP plot. **D.** Violin plot of glycolysis / gluconeogenesis pathway score across cell types. **E.** Comparison of GLC score in B cells and non-B cells. **F.** Spatial plot of GLC score in 10 DLBCL samples. **G-H.** Violin plot of GLC score in all cells and B cells between NR (*n* = 4), R (*n* = 2) and other samples (*n* = 4) *(Abbreviation*: GLC: glycolysis; DLBCL: diffuse large B-cell lymphoma; UMAP: uniform manifold approximation and projection; NB: non-B cell; R: relapsed patients, patients without EFS24; NR: non-relapsed patients, patients with EFS24. * *p* < 0.05, ** *p* < 0.01, *** *p* < 0.001, **** *p* < 0.0001, ns, not significant.)

Functional enrichment analyses for nine cell types, including comparisons between B cells and non-B cells, revealed consistent outcomes with scRNA-seq results. Notably, B cells exhibited enrichment in E2F-targets, G2M-checkpoint, MYC-targets, DNA-repair, and mTORC1-signaling hallmark pathways (*p* < 0.05) (Fig. S7E, F), which were reflected in the UMAP plot (Fig. S7G, H). Moreover, consistent with scRNA-seq findings, both B cells and macrophages displayed elevated metabolic activity (Fig. 7A), with higher activity in glycolysis/gluconeogenesis and oxidative phosphorylation metabolism observed in B cells compared to non-B cells (Fig. 7B, C). The glycolysis/gluconeogenesis score calculated using scMetabolism confirmed this observation (Fig. 7D). Five of seven glycolysis/gluconeogenesis marker genes, *ENO1*, *PPIA*, *STMN1*, *PKM* and *CDK1* were identified in ST. The glycolysis/gluconeogenesis (GLC) score based on these genes consistently showed higher scores (*p* < 0.0001) in B cells across all ten samples when compared to non-B cells (Fig. 7E, F). Notably, GLC scores were higher in R samples (*n* = 2) compared to NR samples (*n* = 4) (*p* < 0.001) in all cells and B cells (Fig. 7G, H).

**Fig. 8:**
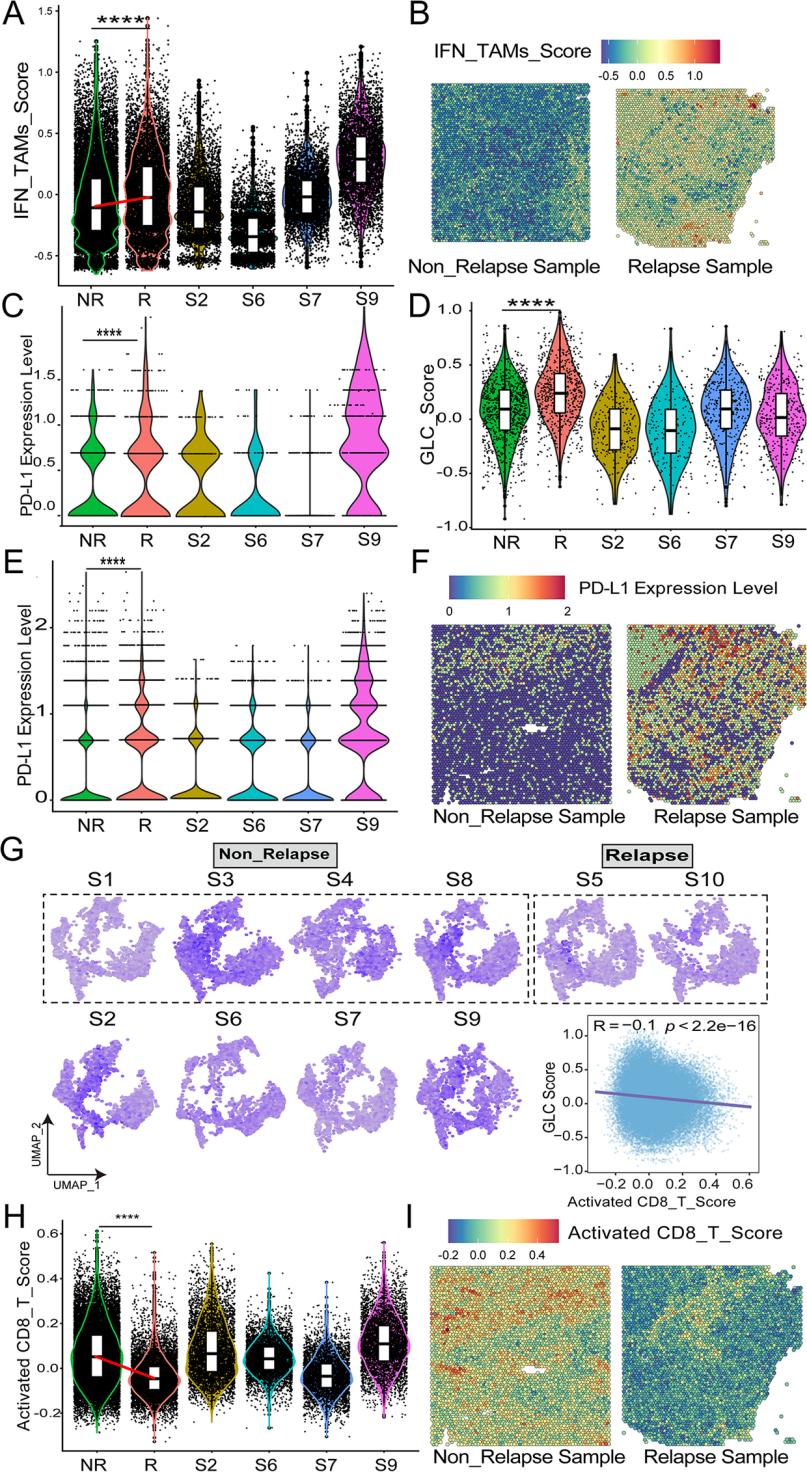
DLBCL samples with high glycolysis / gluconeogenesis activity were characterized by immunosuppressive microenvironment in spatial transcriptomics. **A-B.** Vlnplot and representative spatial plots of IFN_TAMs between NR (*n* = 4), R (*n* = 2) and other samples (*n* = 4). **C-D.** PD-L1 expression and GLC score of TAMs in samples by violin plot. **E.** Vlnplot of PD-L1 expression in all cells between NR (*n* = 4), R (*n* = 2) and other samples (*n* = 4). **F.** Representative spatial plots of PD-L1 expression in NR (S3) and R (S10) group. **G-H.** UMAP and violin plot of activated CD8^+^ T score in NR (*n* = 4), R (*n* = 2) and other samples (*n* = 4), along with the correlation between GLC score and activated CD8^+^ T score. **I.** Representative spatial plots of activated CD8^+^ T score in NR (S3) and R (S10) group (*Abbreviation*: DLBCL: diffuse large B-cell lymphoma; UMAP: uniform manifold approximation and projection; TAMs: tumor-associated macrophages; GLC: glycolysis; R: relapsed patients, patients without EFS24; NR: non-relapsed patients, patients with EFS24. Mann-Whitney test was performed between groups. * *p* < 0.05, ** *p* < 0.01, *** *p* < 0.001, **** *p* < 0.0001, ns, not significant.)

Concerning the exhausted immune microenvironment, evaluating IFN_TAMs infiltration revealed higher levels (*p* < 0.0001) in R samples (Fig. 8A, B), higher expression of PD-L1 in TAMs of R samples (Fig. 8C), paralleling the trend observed in GLC scores (*p* < 0.0001) (Fig. 8D).This trend was consistent across all cell types, as exemplified by the representative NR sample S3 and R sample S10 in spatial plots (Fig. 8E, F). Furthermore, the comparison of activated CD8^+^T cell scores between R and NR samples exhibited lower scores (*p* < 0.0001) in R samples (Fig. 8G, H). The activated CD8^+^ T score demonstrated a trend of negative association with the GLC score (*R* = -0.1, *p* < 2.2e-16) (Fig. 8G). This trend was visually represented in spatial plots, exemplified by the representative NR sample S3 and R sample S10 (Fig. 8I). Interestingly, the trend of activated CD8^+^T cell scores contrasted with the GLC scores across all ten samples (Figs. 7H and 8H).

**Fig. 9:**
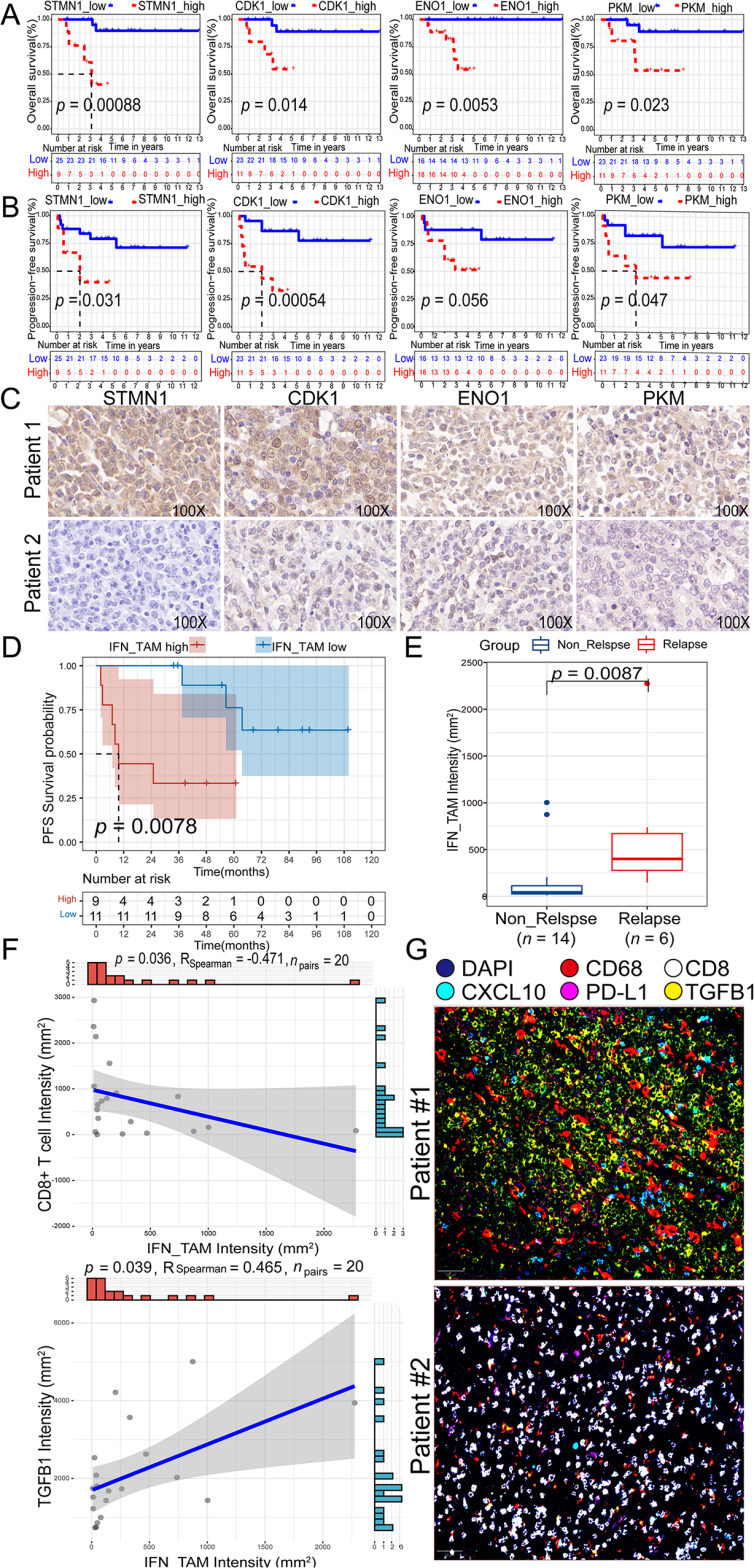
Prognostic value of four glycolysis / gluconeogenesis (STMN1, ENO1, CDK1, PKM, and PPIA) proteins in IHC cohort (*n* = 34, 100X) and IFN_TAMs (CD68^+^CXCL10^+^PD-L1^+^) in mIF cohort (*n* = 20, 10X). **A-B.** Kaplan–Meier curves of OS and PFS according to STMN1, CDK1, ENO1 and PKM proteins expression. **C.** Representative IHC staining of STMN1, CDK1, ENO1 and PKM in patient 1 (PFS = 7 months, OS = 9 months) and patient 2 (PFS = 135 months, OS = 135 months). **D.** Kaplan-Meier analysis for PFS based on IFN_TAMs intensity. **E.** Comparison of IFN_TAMs intensity in relapse and non_relapse groups. **F.** Correlation of IFN_TAMs’ intensity, CD8^+^ T cells’ intensity, and TGFβ1 intensity. **G.** Representative mIF staining of IFN_TAMs and CD8^+^ T cells in patient #1 (PFS = 2.7 months) and patient #2 (PFS = 90 months). (*Abbreviation*: IHC: immunohistochemistry; IFN_TAMs: interferon-primed tumor-associated macrophages; mIF: multiple immunofluorescence; OS: overall survival; PFS: progression -free survival, Relapse: relapsed patients, patients without EFS24; Non_Relapse: non-relapsed patients, patients with EFS24. Mann-Whitney test was performed between groups.)

Five glycolysis / gluconeogenesis proteins (STMN1, CDK1, ENO1, PKM and PPIA) expression were assessed in 34 DLBCL FFPE samples (Table S1, Fig. 9, and S12). Representative examples of STMN1, CDK1, ENO1, PKM and PPIA expression ranging from negative, weak, moderate to strong were shown in Fig. S12A. The expression of STMN1, CDK1, ENO1, and PKM proteins showed predictive value for OS and PFS (*p* < 0.05) (Fig. 9A, B). Representative IHC staining of STMN1, CDK1, ENO1, and PKM in a patient with short PFS and OS (PFS = 7 months, OS = 9 months) and a patient with longt PFS and OS (PFS = 135 months, OS = 135 months) were shown in Fig. 9C. PPIA expression couldn’t predict OS (*p* > 0.05), while showed predictive value for PFS (*p* < 0.05) (Figure S12B). And vlnplot exhibited that all of these five markers had higher levels in B cell type in GSE182434 (Fig. S12C).

In the mIF cohort, the intensity of IFN_TAMs demonstrated predictive value for PFS (*p* < 0.01) (Fig. 9D). Additionally, the intensity of IFN_TAMs was higher in patients who experienced relapse (*p* < 0.01) (Fig. 9E). Moreover, samples with higher levels of IFN_TAMs exhibited lower CD8^+^ T cell infiltration (*p* < 0.05, *R* = -0.471) (Fig. 9F) and higher TGFβ1 infiltration (*p* < 0.05, *R* = 0.465) (Fig. 9F). Representative mIF staining of IFN_TAMs and CD8^+^ T cells in a patient with short PFS (PFS = 2.7 months) and a patient with long PFS (PFS = 90 months) are shown in Fig. 9G.

## Discussion

Several studies have focused on the glycolysis metabolism and DLBCL prognosis, including metabolism-associated gene signature and plasma metabolites [59–61]. 13 metabolic gene signatures were found to be associated with poor prognosis in DLBCL [59]. He et al. discovered and validated 14 metabolism-associated genes for the prognostic prediction in DLBCL [60]. And higher abundance of plasma malate, which was essential for cancer growth by contributing to elevated glycolytic flux, was found to be correlated with poorer survival [61]. The aforementioned studies were all based on bulk-RNA-seq for screening metabolic genes. However, whole tissues reflect average gene expression levels, failing to elucidate differences among various cellular heterogeneities within tumors. Single-cell transcriptomics and spatial transcriptomics effectively address these issues. Furthermore, these studies lack elucidation on the role of glycolytic metabolism levels in malignant cells, exploration of tumor immune microenvironment differences caused by metabolic heterogeneity, and independent validation of clinical samples.

This study comprehensively applied multi-omics to identify a highly malignant tumor cell type and IFN_TAMs. DLBCL tissues with high glycolysis activity exhibited an immunosuppressive microenvironment, manifested by abundant IFN_TAMs, and low CD8^+^ T cell infiltration. Through scRNA-seq, we identified highly malignant DLBCL cell subgroups with enhanced glycolysis, with seven glycolysis genes identified (*LDHA, TPI1, PPIA, STMN1, CDK1, ENO1*, and *PKM*). Additionally, IFN_TAMs showed high metabolic activity across all celltypes, closely interacting with high-malignancy tumor cells identified within datasets. The glycolysis score, derived from glycolysis genes and IFN_TAMs infiltration, emerged as an independent prognostic factor for DLBCL. ST confirmed elevated glycolytic activity in malignant cells (over 95% malignant B cells) and IFN_TAMs, particularly in relapsed patients. Prognostic value of four glycolysis genes (*STMN1, CDK1, ENO1*, and *PKM*) was further validated by IHC, emphasizing their predictive power for overall and progression-free survival. This comprehensive analysis sheds light on DLBCL development mechanisms and metabolic targets, offering insights for precise immune therapies targeting tumor-specific metabolic pathways.

In our study, four glycolysis genes (*ENO1, STMN1, PKM*, and *CDK1*) have been previously reported to be associated with cancer progression and drug resistance. Enolase 1 (*ENO1*), plays a vital role as a glycolytic enzyme in cellular energy metabolism and is overexpressed in more than 70% of human cancers [62]. ENO1 promotes glycolytic metabolism, oncogenic signaling, tumor migration, invasion, and metastasis [63–65]. In lymphoma, ENO1 expression was generally high and being eight times higher than that observed in benign lymphoid tissues [66]. And ENO1 can promote tumor cell proliferation and alter the phosphatidylinositol 3-kinase/Akt signaling pathway between cells, mediating drug resistance [67]. In peripheral T-cell lymphoma, high ENO1 expression in tissues is positively correlated with low overall survival rates [68]. In DLBCL, our previous studies found that high ENO1 protein levels in plasma were positively correlated with disease progression within two years, lower PFS, and OS [69]. Consistent with our findings, higher ENO1 protein expression in DLBCL patients’ tumor tissues indicated poorer survival. Stathmin (*STMN1*) is a structural microtubule-associated protein that binds to tubulin dimers, preventing their aggregation and thus destabilizing microtubules. It is overexpressed in many malignant tumors, such as non-small-cell lung cancer, and hepatocellular carcinoma, serving as a biomarker for malignant progression, recurrence, and resistance to adjuvant therapy (e.g. paclitaxel and vinblastine) [70–72]. STMN1 is also highly expressed in hematological malignancies [73]. In follicular lymphoma, STMN1 can further serve as a sensitive marker to distinguish primary cutaneous follicular lymphoma from primary cutaneous marginal zone lymphoma [74, 75]. Pyruvate kinase (*PKM*) gene encodes two proteins, PKM1 and PKM2. PKM1 is upregulated in tissues requiring large energy supplies, such as heart, brain, and muscles, while PKM2 is expressed in all proliferating cells, especially tumors and embryonic tissues [76, 77]. Upregulated expression of PKM2 gene in cancer cells can confer resistance to drugs (e.g. cisplatin and erlotinib) [78, 79]. In DLBCL, high PKM2 protein levels were associated with recurrence and poor survival [80], which was consistent with our findings. Cyclin-dependent kinase 1 (*CDK1*) is a serine/threonine kinase that controls the cell cycle progression from the G2 phase to the M phase, playing crucial roles in controlling cell division [81]. Dysregulation of CDKs is considered a hallmark event in almost all cancer types and it was also associated with tumor chemoresistance [82].

The heightened glycolytic metabolism in invasive tumor cells induces hypoxia, lactate accumulation, and other factors, influencing the tumor immune microenvironment [83].TAMs constitute up to 50% of the immune cell population within tumor tissues, with M2 phenotype predominantly present, especially in hypoxic regions [84]. Previous studies have implicated TAMs in the tumorigenesis and invasive progression of DLBCL [22–24]. Meta-analyses revealed that high-density M2 TAMs within the tumor microenvironment are indicative of poorer OS in DLBCL [85]. Furthermore, Ma RY et al. identified IFN_TAMs through scRNA-seq, characterized by heightened glycolytic activity, while its role in DLBCL remains unexplored. The specific metabolic pathways of TAMs are closely associated with their phenotype and function. TAMs’ glycolytic metabolism progressively intensifies during tumor growth [86]. The interaction between lactate-mediated tumor cells and TAMs is reciprocal. Tumor-derived lactate activates HIF-1α to promote TAMs glycolysis, M2 polarization, and tumor-promoting functions [14]. Additionally, lactate derived from TAMs provides energy metabolic substrates, promoting tumor progression. For instance, bladder cancer cells can re-educate M2 TAMs through lactate secretion, activating HIF-1α to promote TGF-β secretion. M2 TAMs, in turn, can enhance bladder cancer cell glycolysis through transforming growth factor-beta (TGF-β) [87]. Furthermore, lactate can upregulate PD-L1 expression by increasing HIF-1α expression or modulating NF-κB signaling pathways in TAMs [16]. Tumor-derived hyaluronic acid fragments can also upregulate PFKFB3 expression in TAMs, promoting glycolysis and PD-L1 expression [17]. PD-L1 expression in tumor cells can also be elevated by TGF-β1 secreted by TAMs, which can upregulates PKM2 and activates STAT1 [20]. This metabolic shift and PD-L1 expression, can diminish CD8^+^ T cell and natural killer cell infiltration [88], suppress the memory and antitumor functions of CD8^+^ T cells [83], and facilitate the infiltration of immunosuppressive cells, ultimately impeding antitumor immunity and promoting tumor progression. The IFN_TAMs we identified exhibited heightened glycolytic activity, as well as elevated expression levels of *PD-L1, PFKFB3*, and *TGFB1* genes, coupled with increased activity of the HIF-1α transcription factor. Higher IFN_TAMs infiltration were also correlated with inferior survival in DLBCL.

Targeting glycolysis in cancer therapy is a burgeoning area of research for developing anticancer drugs. In solid tumor types, inhibiting tumor glycolysis can augment immune cell infiltration and enhance immunotherapy effectiveness [89]. Preclinical models have demonstrated the efficacy of a lactate transporter glycolysis inhibitor (AZD3965) in increasing immune cell infiltration in solid tumors, advancing to Phase I clinical trials [90]. Biological experiments also evaluated AZD3965 and OXPHOS inhibitor IACS-010759 effects on eight B-cell lymphoma cell lines, AZD3965 significantly reduced lymphoma cell growth (60-98%) across four cell lines compared to modest growth inhibition (5-45%) with oxidative phosphorylation inhibition [91]. And AZD3965 could also inhibit TAMs polarization [90]. This finding underscores the role of glycolysis regulation in B-cell lymphoma proliferation. Additionally, metformin shows promise in sensitizing treatment and improving DLBCL patient prognosis preclinically and clinically [92]. Moreover, ongoing studies target the identified genes (*ENO1, STMN1, PKM, CDK1*), with therapies like enolase 1 depletion demonstrating efficacy across various tumor types by inhibiting glycolysis, growth, proliferation, migration, metastasis, and sensitizing tumors to chemotherapy and radiotherapy [63]. Targeting STMN1 and PKM2 also shows promise in reducing cell growth, metastasis, and increasing tumor cell apoptosis [93, 94]. Despite these advancements, developing tumor-specific glycolysis inhibitors remains challenging amidst the critical role of the glycolysis pathway in immune cell function.

There are some limitations to this study. Firstly, both single-cell transcriptomics and spatial transcriptomics technologies inherently have dropout rates, which may result in the omission of genes with lower expression levels during glycolysis gene screening. And the inability of ST to effectively distinguish between malignant and non-malignant B cells is also a limitation. Moreover, further in vivo and in vitro experiments are necessary to fully understand the biological functions and potential mechanisms of glycolysis risk genes, IFN_TAMs, and their association with immune microenvironment.

## Conclusion

In summary, our study identified a highly invasive tumor cell and TAMs subgroup characterized by enhanced glycolysis metabolic activity in DLBCL. Glycolysis marker genes and IFN_TAMs were identified and constructed to be predictive of survival. Additionally, we observed that heightened glycolytic metabolism correlates with an immunosuppressive TME, marked by IFN_TAMs infiltration, and diminished CD8^+^ T cell infiltration.

### Electronic supplementary material

Below is the link to the electronic supplementary material.

Supplementary Material 1

## Data Availability

No datasets were generated or analysed during the current study.

## Abbreviations

DLBCL: Diffuse large B-cell lymphoma
R-CHOP: Rituximab: cycolphosphamide: doxorubicin: vincristine: and predni sone
IPI: International Prognostic Index
FDG-PET/CT: Fluorodeoxyglucose positron emission tomography computed tomography
TME: Tumor microenvironment
TAMs: Tumor-associated macrophages
scRNA-seq: Single-cell RNA-sequencing
ST: Spatial transcriptomics
GEO: Gene Expression Omnibus
CHCAMS: Cancer Hospital, Chinese Academy of Medical Sciences
TCGA: The Cancer Genome Atlas
GTEx: Genotype-Tissue Expression
FFPE: Formalin-fixed paraffin-embedded
IHC: Immunohistochemistry
EFS24: Event-free survival at 24 months
R: Relapsed
NR: Non-relapsed
PCA: Principal components analysis
PCs: Principal components
CNVs: Chromosomal copy-number variations
GSEA: Gene set enrichment analysis
UMAP: Uniform manifold approximation and projection
FC: Fold change
TF: Transcription factor
hdWGCNA: High-dimensional weighted gene co-expression network analysis
MSigDB: Molecular Signature Database
mRNA: Messenger RNA
GEPIA2: Gene Expression Profiling Interactive Analysis
OS: Overall survival
ROC: Receiver operating characteristic
ssGSEA: Single-sample Gene Set Enrichment Analysis
UMI: Unique molecular indentifier
ITH: Intratumoral heterogeneity
GLC: Glycolysis
HE: Hematoxylin-eosin
STMN1: Stathmin
ENO1: Enolase 1
PPIA: Peptidylprolyl isomerase A
PKM: Pyruvate kinase
CDK1: Cyclin-dependent kinase 1
AUC: Area under the curve
GCB: Germinal Center B-cell
