# Supplementary material for: Single-cell and spatial transcriptomics reveal a high glycolysis B cell and tumor-associated macrophages cluster correlated with poor prognosis and exhausted immune microenvironment in diffuse large B-cell lymphoma
Source: Biomark Res. 2024 Jun 5;12:58. doi: 10.1186/s40364-024-00605-w (PMC11155084; doi:10.1186/s40364-024-00605-w)
Supplement: Supplementary file 1 — Supplementary Material 1 [file 40364_2024_605_MOESM1_ESM.docx]

**Supplementary Figures**


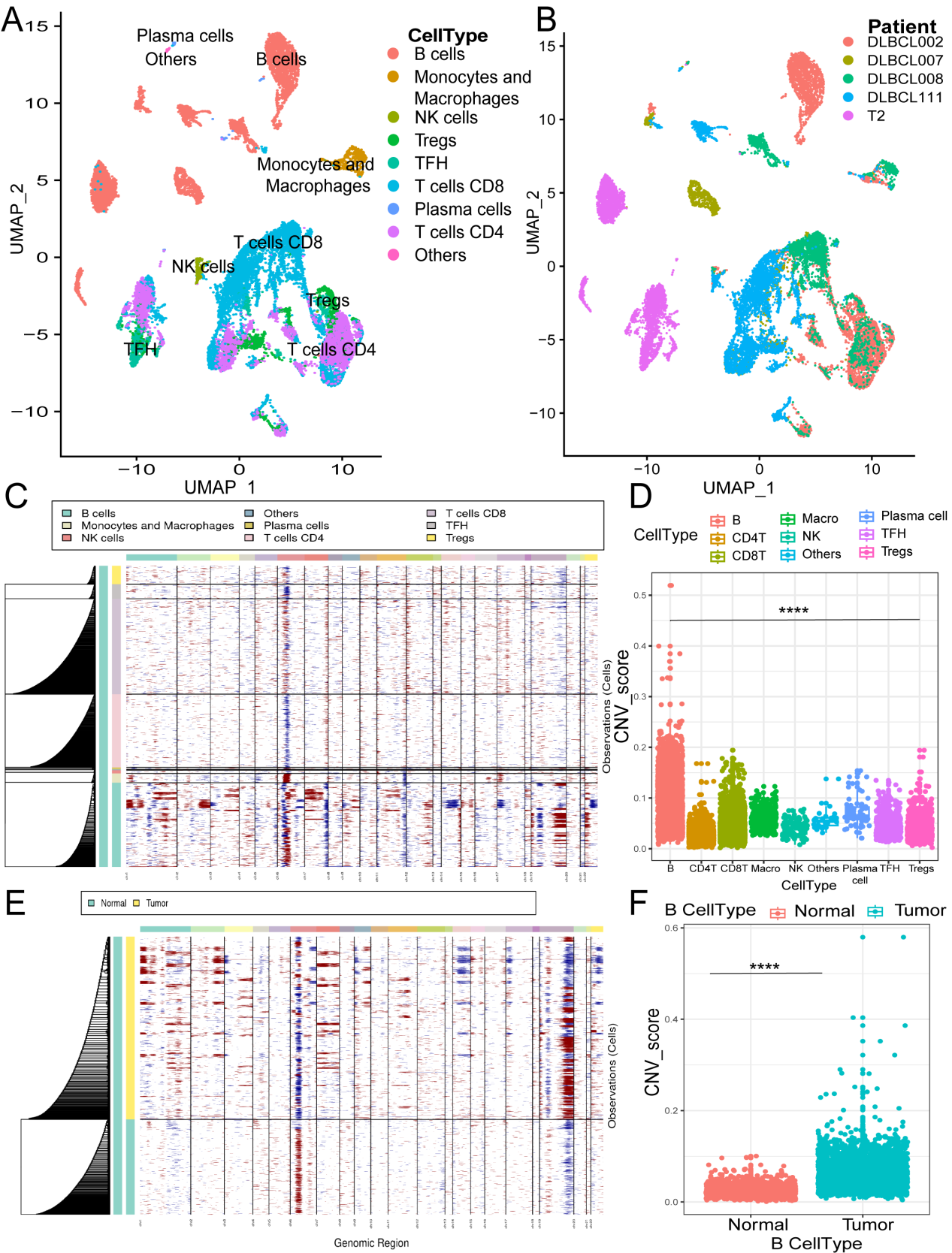


**Figure S1. Samples before harmony integration and inferred CNVs analysis in scRNA-seq.**

1. **B.** UMAP plot of cell types and samples before harmony integration. **C-D.** Heatmap and barplot of inferred CNVs score in cell types. **E-F.** Heatmap and barplot of inferred CNVs score in normal and tumor cells.

(*Abbreviation: CNVs: Chromosomal copy-number variations; scRNA-seq: single-cell RNA-sequencing; UMAP: uniform manifold approximation and projection; FH: follicular helper T cell; DLBCL: diffuse large B-cell lymphoma. Mann-Whitney test was performed between groups. **** p < 0.0001.*)


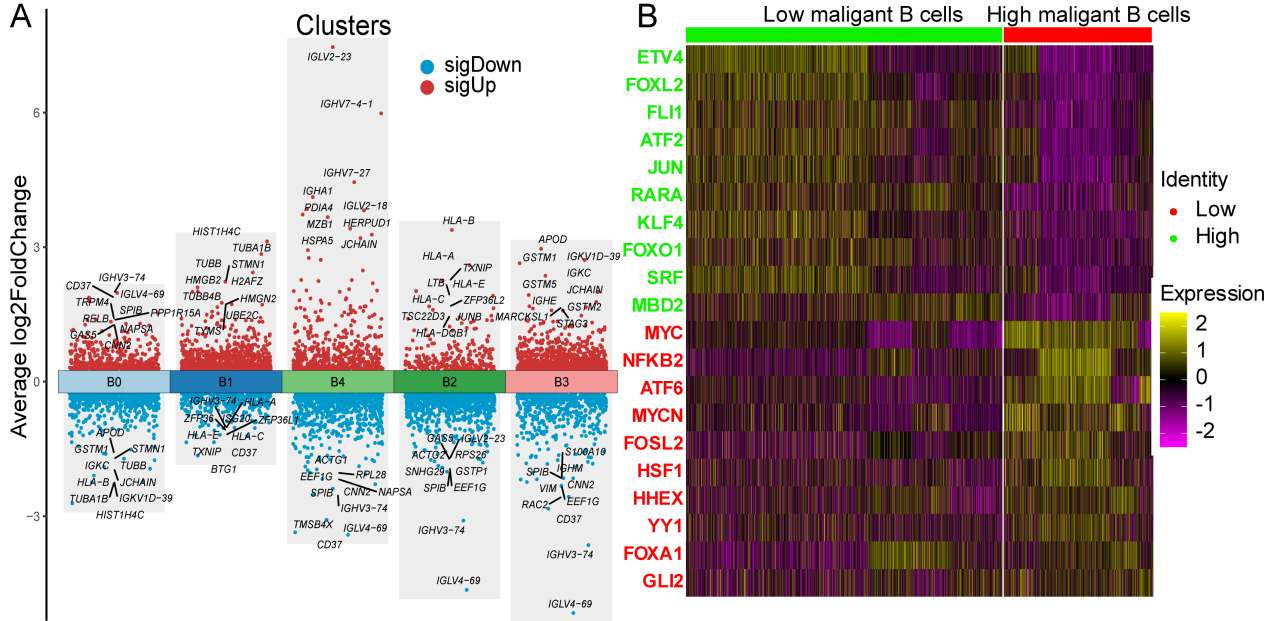


**Figure S2. B malignant subgroups identification in DLBCL scRNA-seq.**

1. Volcano plot of differential genes between the B0-B4 cells. **B.** Prediction of transcription factor activity in high and low maligant B cell subclusters by DoRothEA.

(*Abbreviation: DLBCL: diffuse large B-cell lymphoma; scRNA-seq: single-cell RNA-sequencing.*)


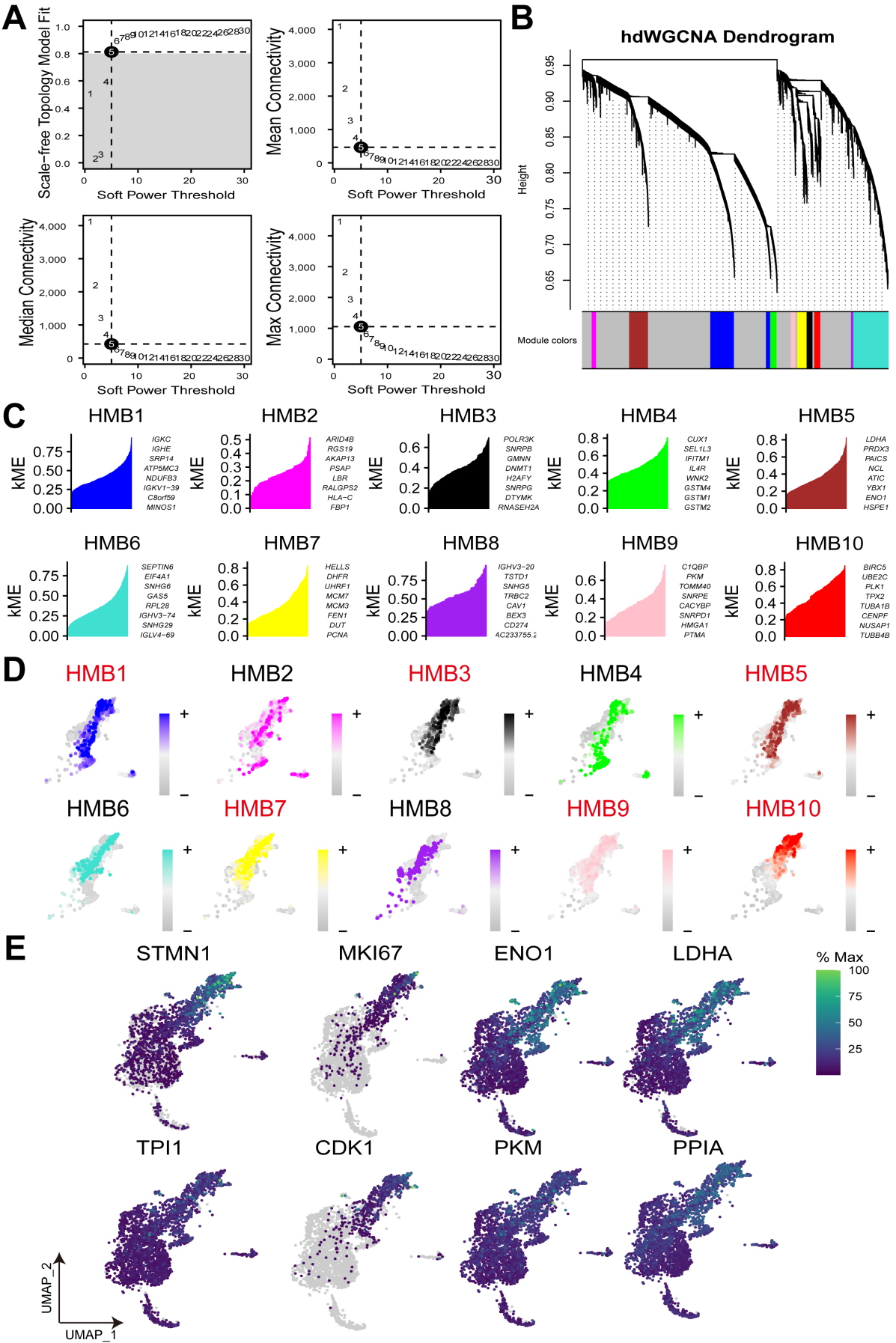


**Figure S3. B malignant subgroups identification in DLBCL scRNA-seq.**

**A-B.** Weighed gene co-expression network analysis was constructed among high malignant B cells. **C.** The frst 8 eigengenes of each module, ranked by eigengene-based connectivity (kME). **D.** Co-expression network of 10 modules .**E.** UMAP plot of MKI67 and eight glycolysis / gluconeogenesis gene markers.

*(Abbreviation: DLBCL: diffuse large B-cell lymphoma; scRNA-seq: single-cell RNA-sequencing; UMAP: uniform manifold approximation and projection; HMB: high malignant B cells.)*

**
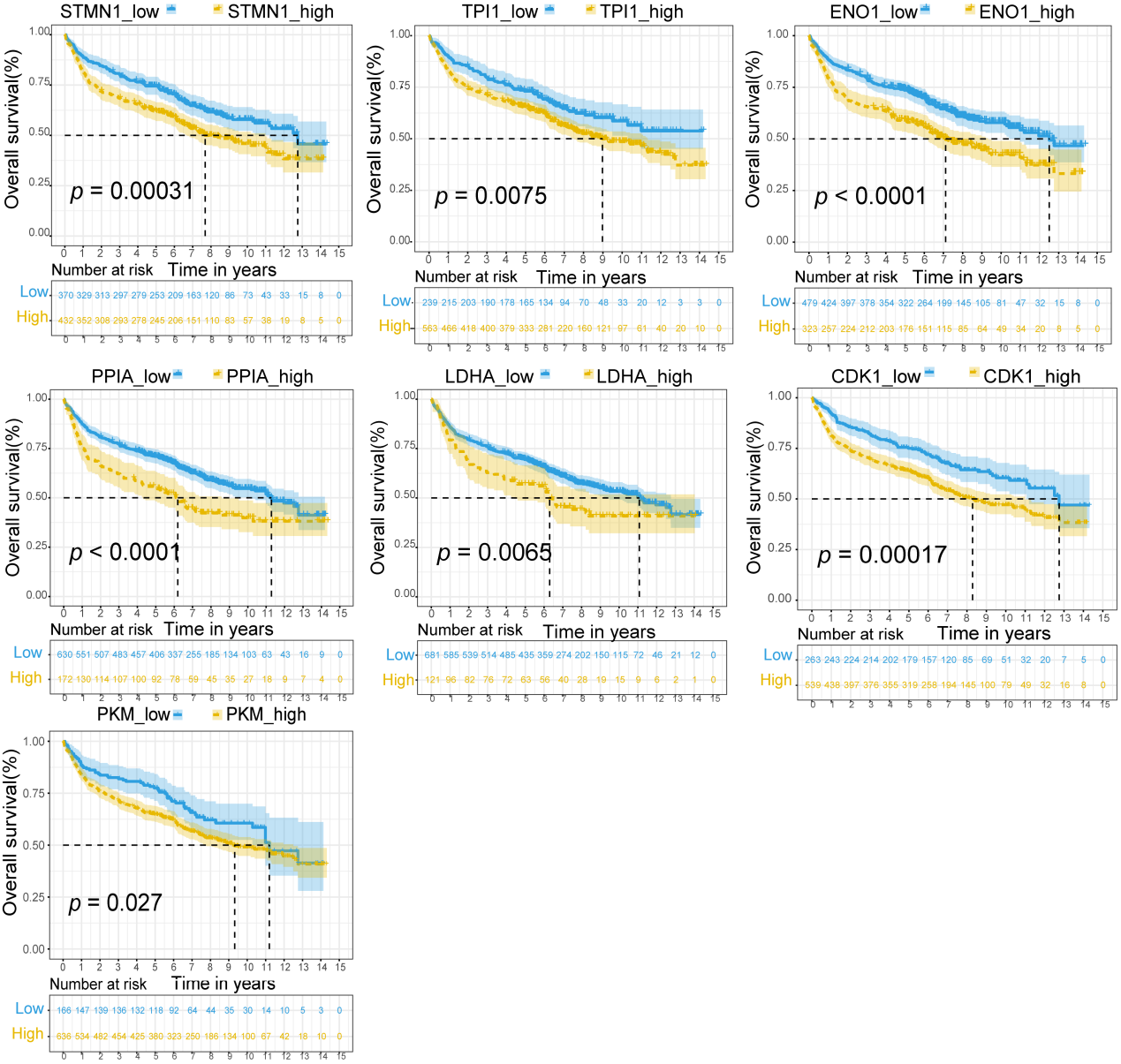
**

**Figure S4. Performance of seven glycolysis / gluconeogenesis markers (*PPIA, STMN1, ENO1, TPI1, LDHA, PKM* and *CDK1*) in predicting OS in GSE181063 (*n* = 802) and GSE10846 (*n* = 164).**

(*Abbreviation: OS: overall survival.*)


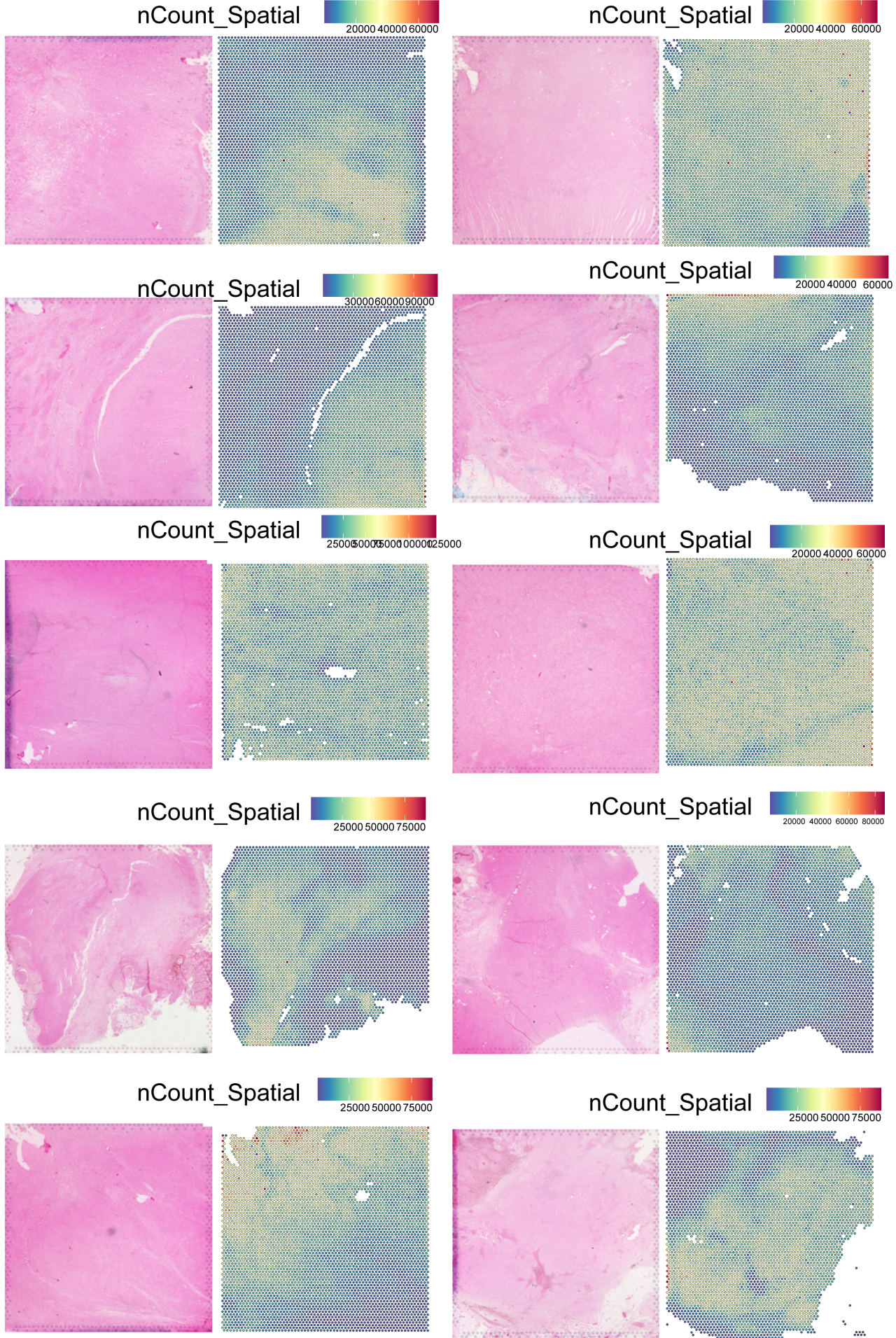


**Figure S5. Hematoxylin eosin staining and nCount spatial plots of 10 samples in spatial transcriptomics.**


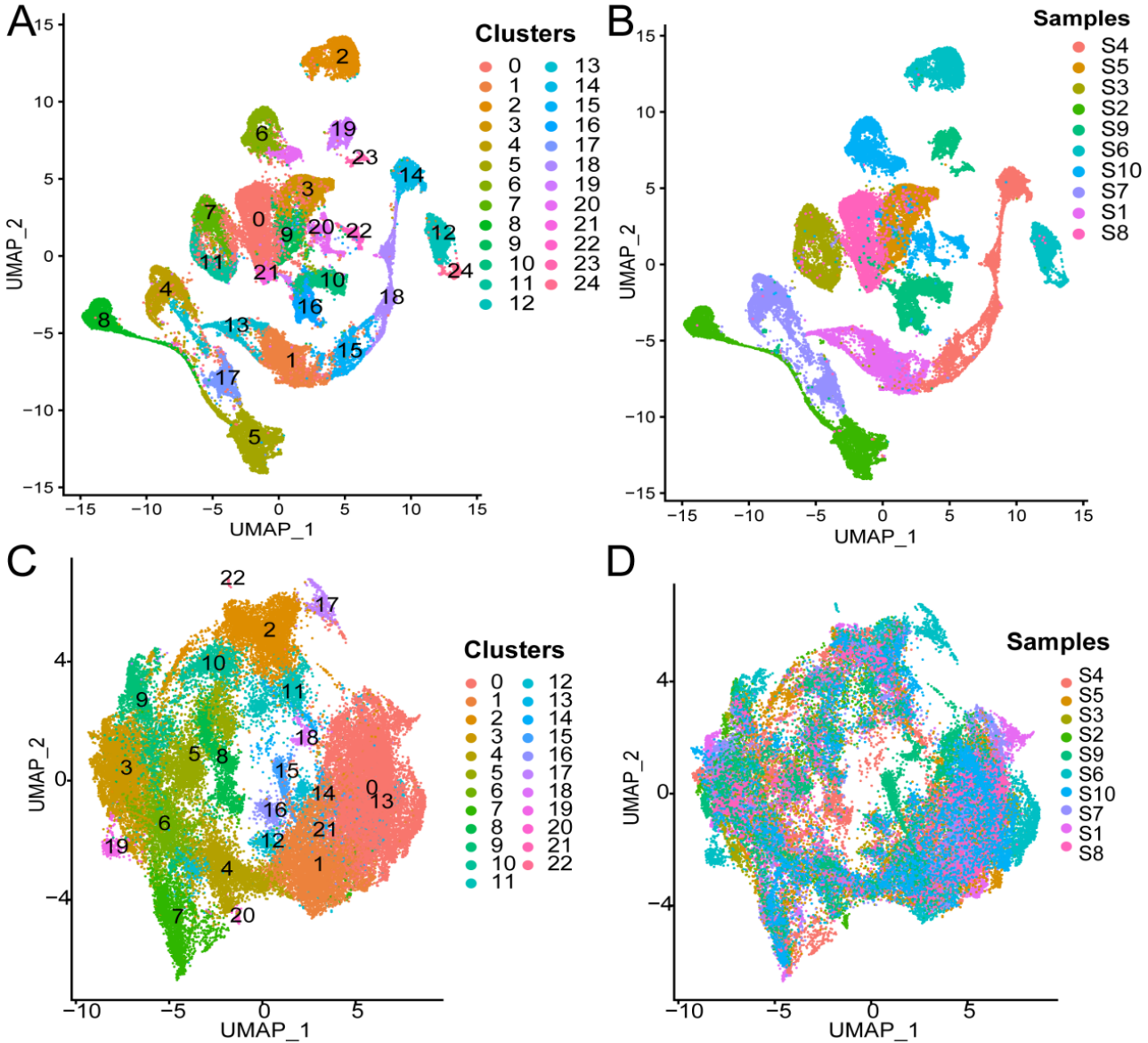


**Figure S6. DLBCL samples before and after harmony integration in ST.**

1. **B.** UMAP plots of PCA clustering and samples before harmony integration. **C-D.** UMAP plots of PCA clustering and samples after harmony integration.

(*Abbreviation: DLBCL: diffuse large B-cell lymphoma; ST: spatial transcriptomics; UMAP: uniform manifold approximation and projection; PCA: principal components analysis.*)

**
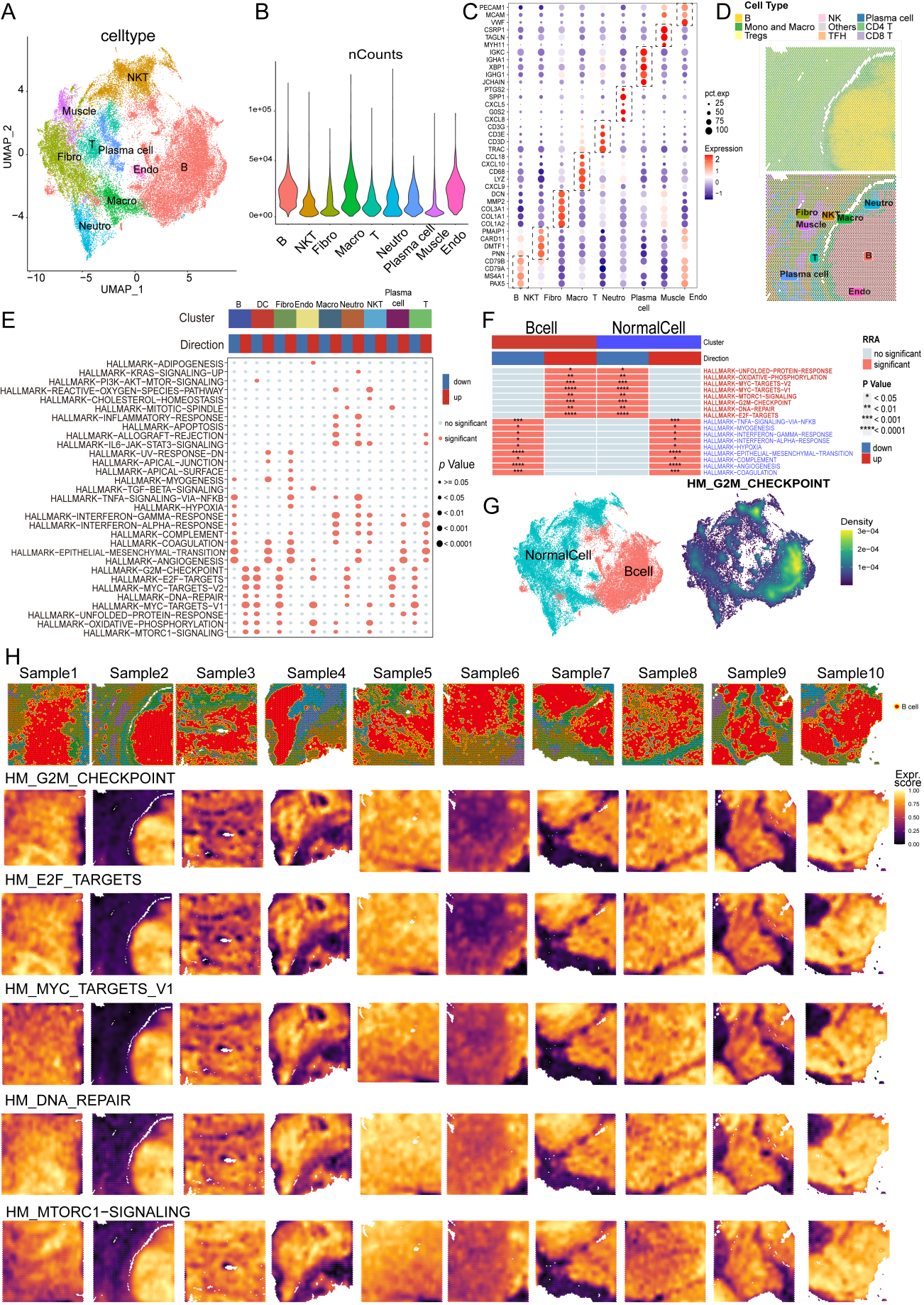
**

**Figure S7. Spatial transcriptomics cell types annotation and functional enrichment after harmony of DLBCL samples (*n* = 10).**

1. UMAP plot of cell types. **B.** nCounts in different celltypes. **C.** Dot plot for expression level of cell markers across cell types. **D.** Representative (S2) cell types annotation by CARD deconvolution and manual annotation. **E-F.** Hallmark and pathways of cell types, and B cells and normal cells using GSEA. **G.** UMAP plot of Hallmark_G2M_checkpoint pathway scores. **H.** Highly expressed hallmark pathways of B cells in 10 samples using spatial plot.

(*Abbreviation: DLBCL: diffuse large B-cell lymphoma; TFH: follicular helper T cell; UMAP: uniform manifold approximation and projection; GSEA: gene set enrichment analysis.*)


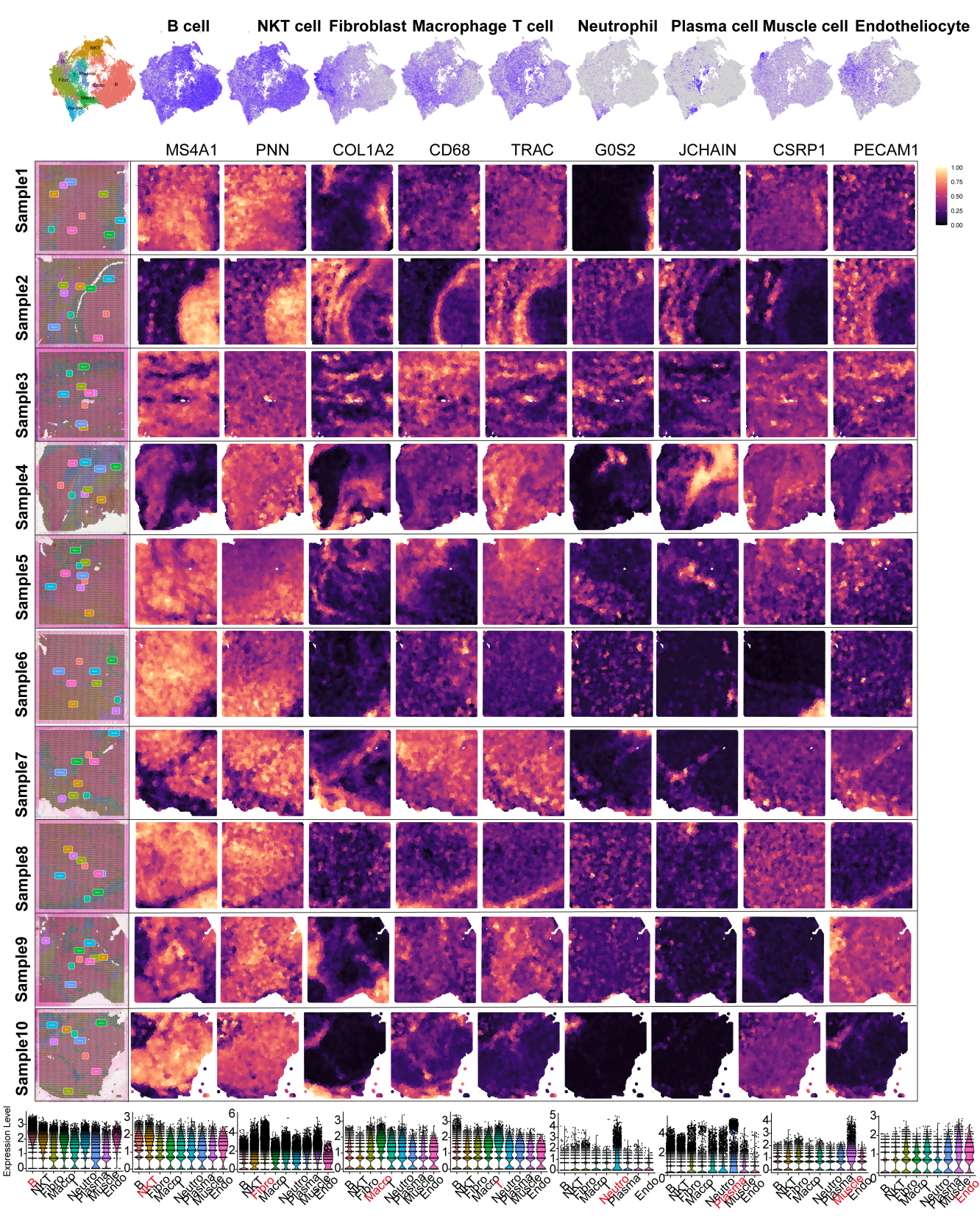


**Figure S8. Representative spatial plots of celltype markers in spatial transcriptomics.**


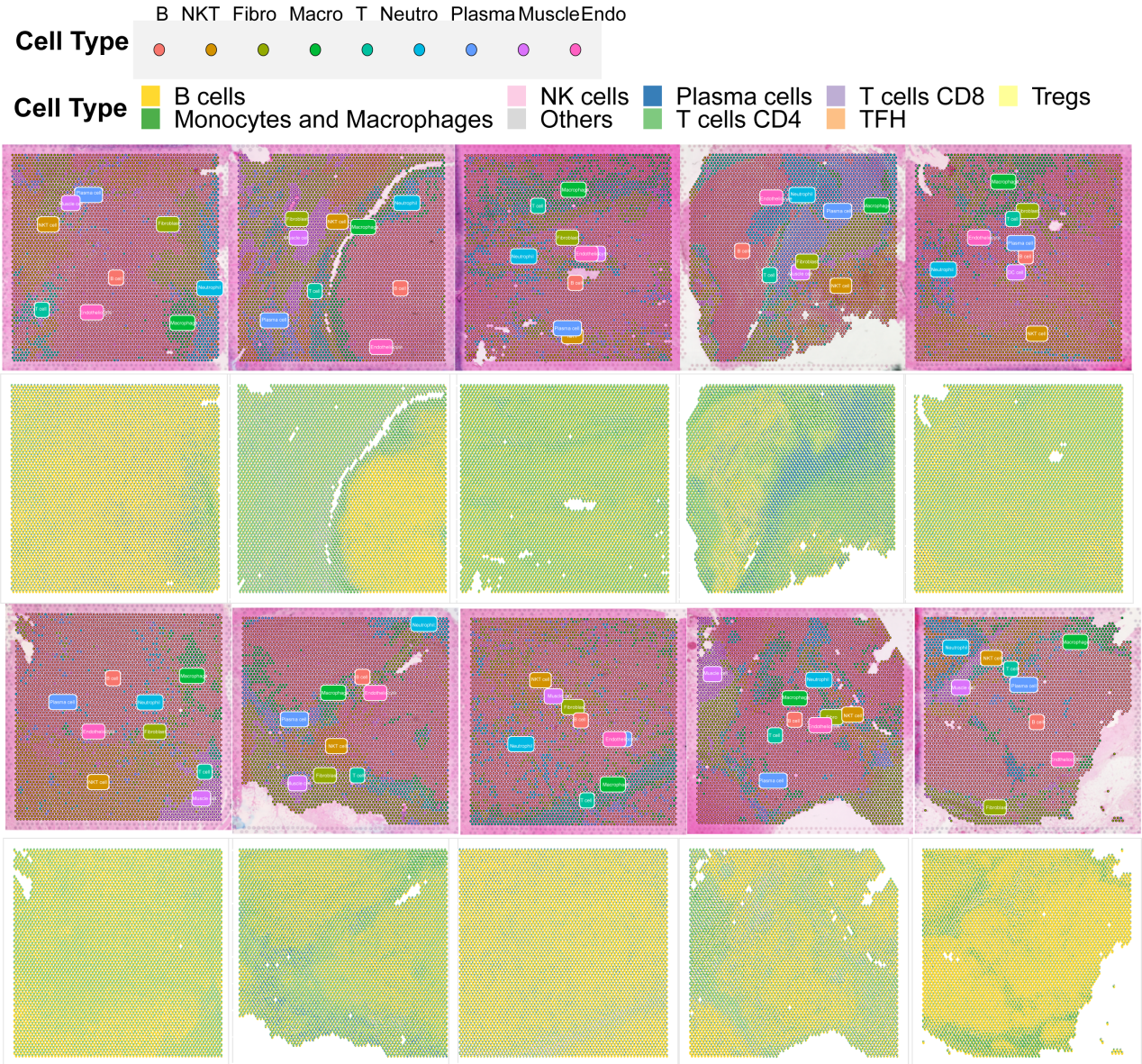


**Figure S9. Celltypes annotation by CARD deconvolution and manual annotation in spatial transcriptomics.**

**
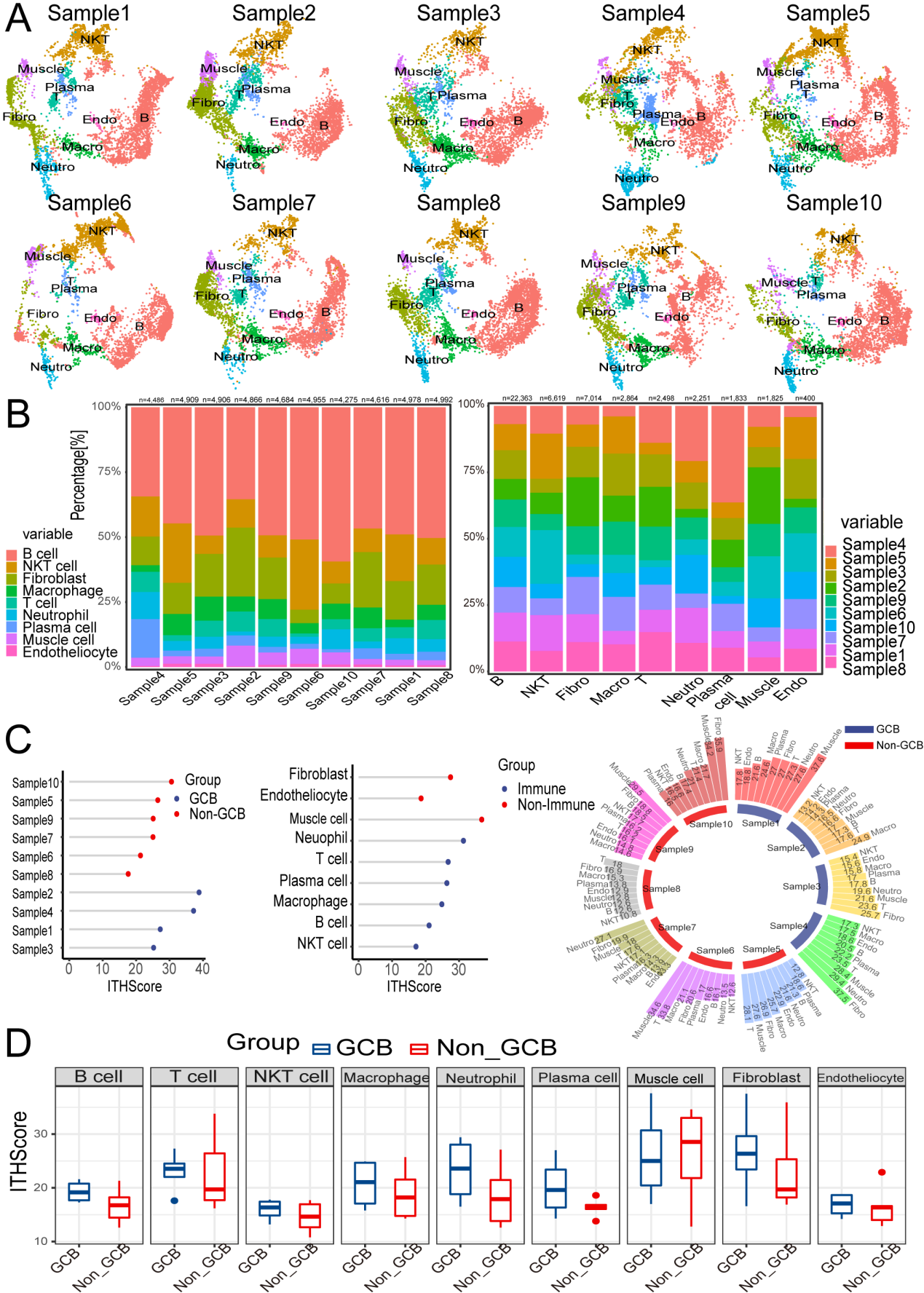
**

**Figure S10. Spatial transcriptomics to identify intra- and inter- tumor heterogeneity in DLBCL.**

1. **B.** UMAP and stacked barplot of cell proportion among samples. **C-D.** Comparison of ITH score between GCB and non-GCB, immune and non-immune cell types.

(*Abbreviation: DLBCL: diffuse large B-cell lymphoma; UMAP: uniform manifold approximation and projection; ITH: intratumoral heterogeneity; GCB : Germinal Center B-cell.*)

**
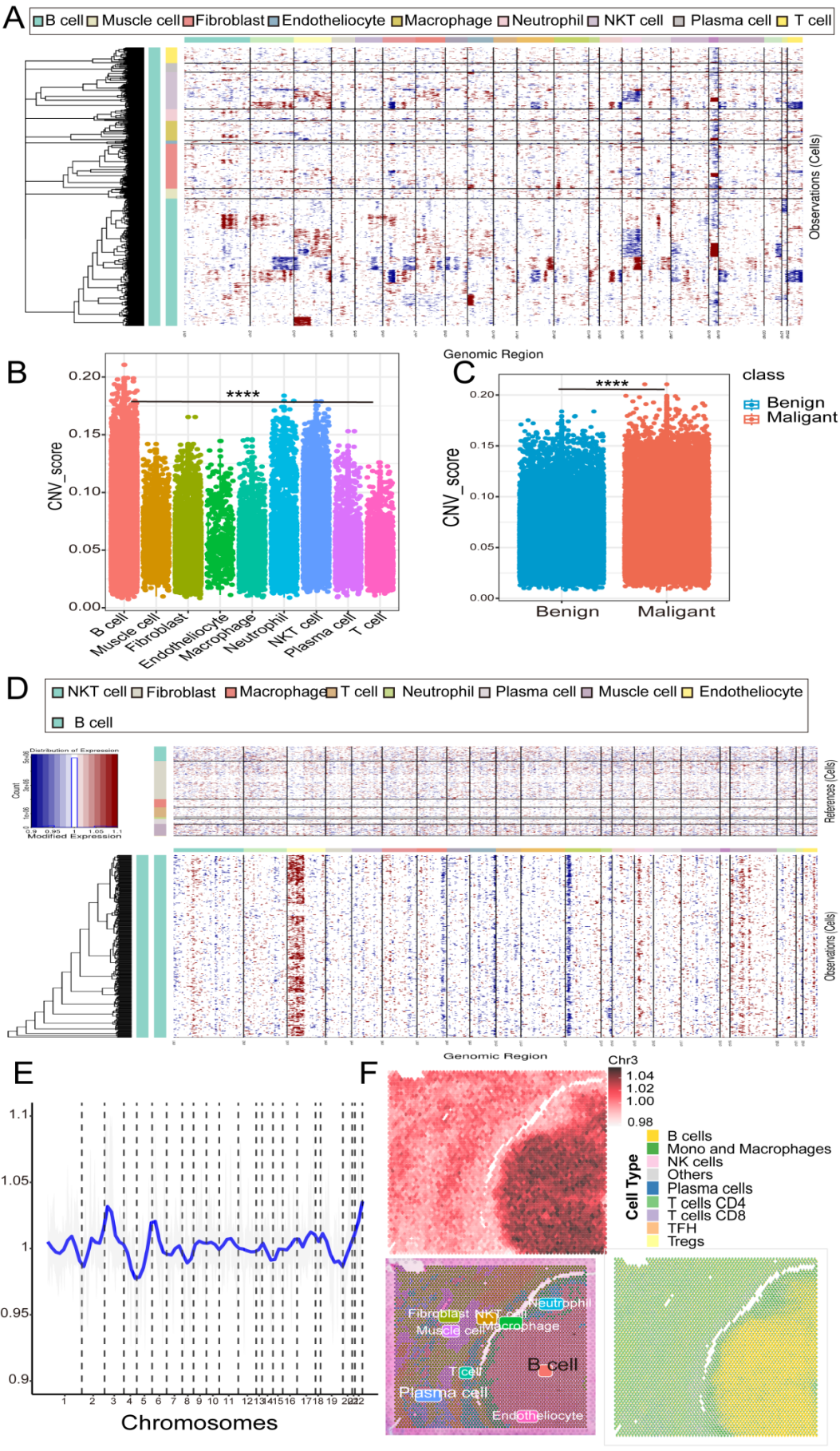
**

**Figure S11. Chromosomal landscape of inferred CNVs among DLBCL cell types in ST.**

**A-B.** Heatmap and barplot of inferred CNVs score in celltypes. **C.** Barplot of inferred CNVs score in benign and malignant cells. **D.** Heatmap of inferred CNVs score in celltypes of representative sample (S2). **E-F.** The frequency of CNVs on different chromosomes in line chart and spatial plot of CNVs in third chromosome of representative sample (S2) by SPATA2.

(*Abbreviation: CNVs: Chromosomal copy-number variations; DLBCL: diffuse large B-cell lymphoma; ST: spatial transcriptomics. Mann-Whitney test was performed between groups. **** p < 0.0001.*)



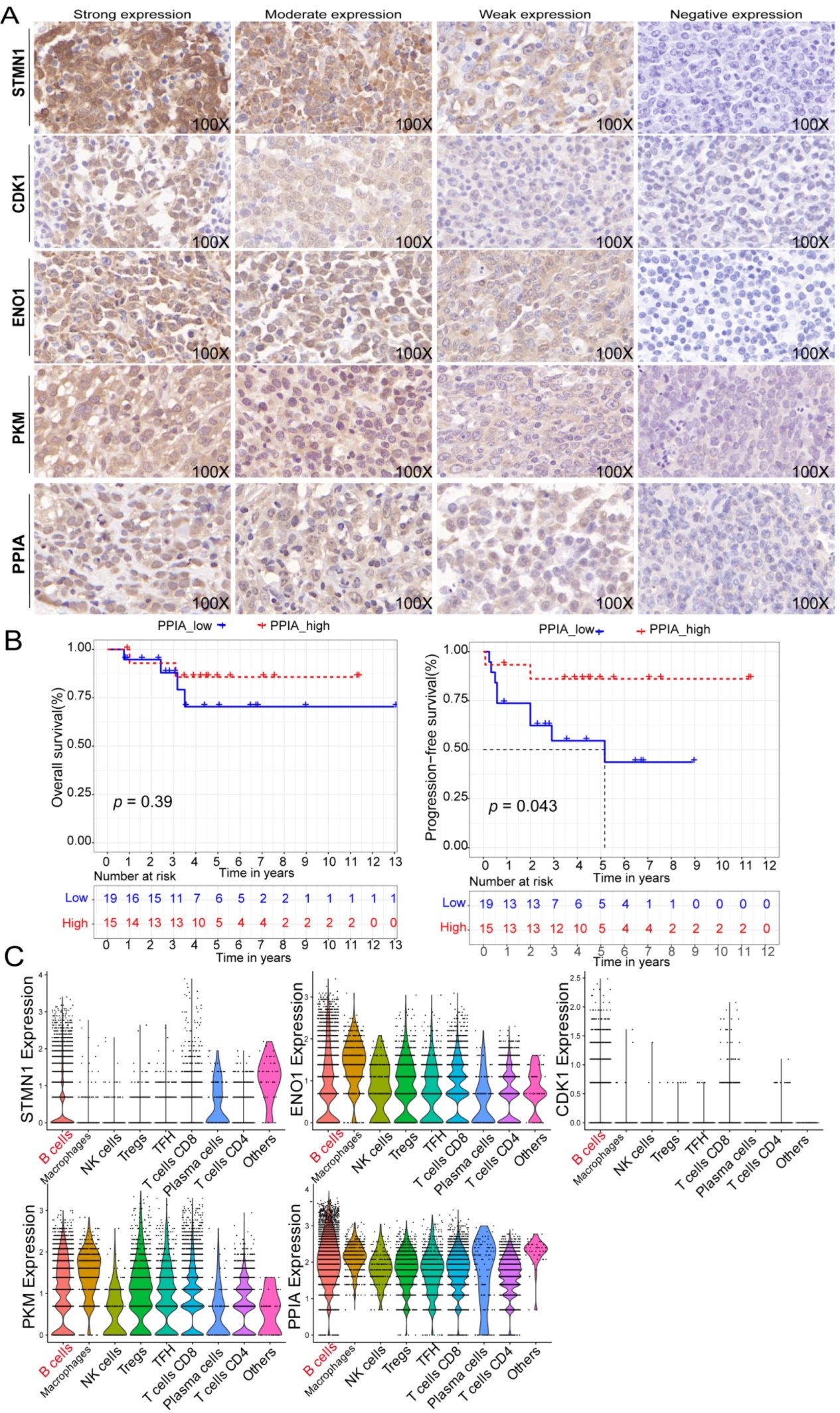



**Figure S12. Prognostic value of PPIA protein in IHC cohort (*n* = 34, 100X) and distributions of five markers across distinct cell types in single-cell RNA sequencing.**

1. STMN1, ENO1, CDK1, PKM, and PPIA expression levels from strong, moderate, weak to negative. **B.** Kaplan–Meier curves of OS and PFS according to PPIA protein expression. **C.** Vlnplot of 5 markers (STMN1, ENO1, CDK1, PKM, PPIA) in GSE182434.

(*Abbreviation: IHC: immunohistochemistry; OS: overall survival; PFS: progression -free survival.*)

**Supplementary Tables**

**Table S1.** Clinical characteristics of DLBCL patients in ST, IHC, and mIF cohorts.

**Table S2.** Detailed ST experimental procedures.

**Table S3.** Clinical characteristic and cell types distribution of GSE182434.

**Table S4.** Correlation coefficients of 24 metabolic pathways with CNV score and metabolism scores (mean ± standard deviation) in B, low malignant B and high malignant B cells.

**Table S5.** 101 genes identified in high malignant B cells.

**Table S6.** Differential genes identified in macrophage and monocytes subgroups.

**Table S7.** Markers for 30 cell types used in ssGSEA.

**Table S8.** Quality control of 10 DLBCL samples in ST.

**Table S9.** Annotation of 9 cell type markers for 10 DLBCL samples in ST.

**Table S10.** Number of spots of 9 cell types in each samples.

**Table S11.** ITH Scores of cell types across samples.

**Table S1.** Clinical characteristics of DLBCL patients in ST, IHC, and mIF cohorts.

| **Variables** | **ST** | **IHC** | **mIF** |
| --- | --- | --- | --- |
| Numbers | 10 | 34 | 20 |
| Progression |  |  |  |
| Yes, *n*% | 2(20) | 11(32.4) | 11(55) |
| No, *n*% | 8(80) | 23(67.6) | 9(45) |
| Gender |  |  |  |
| Male, *n*% | 7(70) | 20(58.8) | 13(65) |
| Female, *n*% | 3(30) | 14(41.2) | 7(35) |
| Age |  |  |  |
| ≤ 60 years, *n*% | 6(60) | 25(73.5) | 15(75) |
| > 60 years, *n*% | 4(40) | 9(26.5) | 5(25) |
| Ann Arbor stage |  |  |  |
| I/II, *n*% | 6(60) | 19(55.9) | 9(45) |
| III/IV, *n*% | 4(40) | 15(44.1) | 11(55) |
| ECOG |  |  |  |
| < 2, *n*% | 9(90) | 33(97.1) | 19(95) |
| ≥ 2, *n*% | 1(10) | 1(2.9) | 1(5) |
| Subtype |  |  |  |
| GCB, *n*% | 4(40) | 13(38.2) | 7(35) |
| Unclassified/ABC, *n*% | 6(60) | 21(61.8) | 13(65) |
| Number of extranodal sites |  |  |  |
| ≤ 1, *n*% | 9(90) | 23(67.6) | 9(45) |
| > 1, *n*% | 1(10) | 11(32.4) | 11(55) |
| IPI |  |  |  |
| 0/1, *n*% | 8(80) | 18(52.9) | 8(40) |
| 2, *n*% | 0(0) | 5(14.7) | 5(25) |
| 3, *n*% | 1(10) | 8(23.5) | 6(30) |
| 4/5, *n*% | 1(10) | 3(8.8) | 1(5) |

Abbreviation: DLBCL: diffuse large B-cell lymphoma; ST: spatial transcriptomics; IHC: immunohistochemistry; mIF: multiple immunofluorescences; ECOG: Eastern Cooperative Oncology Group; GCB: germinal-center B-cell-like; ABC: activated B-cell-like; IPI: International Prognostic Index.

**Table S2.** Detailed ST experimental procedures

| **1** | **Sample fixation and HE staining** |
| --- | --- |
|  | - Five-micrometer FFPE sections from 10 DLBCL samples were positioned on the capture areas of immunocytochemistry slides. Following a 2-hour incubation at 42℃ and subsequent air drying at room temperature, the slides underwent an additional drying period of 3 hours at 60℃. Hematoxylin (Dako, S330930-2) and Eosin (Sigma-Aldrich, HT110216) were employed for the HE staining process. Approximately 100 µL of 85% Glycerol (Thermofisher, 15514011) was applied, followed by the placement of a coverslip for tissue imaging. The coverslip was removed using a beaker filled with Milli-Q water. |
| **2** | **Probe hybridization** |
|  | - The Visium slide was inserted into a cassette, with 100 µL of 0.1 N HCl (Sigma-Aldrich, H1758) added to each well and incubated for 15 minutes at 42℃. Following the removal of HCl from each well, decrosslinking buffer was introduced, and incubation occurred at 95℃ for 1 hour. The subsequent step involved the Pre-hybridization process according to The Visium Spatial Gene Expression for FFPE reagent kit (10×Genomics,User Guide CG000407 Rev C, human transcriptome Product number 1000338), wherein 100 µL of Pre-hybridization mix was added to each well and incubated for 15 minutes at room temperature. Upon completion of this incubation, the pre- hybridization mix was removed, and 100 µL of Hybridization mix was added. The Visium slide was then incubated with the Hybridization mix at 50°C overnight. |
| **3** | **Probe ligation, release and extension, probe elution, and library preparation** |
|  | - The remaining steps of library preparation, encompassing probe ligation, probe release and extension, probe elution, and FFPE library construction, were conducted in accordance with the guidelines outlined in the "Visium Spatial Gene Expression for FFPE reagent kit" user guide (10× Genomics, User Guide CG000407 Rev C, human transcriptome Product number 1000338). The finalized libraries were sequenced using Novaseq6000 (Illumina), with read 1 and read 2 lengths set at 28 base pairs and 91 base pairs, respectively. |

*Abbreviation: ST: spatial transcriptomics; DLBCL: diffuse large B-cell lymphoma; HE: hematoxylin-eosin; FFPE: formalin-fixed paraffin-embedded.*

**Table S3.** Clinical characteristic and cell types distribution of GSE182434.

| **Sample/Celltypes** | **DLBCL002** | **DLBCL007** | **DLBCL008** | **DLBCL111** | **T2** |
| --- | --- | --- | --- | --- | --- |
| Tissue | DLBCL | DLBCL | DLBCL | DLBCL | Tonsil |
| COO | ABC | GCB | ABC | ABC | N/A |
| total_cell_count | 5534 | 1434 | 3000 | 4400 | 3829 |
| B cells | 83 | 0 | 0 | 0 | 1663 |
| High MB | 257 | 513 | 199 | 108 | 0 |
| Low MB | 1311 | 314 | 263 | 388 | 0 |
| Mono | 94 | 5 | 109 | 9 | 0 |
| DC_1 | 72 | 2 | 39 | 20 | 0 |
| LA_TAM | 27 | 3 | 72 | 9 | 0 |
| IFN_TAM | 3 | 3 | 57 | 2 | 0 |
| DC_2 | 18 | 2 | 15 | 6 | 0 |
| NK cells | 69 | 5 | 17 | 122 | 0 |
| Others | 0 | 5 | 0 | 41 | 0 |
| Plasma cells | 65 | 0 | 37 | 0 | 20 |
| T cells CD4 | 1720 | 131 | 437 | 966 | 1113 |
| T cells CD8 | 1177 | 302 | 1605 | 2399 | 332 |
| TFH | 209 | 14 | 0 | 72 | 567 |
| Tregs | 429 | 135 | 150 | 258 | 134 |

*Abbreviation: DLBCL: diffuse large B-cell lymphoma; COO: cell of origin subtypes; ABC: activated B-cell-like; GCB: germinal-center B-cell-like; MB: malignant B cells; DC: dendritic cell; TAM: tumor-associated macrophages; TFH: follicular helper T cell.*

**Table S4.** Correlation coefficients of 18 metabolic pathways with CNV score and metabolism scores (mean ± standard deviation) in B, low malignant B and high malignant B cells.

|  | **Pathway** | ***r*** | **B cell** | **LowMB** | **HighMB** |
| --- | --- | --- | --- | --- | --- |
| 1 | Terpenoid backbone biosynthesis | *0.47* | 0.08 ± 00 | 0.07 ± 0.15 | 08 ± 03 |
| 2 | Pentose phosphate pathway | *0.45* | 03 ± 03 | 0.46 ± 04 | 0.78 ± 0.34 |
| 3 | Fructose and mannose metabolism | *0.42* | 0.11 ± 0.17 | 08 ± 0.17 | 0.46 ± 0.19 |
| 4 | Steroid biosynthesis | *0.42* | 0.06 ± 03 | 0.02 ± 0.17 | 0.19 ± 07 |
| 5 | Glyoxylate and dicarboxylate metabolism | *0.40* | 0.07 ± 0.18 | 0.42 ± 02 | 0.63 ± 06 |
| 6 | Glutathione metabolism | *0.39* | 0.16 ± 0.15 | 0.33 ± 00 | 0.63 ± 0.33 |
| 7 | One carbon pool by folate | *0.37* | 0.01 ± 0.18 | 0.14 ± 0.19 | 0.45 ± 0.32 |
| 8 | N-Glycan biosynthesis | *0.36* | 0.11 ± 0.14 | 0.18 ± 0.12 | 08 ± 00 |
| 9 | Cysteine and methionine metabolism | *0.36* | 0.15 ± 0.18 | 0.50 ± 05 | 0.79 ± 09 |
| 10 | Synthesis and degradation of ketone bodies | *0.35* | -0.06 ± 02 | -0.03 ± 0.19 | 0.17 ± 09 |
| 11 | Pyruvate metabolism | *0.35* | 0.17 ± 00 | 0.73 ± 09 | 1.01 ± 0.33 |
| 12 | Galactose metabolism | *0.35* | -0.01 ± 0.15 | 0.02 ± 0.13 | 0.11 ± 0.14 |
| 13 | Glycolysis / Gluconeogenesis | *0.34* | 0.40 ± 05 | 0.94 ± 0.31 | 18 ± 0.37 |
| 14 | Purine metabolism | *0.34* | 0.17 ± 0.09 | 0.33 ± 0.13 | 0.45 ± 0.15 |
| 15 | Amino sugar and nucleotide sugar metabolism | *0.33* | -0.02 ± 0.10 | 0.05 ± 0.10 | 0.12 ± 0.11 |
| 16 | Propanoate metabolism | *0.33* | 0.14 ± 0.18 | 0.41 ± 00 | 0.61 ± 04 |
| 17 | Glycine, serine and threonine metabolism | *0.32* | -0.04 ± 0.13 | 0.19 ± 0.16 | 0.31 ± 0.18 |
| 18 | Citrate cycle (TCA cycle) | *0.31* | 0.16 ± 00 | 0.51 ± 02 | 0.69 ± 06 |

*Abbreviation: CNVs: Chromosomal copy-number variations; MB: malignant B cells.*

**Table S5.** 101 genes identified in high malignant B cells.

| **gene** | ***p_val*** | **avg_log2FC** | **pct.1** | **pct** | ***p_val_adj*** | **cluster** |
| --- | --- | --- | --- | --- | --- | --- |
| *HIST1H4C* | *5.60E-192* | 2.957 | 0.909 | 0.659 | *1.47E-187* | HighMB |
| *IGKV1D-39* | *0.00E + 00* | 2.733 | 0.474 | 0.038 | *0.00E + 00* | HighMB |
| *TUBA1B* | *2.55E-244* | 2.497 | 0.886 | 0.609 | *6.68E-240* | HighMB |
| *APOD* | *6.04E-231* | 223 | 0.521 | 0.094 | *1.59E-226* | HighMB |
| *GSTM1* | *0.00E + 00* | 2.108 | 0.448 | 0.025 | *0.00E + 00* | HighMB |
| *ATP5MC3* | *0.00E + 00* | 2.058 | 0.972 | 0.530 | *0.00E + 00* | HighMB |
| *STMN1* | *0.00E + 00* | 2.057 | 0.898 | 0.313 | *0.00E + 00* | HighMB |
| *TUBB* | *2.48E-207* | 2.013 | 0.927 | 0.708 | *6.51E-203* | HighMB |
| *TUBB4B* | *1.80E-281* | 1.933 | 0.804 | 0.323 | *4.72E-277* | HighMB |
| *JCHAIN* | *8.89E-189* | 1.927 | 0.536 | 0.142 | *2.33E-184* | HighMB |
| *H2AFZ* | *0.00E + 00* | 1.856 | 0.955 | 0.678 | *0.00E + 00* | HighMB |
| *IGKC* | *1.15E-129* | 1.747 | 0.630 | 0.316 | *3.03E-125* | HighMB |
| *HMGB2* | *3.76E-217* | 1.718 | 0.827 | 0.435 | *9.87E-213* | HighMB |
| *HSPD1* | *1.44E-299* | 1.709 | 0.955 | 0.617 | *3.77E-295* | HighMB |
| *HSPE1* | *3.94E-279* | 1.692 | 0.959 | 0.662 | *1.03E-274* | HighMB |
| *HSPA5* | *4.03E-160* | 1.674 | 0.888 | 0.550 | *1.06E-155* | HighMB |
| *ENO1* | *1.28E-299* | 1.666 | 0.965 | 0.688 | *3.35E-295* | HighMB |
| *LDHA* | *3.55E-298* | 1.663 | 0.952 | 0.653 | *9.32E-294* | HighMB |
| *TYMS* | *0.00E + 00* | 1.617 | 0.595 | 0.082 | *0.00E + 00* | HighMB |
| *PTTG1* | *0.00E + 00* | 1.566 | 0.795 | 015 | *0.00E + 00* | HighMB |
| *UBE2C* | *0.00E + 00* | 1.522 | 0.591 | 0.035 | *0.00E + 00* | HighMB |
| *CENPF* | *0.00E + 00* | 1.499 | 0.631 | 0.068 | *0.00E + 00* | HighMB |
| *ATP5IF1* | *6.12E-252* | 1.436 | 0.923 | 0.483 | *1.61E-247* | HighMB |
| *CKS2* | *1.39E-296* | 1.432 | 0.831 | 0.310 | *3.66E-292* | HighMB |
| *GAPDH* | *0.00E + 00* | 1.426 | 0.996 | 0.955 | *0.00E + 00* | HighMB |
| *IGHE* | *0.00E + 00* | 1.405 | 0.451 | 0.026 | *0.00E + 00* | HighMB |
| *HSP90B1* | *6.94E-138* | 1.380 | 0.937 | 0.647 | *1.82E-133* | HighMB |
| *MZB1* | *7.90E-87* | 1.371 | 0.818 | 0.500 | *2.07E-82* | HighMB |
| *TOP2A* | *0.00E + 00* | 1.367 | 0.601 | 0.041 | *0.00E + 00* | HighMB |
| *PRDX2* | *9.32E-252* | 1.365 | 0.864 | 0.396 | *2.45E-247* | HighMB |
| *PRDX1* | *2.38E-260* | 1.358 | 0.977 | 0.738 | *6.25E-256* | HighMB |
| *CDC20* | *0.00E + 00* | 1.339 | 0.585 | 0.024 | *0.00E + 00* | HighMB |
| *ELOB* | *7.67E-301* | 1.337 | 0.968 | 0.537 | *2.01E-296* | HighMB |
| *ATP5MF* | *0.00E + 00* | 1.336 | 0.975 | 0.541 | *0.00E + 00* | HighMB |
| *TUBA1C* | *2.74E-244* | 1.334 | 0.755 | 072 | *7.18E-240* | HighMB |
| *FABP5* | *7.55E-229* | 1.330 | 0.847 | 0.370 | *1.98E-224* | HighMB |
| *KPNA2* | *0.00E + 00* | 1.321 | 0.788 | 029 | *0.00E + 00* | HighMB |
| *CALR* | *5.37E-178* | 1.318 | 0.949 | 0.727 | *1.41E-173* | HighMB |
| *PDIA4* | *2.54E-95* | 1.307 | 0.754 | 0.383 | *6.68E-91* | HighMB |
| *NUSAP1* | *0.00E + 00* | 1.306 | 0.706 | 0.104 | *0.00E + 00* | HighMB |
| *NDUFB3* | *1.07E-180* | 1.303 | 0.917 | 0.587 | *2.81E-176* | HighMB |
| *CCNB1* | *0.00E + 00* | 1.300 | 0.639 | 0.068 | *0.00E + 00* | HighMB |
| *MCM7* | *7.76E-271* | 149 | 0.743 | 013 | *2.04E-266* | HighMB |
| *GSTP1* | *1.56E-207* | 137 | 0.959 | 0.675 | *4.09E-203* | HighMB |
| *ATP5F1B* | *6.35E-261* | 136 | 0.956 | 0.530 | *1.67E-256* | HighMB |
| *ATF5* | *3.19E-191* | 123 | 0.794 | 0.349 | *8.38E-187* | HighMB |
| *PLK1* | *0.00E + 00* | 112 | 0.526 | 0.020 | *0.00E + 00* | HighMB |
| *MKI67* | *0.00E + 00* | 109 | 0.517 | 0.044 | *0.00E + 00* | HighMB |
| *COX5A* | *1.95E-289* | 1.199 | 0.972 | 0.702 | *5.11E-285* | HighMB |
| *ATP5PF* | *9.52E-289* | 1.199 | 0.952 | 0.508 | *2.50E-284* | HighMB |
| *ATP5MC1* | *1.37E-284* | 1.198 | 0.918 | 0.437 | *3.61E-280* | HighMB |
| *CHCHD2* | *0.00E + 00* | 1.198 | 0.986 | 0.894 | *0.00E + 00* | HighMB |
| *UBE2S* | *5.68E-200* | 1.197 | 0.682 | 047 | *1.49E-195* | HighMB |
| *SEM1* | *2.14E-263* | 1.179 | 0.959 | 0.511 | *5.62E-259* | HighMB |
| *C12orf75* | *1.81E-205* | 1.178 | 0.823 | 0.369 | *4.76E-201* | HighMB |
| *RAN* | *9.95E-261* | 1.159 | 0.968 | 0.794 | *2.61E-256* | HighMB |
| *HSPA8* | *1.81E-194* | 1.152 | 0.977 | 0.859 | *4.75E-190* | HighMB |
| *SMC4* | *1.05E-269* | 1.148 | 0.791 | 088 | *2.76E-265* | HighMB |
| *SNRPG* | *2.24E-250* | 1.144 | 0.958 | 0.703 | *5.87E-246* | HighMB |
| *NCL* | *2.17E-196* | 1.134 | 0.959 | 0.747 | *5.70E-192* | HighMB |
| *JPT1* | *2.61E-232* | 1.133 | 0.877 | 0.401 | *6.85E-228* | HighMB |
| *ATP5F1E* | *1.10E-247* | 1.130 | 0.987 | 0.579 | *2.90E-243* | HighMB |
| *ATP5ME* | *3.18E-241* | 1.127 | 0.931 | 0.493 | *8.34E-237* | HighMB |
| *TPI1* | *4.30E-227* | 1.119 | 0.975 | 0.755 | *1.13E-222* | HighMB |
| *CDK1* | *0.00E + 00* | 1.117 | 0.559 | 0.024 | *0.00E + 00* | HighMB |
| *RRM2* | *4.50E-290* | 1.116 | 0.401 | 0.022 | *1.18E-285* | HighMB |
| *CKS1B* | *0.00E + 00* | 1.115 | 0.787 | 027 | *0.00E + 00* | HighMB |
| *ATP5PB* | *1.75E-242* | 1.111 | 0.914 | 0.459 | *4.59E-238* | HighMB |
| *GSTM5* | *3.51E-240* | 1.105 | 0.306 | 0.010 | *9.22E-236* | HighMB |
| *DUT* | *1.56E-137* | 1.103 | 0.878 | 0.585 | *4.10E-133* | HighMB |
| *LDHB* | *3.84E-239* | 1.101 | 0.979 | 0.720 | *1.01E-234* | HighMB |
| *HMGA1* | *3.98E-210* | 1.099 | 0.967 | 0.701 | *1.05E-205* | HighMB |
| *ATP1A1* | *4.50E-187* | 1.097 | 0.708 | 070 | *1.18E-182* | HighMB |
| *PKM* | *6.58E-230* | 1.091 | 0.976 | 0.707 | *1.73E-225* | HighMB |
| *HIST1H1B* | *1.76E-200* | 1.083 | 0.399 | 0.056 | *4.62E-196* | HighMB |
| *DTYMK* | *0.00E + 00* | 1.082 | 0.793 | 006 | *0.00E + 00* | HighMB |
| *HNRNPAB* | *5.79E-246* | 1.079 | 0.842 | 0.344 | *1.52E-241* | HighMB |
| *XBP1* | *2.82E-103* | 1.079 | 0.745 | 0.379 | *7.39E-99* | HighMB |
| *CCT5* | *1.25E-232* | 1.071 | 0.909 | 0.471 | *3.28E-228* | HighMB |
| *IGKV1-39* | *1.05E-282* | 1.070 | 0.452 | 0.039 | *2.76E-278* | HighMB |
| *CCNB2* | *0.00E + 00* | 1.068 | 0.569 | 0.032 | *0.00E + 00* | HighMB |
| *PPIA* | *1.65E-276* | 1.066 | 0.994 | 0.971 | *4.33E-272* | HighMB |
| *ATP5MG* | *1.04E-253* | 1.062 | 0.981 | 0.566 | *2.73E-249* | HighMB |
| *COX7A2* | *1.25E-164* | 1.052 | 0.950 | 0.741 | *3.29E-160* | HighMB |
| *PCNA* | *3.16E-211* | 1.049 | 0.644 | 0.193 | *8.29E-207* | HighMB |
| *SNRPD1* | *3.52E-222* | 1.045 | 0.926 | 0.605 | *9.24E-218* | HighMB |
| *TPX2* | *0.00E + 00* | 1.044 | 0.613 | 0.045 | *0.00E + 00* | HighMB |
| *CCT7* | *6.91E-214* | 1.040 | 0.916 | 0.537 | *1.81E-209* | HighMB |
| *MAD2L1* | *0.00E + 00* | 1.035 | 0.727 | 0.119 | *0.00E + 00* | HighMB |
| *DEK* | *3.02E-147* | 1.032 | 0.871 | 0.609 | *7.93E-143* | HighMB |
| *GSTM2* | *1.05E-267* | 1.028 | 0.414 | 0.034 | *2.77E-263* | HighMB |
| *PRDX3* | *4.18E-189* | 1.028 | 0.898 | 0.517 | *1.10E-184* | HighMB |
| *FEN1* | *0.00E + 00* | 1.025 | 0.695 | 0.129 | *0.00E + 00* | HighMB |
| *HSP90AA1* | *6.71E-177* | 1.016 | 0.969 | 0.757 | *1.76E-172* | HighMB |
| *MINOS1* | *5.80E-111* | 1.014 | 0.513 | 011 | *1.52E-106* | HighMB |
| *HMGB1* | *8.85E-177* | 1.011 | 0.978 | 0.924 | *2.32E-172* | HighMB |
| *SRP14* | *5.96E-113* | 1.011 | 0.968 | 0.908 | *1.56E-108* | HighMB |
| *ZWINT* | *0.00E + 00* | 1.009 | 0.636 | 0.044 | *0.00E + 00* | HighMB |
| *IGKV4-1* | *1.74E-41* | 1.007 | 092 | 0.114 | *4.56E-37* | HighMB |
| *NDUFB11* | *3.52E-207* | 1.005 | 0.966 | 0.734 | *9.25E-203* | HighMB |
| *IGHA1* | *2.15E-10* | 1.003 | 0.313 | 0.409 | *5.66E-06* | HighMB |

*Abbreviation: MB: malignant B cells.*

**Table S6.** Differential genes identified in macrophage and monocytes subgroups (*p* < 0.05, avg_log2FC > 1).

| **gene** | **p_val** | **avg_log2FC** | **pct.1** | **pct.2** | **p_val_adj** | **Celltype** |
| --- | --- | --- | --- | --- | --- | --- |
| *FCN1* | 1.19E-28 | 1.351 | 0.774 | 0.331 | 2.65E-24 | Mono |
| *S100A9* | 1.77E-13 | 1.345 | 0.779 | 0.531 | 3.97E-09 | Mono |
| *S100A8* | 2.87E-12 | 1.328 | 0.659 | 0.403 | 6.42E-08 | Mono |
| *S100A4* | 6.48E-09 | 1.149 | 0.581 | 0.411 | 1.45E-04 | Mono |
| *APOBEC3A* | 3.52E-07 | 1.059 | 0.313 | 0.140 | 7.87E-03 | Mono |
| *MS4A7* | 1.50E-18 | 1.045 | 0.659 | 0.300 | 3.36E-14 | Mono |
| *LTB* | 1.10E-06 | 1.534 | 0.346 | 0.173 | 2.46E-02 | DC_1 |
| *CLEC10A* | 5.13E-24 | 1.516 | 0.805 | 0.523 | 1.15E-19 | DC_1 |
| *CD1C* | 1.99E-37 | 1.410 | 0.564 | 0.078 | 4.45E-33 | DC_1 |
| *JAML* | 2.55E-32 | 1.178 | 0.955 | 0.571 | 5.70E-28 | DC_1 |
| *CD1E* | 8.38E-21 | 1.080 | 0.451 | 0.094 | 1.88E-16 | DC_1 |
| *GSN* | 7.51E-27 | 1.022 | 0.985 | 0.836 | 1.68E-22 | DC_1 |
| *S100B* | 3.06E-05 | 1.010 | 0.391 | 0.214 | 6.85E-01 | DC_1 |
| *PTGDS* | 2.16E-40 | 4.036 | 0.459 | 0.024 | 4.84E-36 | LA_TAM |
| *CCL18* | 8.21E-37 | 2.616 | 0.414 | 0.020 | 1.84E-32 | LA_TAM |
| *APOE* | 7.40E-24 | 2.568 | 0.703 | 0.274 | 1.66E-19 | LA_TAM |
| *CHI3L1* | 6.85E-12 | 2.351 | 0.468 | 0.175 | 1.53E-07 | LA_TAM |
| *CTSD* | 9.51E-11 | 2.132 | 0.748 | 0.596 | 2.13E-06 | LA_TAM |
| *GPNMB* | 4.41E-23 | 2.006 | 0.495 | 0.116 | 9.87E-19 | LA_TAM |
| *APOC1* | 9.89E-18 | 1.860 | 0.784 | 0.436 | 2.21E-13 | LA_TAM |
| *PLA2G2D* | 4.02E-34 | 1.832 | 0.477 | 0.050 | 9.01E-30 | LA_TAM |
| *CAPG* | 3.63E-10 | 1.602 | 0.694 | 0.550 | 8.13E-06 | LA_TAM |
| *MMP9* | 2.80E-12 | 1.593 | 0.595 | 0.298 | 6.28E-08 | LA_TAM |
| *NUPR1* | 6.37E-28 | 1.411 | 0.324 | 0.018 | 1.43E-23 | LA_TAM |
| *CTSL* | 7.24E-08 | 1.362 | 0.568 | 0.404 | 1.62E-03 | LA_TAM |
| *RARRES1* | 2.44E-23 | 1.170 | 0.396 | 0.057 | 5.47E-19 | LA_TAM |
| *IL32* | 2.95E-30 | 1.099 | 0.775 | 0.200 | 6.60E-26 | LA_TAM |
| *FUCA1* | 3.21E-11 | 1.097 | 0.505 | 0.243 | 7.18E-07 | LA_TAM |
| *LGMN* | 3.55E-08 | 1.069 | 0.577 | 0.375 | 7.94E-04 | LA_TAM |
| *FTL* | 1.53E-12 | 1.001 | 0.991 | 1.000 | 3.43E-08 | LA_TAM |
| *MT1H* | 1.04E-30 | 3.142 | 0.677 | 0.114 | 2.33E-26 | IFN_TAM |
| *MT1G* | 4.31E-31 | 2.994 | 0.785 | 0.169 | 9.64E-27 | IFN_TAM |
| *CCL8* | 2.32E-23 | 2.491 | 0.508 | 0.080 | 5.20E-19 | IFN_TAM |
| *CCL2* | 1.20E-27 | 2.487 | 0.754 | 0.179 | 2.69E-23 | IFN_TAM |
| *MT1X* | 1.87E-25 | 2.306 | 0.862 | 0.329 | 4.20E-21 | IFN_TAM |
| *MT2A* | 4.70E-24 | 2.167 | 1.000 | 0.717 | 1.05E-19 | IFN_TAM |
| *CXCL10* | 1.74E-15 | 2.147 | 0.846 | 0.442 | 3.90E-11 | IFN_TAM |
| *SLC39A8* | 7.25E-32 | 2.130 | 0.908 | 0.339 | 1.62E-27 | IFN_TAM |
| *MT1F* | 1.03E-30 | 1.944 | 0.815 | 0.197 | 2.31E-26 | IFN_TAM |
| *MT1E* | 3.38E-29 | 1.855 | 0.877 | 0.273 | 7.57E-25 | IFN_TAM |
| *CXCL9* | 1.28E-19 | 1.830 | 0.969 | 0.586 | 2.86E-15 | IFN_TAM |
| *MT1M* | 7.35E-28 | 1.783 | 0.569 | 0.084 | 1.64E-23 | IFN_TAM |
| *CCL4* | 7.63E-09 | 1.699 | 0.523 | 0.217 | 1.71E-04 | IFN_TAM |
| *CD14* | 6.59E-23 | 1.670 | 0.969 | 0.647 | 1.48E-18 | IFN_TAM |
| *C1QC* | 6.65E-18 | 1.534 | 0.938 | 0.586 | 1.49E-13 | IFN_TAM |
| *C1QA* | 2.71E-19 | 1.528 | 0.969 | 0.622 | 6.06E-15 | IFN_TAM |
| *SOD2* | 3.30E-23 | 1.500 | 1.000 | 0.761 | 7.39E-19 | IFN_TAM |
| *SNX10* | 6.68E-21 | 1.488 | 0.938 | 0.657 | 1.49E-16 | IFN_TAM |
| *C1QB* | 9.38E-16 | 1.383 | 0.923 | 0.659 | 2.10E-11 | IFN_TAM |
| *MARCKS* | 4.25E-21 | 1.327 | 0.969 | 0.554 | 9.52E-17 | IFN_TAM |
| *FCGR1A* | 2.89E-23 | 1.315 | 0.985 | 0.612 | 6.46E-19 | IFN_TAM |
| *DRAM1* | 1.92E-25 | 1.291 | 0.954 | 0.462 | 4.30E-21 | IFN_TAM |
| *C2* | 2.37E-23 | 1.270 | 0.815 | 0.249 | 5.30E-19 | IFN_TAM |
| *IL4I1* | 3.16E-20 | 1.258 | 1.000 | 0.590 | 7.07E-16 | IFN_TAM |
| *CD163* | 5.23E-27 | 1.258 | 0.938 | 0.307 | 1.17E-22 | IFN_TAM |
| *MAFB* | 4.12E-21 | 1.245 | 1.000 | 0.500 | 9.22E-17 | IFN_TAM |
| *GBP1* | 1.69E-17 | 1.243 | 0.969 | 0.783 | 3.77E-13 | IFN_TAM |
| *CCL3* | 3.17E-12 | 1.236 | 0.800 | 0.388 | 7.10E-08 | IFN_TAM |
| *GPR84* | 6.81E-26 | 1.233 | 0.815 | 0.253 | 1.52E-21 | IFN_TAM |
| *VSIG4* | 1.08E-22 | 1.202 | 0.738 | 0.209 | 2.43E-18 | IFN_TAM |
| *NINJ1* | 2.11E-22 | 1.200 | 1.000 | 0.588 | 4.72E-18 | IFN_TAM |
| *IL18* | 5.83E-15 | 1.192 | 0.877 | 0.532 | 1.30E-10 | IFN_TAM |
| *TMEM176B* | 2.75E-23 | 1.187 | 1.000 | 0.759 | 6.15E-19 | IFN_TAM |
| *FCER1G* | 3.01E-25 | 1.180 | 1.000 | 0.924 | 6.73E-21 | IFN_TAM |
| *CALHM6* | 2.41E-19 | 1.177 | 1.000 | 0.876 | 5.40E-15 | IFN_TAM |
| *SERPING1* | 5.49E-22 | 1.172 | 0.985 | 0.592 | 1.23E-17 | IFN_TAM |
| *GLUL* | 7.09E-16 | 1.155 | 1.000 | 0.667 | 1.59E-11 | IFN_TAM |
| *NR1H3* | 3.90E-20 | 1.153 | 1.000 | 0.530 | 8.74E-16 | IFN_TAM |
| *CLEC4E* | 3.81E-23 | 1.152 | 0.954 | 0.448 | 8.54E-19 | IFN_TAM |
| *PLEK* | 1.01E-18 | 1.150 | 1.000 | 0.888 | 2.26E-14 | IFN_TAM |
| *CD68* | 2.22E-21 | 1.146 | 1.000 | 0.865 | 4.98E-17 | IFN_TAM |
| *MAP1LC3A* | 1.83E-24 | 1.130 | 0.646 | 0.131 | 4.09E-20 | IFN_TAM |
| *ANKRD22* | 1.20E-22 | 1.124 | 0.985 | 0.544 | 2.68E-18 | IFN_TAM |
| *CTSB* | 1.14E-17 | 1.118 | 1.000 | 0.845 | 2.55E-13 | IFN_TAM |
| *ACP2* | 3.56E-23 | 1.079 | 0.985 | 0.486 | 7.96E-19 | IFN_TAM |
| *TMEM176A* | 5.09E-20 | 1.071 | 1.000 | 0.717 | 1.14E-15 | IFN_TAM |
| *MARCO* | 7.81E-06 | 1.065 | 0.446 | 0.219 | 1.75E-01 | IFN_TAM |
| *FCGR3A* | 2.72E-17 | 1.050 | 0.954 | 0.484 | 6.09E-13 | IFN_TAM |
| *TNFSF13B* | 3.92E-18 | 1.048 | 1.000 | 0.843 | 8.78E-14 | IFN_TAM |
| *PRDX1* | 1.00E-16 | 1.045 | 1.000 | 0.789 | 2.25E-12 | IFN_TAM |
| *CFB* | 3.67E-27 | 1.029 | 0.738 | 0.175 | 8.22E-23 | IFN_TAM |
| *CD38* | 2.54E-18 | 1.023 | 0.862 | 0.418 | 5.70E-14 | IFN_TAM |
| *GBP5* | 5.27E-13 | 1.004 | 0.908 | 0.506 | 1.18E-08 | IFN_TAM |
| *IL32* | 5.40E-09 | 1.001 | 0.585 | 0.277 | 1.21E-04 | IFN_TAM |
| *CD44* | 3.50E-19 | 1.001 | 0.954 | 0.725 | 7.84E-15 | IFN_TAM |
| *DNASE1L3* | 7.66E-44 | 2.874 | 0.780 | 0.076 | 1.72E-39 | DC_2 |
| *RGCC* | 6.09E-59 | 2.524 | 0.756 | 0.036 | 1.36E-54 | DC_2 |
| *CST3* | 1.64E-13 | 2.482 | 1.000 | 0.981 | 3.66E-09 | DC_2 |
| *CLEC9A* | 4.16E-82 | 2.256 | 0.732 | 0.006 | 9.32E-78 | DC_2 |
| *SNX3* | 2.71E-22 | 2.245 | 0.976 | 0.806 | 6.07E-18 | DC_2 |
| *IRF8* | 4.00E-22 | 2.195 | 0.878 | 0.344 | 8.95E-18 | DC_2 |
| *CCND1* | 1.74E-36 | 2.089 | 0.878 | 0.150 | 3.88E-32 | DC_2 |
| *LSP1* | 2.86E-23 | 2.043 | 1.000 | 0.696 | 6.41E-19 | DC_2 |
| *CPNE3* | 1.54E-27 | 2.004 | 0.780 | 0.169 | 3.44E-23 | DC_2 |
| *CPVL* | 2.45E-12 | 1.984 | 0.976 | 0.869 | 5.48E-08 | DC_2 |
| *C1orf54* | 2.20E-11 | 1.778 | 0.780 | 0.523 | 4.92E-07 | DC_2 |
| *NAPSA* | 9.50E-31 | 1.574 | 0.854 | 0.148 | 2.13E-26 | DC_2 |
| *ID2* | 3.99E-13 | 1.507 | 0.634 | 0.198 | 8.93E-09 | DC_2 |
| *NAAA* | 3.61E-11 | 1.505 | 0.854 | 0.555 | 8.08E-07 | DC_2 |
| *ANPEP* | 1.46E-23 | 1.478 | 0.683 | 0.129 | 3.26E-19 | DC_2 |
| *IDO1* | 1.72E-13 | 1.413 | 0.951 | 0.466 | 3.85E-09 | DC_2 |
| *XCR1* | 8.17E-78 | 1.411 | 0.634 | 0.000 | 1.83E-73 | DC_2 |
| *S100A10* | 1.92E-12 | 1.305 | 0.951 | 0.837 | 4.29E-08 | DC_2 |
| *HLA-DOB* | 4.91E-41 | 1.270 | 0.683 | 0.053 | 1.10E-36 | DC_2 |
| *RAB7B* | 1.31E-51 | 1.256 | 0.61 | 0.021 | 2.93E-47 | DC_2 |
| *WDFY4* | 3.30E-19 | 1.246 | 0.61 | 0.129 | 7.39E-15 | DC_2 |
| *MARCKSL1* | 2.28E-16 | 1.239 | 0.927 | 0.352 | 5.10E-12 | DC_2 |
| *NET1* | 5.06E-43 | 1.211 | 0.585 | 0.029 | 1.13E-38 | DC_2 |
| *CYB5R3* | 3.54E-19 | 1.191 | 0.854 | 0.344 | 7.92E-15 | DC_2 |
| *TSPAN13* | 4.40E-51 | 1.188 | 0.634 | 0.023 | 9.85E-47 | DC_2 |
| *ASB2* | 5.24E-54 | 1.134 | 0.561 | 0.011 | 1.17E-49 | DC_2 |
| *RGS10* | 2.56E-15 | 1.128 | 1 | 0.797 | 5.73E-11 | DC_2 |
| *GSTP1* | 4.98E-16 | 1.113 | 1 | 0.97 | 1.11E-11 | DC_2 |
| *TAGLN2* | 1.85E-15 | 1.100 | 1 | 0.926 | 4.15E-11 | DC_2 |
| *CD74* | 1.68E-13 | 1.093 | 1 | 1 | 3.75E-09 | DC_2 |
| *HLA-DQB1* | 1.72E-13 | 1.092 | 1 | 0.954 | 3.86E-09 | DC_2 |
| *CADM1* | 9.15E-49 | 1.088 | 0.537 | 0.013 | 2.05E-44 | DC_2 |
| *STMN1* | 5.18E-08 | 1.086 | 0.293 | 0.061 | 1.16E-03 | DC_2 |
| *SELPLG* | 2.05E-17 | 1.071 | 0.78 | 0.253 | 4.60E-13 | DC_2 |
| *LMNA* | 4.18E-15 | 1.067 | 0.707 | 0.226 | 9.35E-11 | DC_2 |
| *TAP1* | 6.74E-16 | 1.066 | 1 | 0.677 | 1.51E-11 | DC_2 |
| *CST7* | 1.85E-20 | 1.054 | 0.634 | 0.112 | 4.14E-16 | DC_2 |
| *CKLF* | 5.32E-12 | 1.053 | 0.805 | 0.39 | 1.19E-07 | DC_2 |
| *PPT1* | 8.96E-08 | 1.050 | 0.927 | 0.741 | 2.01E-03 | DC_2 |
| *DBN1* | 5.45E-66 | 1.046 | 0.585 | 0.004 | 1.22E-61 | DC_2 |
| *MPEG1* | 3.69E-05 | 1.029 | 0.683 | 0.549 | 8.26E-01 | DC_2 |
| *ACTG1* | 1.76E-17 | 1.017 | 1 | 1 | 3.93E-13 | DC_2 |
| *CSRP1* | 5.02E-18 | 1.009 | 0.756 | 0.219 | 1.12E-13 | DC_2 |
| *PTMS* | 1.27E-13 | 1.001 | 0.927 | 0.468 | 2.84E-09 | DC_2 |

*Abbreviation: DC: dendritic cell; IFN_TAMs: interferon-primed tumor-associated macrophages; LA_TAMs: lipid-associated tumor-associated macrophages.*

**Table S7.** Markers for 30 cell types used in ssGSEA.

| **Cell Types** | **Markers** |
| --- | --- |
| Activated B | *"ADAM28";"CD180";"CD79B";"BLK";"CD19";"MS4A1";"TNFRSF17";"IGHM";"GNG7";"MICAL3";"SPIB";"HLA-DOB";"IGKC";"PNOC";"FCRL2";"BACH2";"CR2";"TCL1A";"AKNA";"ARHGAP25";"CCL21";"CD27";"CD38";"CLEC17A";"CLEC9A";"CLECL1"* |
| Activated CD4 T | *"AIM2";"BIRC3";"BRIP1";"CCL20";"CCL4";"CCL5";"CCNB1";"CCR7";"DUSP2";"ESCO2";"ETS1";"EXO1";"EXOC6";"IARS";"ITK";"KIF11";"KNTC1";"NUF2";"PRC1";"PSAT1";"RGS1";"RTKN2";"SAMSN1";"SELL";"TRAT1"* |
| Activated CD8 T | *"ADRM1";"AHSA1";"C1GALT1C1";"CCT6B";"CD37";"CD3D";"CD3E";"CD3G";"CD69";"CD8A";"CETN3";"CSE1L";"GEMIN6";"GNLY";"GPT2";"GZMA";"GZMH";"GZMK";"IL2RB";"LCK";"MPZL1";"NKG7";"PIK3IP1";"PTRH2";"TIMM13";"ZAP70"* |
| Central memory CD4 T | *"ABHD3";"AHNAK";"ANXA2P2";"AQP3";"ATHL1";"BMI1";"BZW2";"CD63";"COL4A1";"CYLD";"ELMO2";"FYN";"GLIPR1";"GSS";"IFITM2";"ITGB1";"ITGB2";"KLF5";"LSP1";"NDUFB9";"PKM2";"SFXN3";"SIRPG";"SMAD4";"STX4";"TRADD";"VIM";"XRCC6"* |
| Central memory CD8 T | *"ACTN4";"ADAM12";"ADCY9";"F13A1";"FCER1G";"FCGR3B";"FGF7";"FKBP4";"GLUD1";"GM2A";"GUSB";"IL1RN";"NOL11";"NTRK1";"RARA";"RNF128";"SIGLEC1";"TNFRSF11A";"TOX4";"UBA52";"ULBP1"* |
| Effector memeory CD4 T | *"ATM";"CASP3";"CASQ1";"CD300E";"DARS";"DOCK9";"EXOSC9";"EZH2";"GDE1";"IL34";"NCOA4";"NEFL";"PDGFRL";"PTGS1";"REPS1";"SCG2";"SDPR";"SIGLEC14";"SIGLEC6";"TAL1";"TFEC";"TIPIN";"TPK1";"UQCRB";"USP9Y";"WIPF1";"ZCRB1"* |
| Effector memeory CD8 T | *"ACAP1";"APOL3";"ARHGAP10";"ATP10D";"C3AR1";"CCR5";"CD160";"CD55";"CFLAR";"CMKLR1";"DAPP1";"FCRL6";"FLT3LG";"GZMM";"HAPLN3";"HLA-DMB";"HLA-DPA1";"HLADPB1";"IFI16";"LIME1";"LTK";"NFKBIA";"SETD7";"SIK1";"TRIB2"* |
| Gamma delta T | *"ACP5";"AQP9";"BTN3A2";"C1orf54";"CARD8";"CCL18";"CD209";"CD33";"CD36";"CDK5";"IL10RB";"KLRF1";"LGALS1";"MAPK7";"KLHL7";"KRT80";"LAMC1";"LCORL";"LMNB1";"MEIS3P1";"MPL";"FABP1";"FABP5";"FADD";"MFAP3L";"MINPP1";"RPS24";"RPS7";"RPS9";"DBNL";"CCL13"* |
| Immature B | *"CD22";"CYBB";"FAM129C";"FCRL1";"FCRL3";"FCRL5";"FCRLA";"HDAC9";"HLADQA1";"HVCN1";"KIAA0226";"NCF1";"NCF1B";"P2RY10";"SP100";"TXNIP";"STAP1";"TAGAP";"ZCCHC2"* |
| Memory B | *"AICDA";"CCNA2";"CDKN3";"CLCN5";"ENPP1";"FCER1A";"FCRL4";"MYC";"RUNX2";"SORL1";"SOX5";"STAT5A";"STAT5B";"TLR9"* |
| Regulatory T | *"CCL3L1";"CD72";"CLEC5A";"FOXP3";"ITGA4";"L1CAM";"LIPA";"LRP1";"LRRC42";"MARCO";"MMP12";"MNDA";"MRC1";"MS4A6A";"PELO";"PLEK";"PRSS23";"PTGIR";"ST8SIA4";"STAB1"* |
| T follicular helper | *"B3GAT1";"CDK5R1";"PDCD1";"BCL6";"CD200";"CD83";"CD84";"FGF2";"GPR18";"CEBPA";"CECR1";"CLEC10A";"CLEC4A";"CSF1R";"CTSS";"DMN";"DPP4";"LRRC32";"MC5R";"MICA";"NCAM1";"NCR2";"NRP1";"PD-L2";"PDCD6";"PRDX1";"RAE1";"RAET1E";"SIGLEC7";"SIGLEC9";"TYRO3";"CHST12";"CLIC3";"IVNS1ABP";"KIR2DL2";"LGMN"* |
| Type 1 T helper | *"CD70";"TBX21";"ADAM8";"AHCYL2";"ALCAM";"B3GALNT1";"BBS12";"BST1";"CD151";"CD47";"CD48";"CD52";"CD53";"CD59";"CD6";"CD68";"CD7";"CD96";"CFHR3";"CHRM3";"CLEC7A";"COL23A1";"COL4A4";"COL5A3";"DAB1";"DLEU7";"DOC2B";"EMP1";"F12";"FURIN";"GAB3";"GATM";"GFPT2";"GPR25";"GREM2";"HAVCR1";"HSD11B1";"HUNK";"IGF2";"RCSD1";"RYR1";"SAV1";"SELE";"SELP";"SH3KBP1";"SIT1";"SLC35B3";"SIGLEC10";"SKAP1";"THUMPD2";"TIGIT";"ZEB2";"ENC1";"FAM134B";"FBXO30";"FCGR2C";"STAC";"LTC4S";"MAN1B1";"MDH1";"MMD";"RGS16";"IL12A";"P2RX5";"CD97";"ITGB4";"ICAM3";"METRNL";"TNFRSF1A";"IRF1";"HTR2B";"CALD1";"MOCOS";"TRAF3IP2";"TLR8";"TRAF1";"DUSP14"* |
| Type 17 T helper | *"IL17A";"IL17RA";"C2CD4A";"C2CD4B";"CA2";"CCDC65";"CEACAM3";"IL17C";"IL17F";"IL17RC";"IL17RE";"IL23A";"ILDR1";"LONRF3";"SH2D6";"TNIP2";"ABCA1";"ABCB1";"ADAMTS12";"ANK1";"ANKRD22";"B3GALT2";"CAMTA1";"CCR9";"CD40";"GPR44";"IFT80"* |
| Type 2 T helper | *"ASB2";"CSRP2";"DAPK1";"DLC1";"DNAJC12";"DUSP6";"GNAI1";"LAMP3";"NRP2";"OSBPL1A";"PDE4B";"PHLDA1";"PLA2G4A";"RAB27B";"RBMS3";"RNF125";"TMPRSS3";"GATA3";"BIRC5";"CDC25C";"CDC7";"CENPF";"CXCR6";"DHFR";"EVI5";"GSTA4";"HELLS";"IL26";"LAIR2"* |
| Activated dendritic | *"ABCD1";"C1QC";"CAPG";"CCL3L3";"CD207";"CD302";"ATP5B";"ATP5L";"ATP6V1A";"BCL2L1";"C1QB";"SNURF";"SPCS3";"CCNA1";"CEACAM8";"NOS2";"SRA1";"TNFRSF6B";"TREM1";"TREML1";"RHOA";"SLC25A37";"TNFSF14";"TREML4";"VNN2";"XPO6";"CLEC4C";"TNFAIP2";"UBD";"ACTR3";"RAB1A";"SLA";"HLA-DQA2";"SIGLEC5";"SLAMF9"* |
| CD56bright natural killer | *"ABAT";"C11orf75";"C5orf15";"CDHR1";"DCAF12";"DYNLL1";"GPR137B";"HCP5";"HDGFRP2";"KRT86";"MLST8";"ELMOD3";"ENTPD5";"FAM119A";"FAM179A";"CLIC2";"COX7A2L";"CREB3L4";"CSF1";"CSNK2A2";"CSTA";"CSTB";"CTPS";"CTSD";"FST";"GATA2";"GMPR";"HDC";"HEY1";"HOXA1";"HS2ST1";"HS3ST1";"BCL11B";"CDH3";"MYL6B";"NAA16";"ClQA";"ClQB";"CYP27B1";"EIF3M"* |
| CD56dim natural killer | *"CYP27A1";"DDX55";"DYRK2";"RPL37A";"NOTCH3";"AKR7A3";"GPRC5C";"GRIN1";"HLAE";"PORCN";"PSMC4";"UPP1";"IL21R";"KIR2DS1";"KIR2DS2";"KIR2DS5"* |
| Eosinophil | *"GIPR";"KRT18P50";"LRMP";"FOSB";"RRP12";"GPR183";"NR4A3";"ST3GAL6";"DEPDC5";"PDE6C";"PKD2L2";"GPR65";"IL5RA";"P2RY14";"DACH1";"DAPK2";"EMR3"* |
| Immature dendritic | *"ACADM";"AHCYL1";"ALDH1A2";"ALDH3A2";"ALDH9A1";"ALOX15";"AMT";"ARL1";"ATIC";"ATP5A1";"CAPZA1";"LILRA5";"RDX";"RRAGD";"TACSTD2";"INPP5F";"RAB38";"PLAU";"CSF3R";"SLC18A2";"AMPD2";"CLTB";"C1orf162"* |
| Macrophage | *"AIF1";"CCL1";"CCL14";"CCL23";"CCL26";"CD300LB";"CNR1";"CNR2";"EIF1";"EIF4A1";"FPR1";"FPR2";"FRAT2";"GPR27";"GPR77";"RNASE2";"MS4A2";"BASP1";"IGSF6";"HK3";"VNN1";"FES";"NPL";"FZD2";"FAM198B";"HNMT";"SLC15A3";"CD4";"TXNDC3";"FRMD4A";"CRYBB1";"HRH1";"WNT5B"* |
| Mast | *"ADAMTS3";"CPA3";"CMA1";"CTSG";"ARHGAP15";"CPM";"FCN1";"FTL";"HSPA6";"ITGA9";"RNASE3";"S100A4";"SIGLEC8";"SLC6A4";"PTGS2";"EGR3";"PILRA"* |
| MDSC | *"CCR2";"CD14";"CD2";"CD86";"CXCR4";"FCGR2A";"FCGR2B";"FCGR3A";"FERMT3";"GPSM3";"IL18BP";"IL4R";"ITGAL";"ITGAM";"PARVG";"PSAP";"PTGER2";"PTGES2";"S100A8";"S100A9"* |
| Monocyte | *"ASGR2";"CFP";"ASGR1";"CD1D";"UPK3A";"ACTG1";"ANXA5";"ATP6V1B2";"CFL1";"DAZAP2";"CTBS";"EMR4P";"HIVEP2";"MARCKSL1";"MBP";"MMP15";"PNPLA6";"TMBIM6";"PQBP1";"TEX264";"IKZF1"* |
| Natural killer | *"AKT3";"AXL";"BST2";"CDH2";"CRTAM";"CSF2RA";"CTSZ";"CXCL1";"CYTH1";"DAXX";"DGKH";"DLL4";"DPYD";"ERBB3";"F11R";"FAM27A";"FAM49A";"FASLG";"FCGR1A";"FN1";"FSTL1";"FUCA1";"GBP3";"GLS2";"GRB2";"LST1";"BCL2";"CDC5L";"FGF18";"FUT5";"FZR1";"GAGE2";"IGFBP5";"KANK2";"LDB3"* |
| Natural killer T | *"BTN2A2";"CD101";"CD109";"CNPY3";"CNPY4";"CREB1";"CRTC2";"CRTC3";"CSF2";"KLRC1";"FUT4";"ICAM2";"IL32";"LAMP2";"LILRB5";"KLRG1";"HSPA4";"HSPB6";"ISM2";"ITIH2";"KDM4C";"KIR2DS4";"KIRREL3";"SDCBP";"NFATC2IP";"MICB";"KIR2DL1";"KIR2DL3";"KIR3DL1";"KIR3DL2";"NCR1";"FOSL1";"TSLP";"SLC7A7";"SPP1";"TREM2";"UBASH3A";"YBX2";"CCDC88A";"CLEC1A";"THBD";"PDPN";"VCAM1";"EMR1"* |
| Neutrophil | *"CREB5";"CDA";"CHST15";"S100A12";"APOBEC3A";"CASP5";"MMP25";"HAL";"C1orf183";"FFAR2";"MAK";"CXCR1";"STEAP4";"MGAM";"BTNL8";"CXCR2";"TNFRSF10C";"VNN3"* |
| Plasmacytoid dendritic | *"CBX6";"DAB2";"DDX17";"HIGD1A";"IDH3A";"IL3RA";"MAGED1";"NUCB2";"OFD1";"OGT";"PDIA4";"SERTAD2";"SIRPA";"TMED2";"ENG";"FCAR";"IGF1";"ITGA2B";"GABARAP";"GPX1";"KRT23";"PROK2";"RALB";"RETNLB";"RNF141";"SEC14L1";"SEPX1";"EMP3";"CD300LF";"ABTB1";"KLHL21";"PHRF1"* |
| IFN_TAM | *"MT1H";"MT1G2";"CCL8";"CCL2";"MT1X";"MT2A";"CXCL10";"SLC39A8";"MT1F";"MT1E";"CD163";"CD68";"PD-L1"* |
| LA_TAM | *"PTGDS";"CCL18";"APOE";"CHI3L1";"CTSD";"GPNMB";"APOC1";"PLA2G2D";"CAPG";"MMP9";"ACP5";"CD68"* |

*Abbreviation: ssGSEA: Single-sample gene set enrichment analysis.*

**Table S8.** Quality control of 10 DLBCL samples in ST.

| **ID** | **Spots** | **Median Genes /Spot** | **Reads** | **Valid Barcodes** | **Valid UMIs** | **Sequencing Saturation** | **Q30 Bases in Barcode** | **Q30 Bases in Probe Read** | **Q30 Bases in UMI** |
| --- | --- | --- | --- | --- | --- | --- | --- | --- | --- |
| S1 | 4978 | 5219 | 324159529 | 0.99 | 1 | 0.78 | 0.97 | 0.98 | 0.97 |
| S2 | 4866 | 5954 | 331380362 | 0.99 | 1 | 0.759 | 0.97 | 0.97 | 0.97 |
| S3 | 4906 | 7343 | 331356071 | 0.99 | 1 | 0.53 | 0.97 | 0.97 | 0.96 |
| S4 | 4486 | 5200 | 325219600 | 0.99 | 1 | 0.81 | 0.97 | 0.97 | 0.97 |
| S5 | 4909 | 6865 | 328342295 | 0.99 | 1 | 0.6 | 0.97 | 0.97 | 0.96 |
| S6 | 4955 | 5941 | 374291928 | 0.98 | 1 | 0.75 | 0.98 | 0.97 | 0.97 |
| S7 | 4616 | 4678 | 326430429 | 0.99 | 1 | 0.86 | 0.97 | 0.97 | 0.97 |
| S8 | 4992 | 7237 | 321757368 | 0.99 | 1 | 0.64 | 0.97 | 0.98 | 0.97 |
| S9 | 4684 | 4584 | 334186091 | 0.99 | 1 | 0.86 | 0.98 | 0.97 | 0.97 |
| S10 | 4275 | 6939 | 391465547 | 0.98 | 1 | 0.75 | 0.98 | 0.97 | 0.97 |

*Abbreviation: DLBCL: diffuse large B-cell lymphoma; ST: spatial transcriptomics; UMI: unique molecular indentifier.*

**Table S9.** Annotation of 9 cell type markers for 10 DLBCL samples in ST.

| **CellType** | **Markers** | **Spots** |
| --- | --- | --- |
| B cell | *"PAX5","MS4A1","CD79A","CD79B"* | 22363 |
| NKT cell | *"PNN","DMTF1","CARD11","PMAIP1"* | 6619 |
| Fibroblast | *"COL1A2","COL1A1","COL3A1","MMP2","DCN"* | 7014 |
| Macrophage | *"CXCL9","LYZ","CD68","CXCL10","CCL18"* | 2864 |
| T cell | *"TRAC","CD3D","CD3E","CD3G"* | 2498 |
| Neutrophil | *"CXCL8","G0S2","CXCL5","SPP1","PTGS2"* | 2251 |
| Plasma cell | *"JCHAIN","IGHG1","XBP1","IGHA1","IGKC"* | 1833 |
| Muscle cell | *"MYH11","TAGLN","CSRP1"* | 1825 |
| Endotheliocyte | *"VWF","A2M","TPM2","PECAM1"* | 400 |

*Abbreviation: DLBCL: diffuse large B-cell lymphoma; ST: spatial transcriptomics.*

**Table S10.** Number of spots of 9 cell types in each samples.

| **ID/Spots** | **B cell** | **NKT cell** | **Fibroblast** | **Macrophage** | **T cell** | **Neutrophil** | **Muscle cell** | **Plasma cell** | **Endotheliocyte** |
| --- | --- | --- | --- | --- | --- | --- | --- | --- | --- |
| S1 | 2438 | 894 | 738 | 146 | 211 | 297 | 109 | 115 | 30 |
| S2 | 1724 | 531 | 1291 | 280 | 375 | 73 | 389 | 190 | 13 |
| S3 | 2423 | 348 | 807 | 453 | 304 | 224 | 138 | 149 | 60 |
| S4 | 1541 | 693 | 494 | 114 | 348 | 468 | 144 | 667 | 17 |
| S5 | 2195 | 1124 | 587 | 401 | 109 | 182 | 140 | 108 | 63 |
| S6 | 2524 | 1338 | 256 | 197 | 64 | 132 | 289 | 97 | 58 |
| S7 | 2156 | 418 | 986 | 367 | 236 | 121 | 98 | 189 | 45 |
| S8 | 2514 | 510 | 773 | 291 | 369 | 242 | 96 | 163 | 34 |
| S9 | 2309 | 402 | 749 | 359 | 317 | 185 | 223 | 101 | 39 |
| S10 | 2539 | 361 | 333 | 256 | 165 | 327 | 199 | 54 | 41 |

**Table S11.** ITH Scores of cell types across samples.

| **ID** | **B** | **NKT** | **Fibro** | **Macro** | **T** | **Neuo** | **Muscle** | **Plasma** | **Endo** |
| --- | --- | --- | --- | --- | --- | --- | --- | --- | --- |
| S1 | 21.62 | 17.81 | 27.03 | 24.60 | 275 | 27.60 | 37.56 | 26.95 | 18.83 |
| S2 | 179 | 13.16 | 16.63 | 24.86 | 17.57 | 16.55 | 16.97 | 14.34 | 14.17 |
| S3 | 17.77 | 15.43 | 25.70 | 15.78 | 23.60 | 19.57 | 21.56 | 17.05 | 15.58 |
| S4 | 20.53 | 179 | 37.49 | 17.53 | 23.51 | 29.44 | 28.38 | 224 | 18.56 |
| S5 | 21.34 | 12.77 | 26.91 | 25.74 | 28.05 | 21.63 | 27.57 | 18.64 | 22.91 |
| S6 | 16.07 | 12.58 | 20.65 | 21.07 | 33.80 | 13.48 | 34.61 | 16.96 | 16.60 |
| S7 | 13.87 | 17.12 | 18.01 | 14.34 | 17.58 | 27.07 | 19.92 | 16.34 | 136 |
| S8 | 12.62 | 10.85 | 16.95 | 15.31 | 18.02 | 12.59 | 12.82 | 13.78 | 12.94 |
| S9 | 18.47 | 17.69 | 18.80 | 14.56 | 16.16 | 14.79 | 29.50 | 16.17 | 16.05 |
| S10 | 17.37 | 16.45 | 35.93 | 21.68 | 21.41 | 20.96 | 34.15 | 15.99 | 16.62 |
